# Mixed-Length Multivariate Covalent Organic Framework for Combined Near-Infrared Photodynamic Therapy and Drug Delivery

**DOI:** 10.1021/jacs.5c07787

**Published:** 2025-09-08

**Authors:** Andrés Rodríguez-Camargo, Erdost Yildiz, Diego Juela, Felix Richard Fischer, Daniel Graf, Bibhuti Bhusan Rath, Christian Ochsenfeld, Matthias Bauer, Metin Sitti, Liang Yao, Bettina V. Lotsch

**Affiliations:** † Nanochemistry Department, 28326 Max Planck Institute for Solid State Research, Heisenbergstraße 1, 70569 Stuttgart, Germany; ‡ Department of Chemistry, University of Stuttgart, Pfaffenwaldring 55, 70569 Stuttgart, Germany; § Physical Intelligence Department, 28325 Max Planck Institute for Intelligent Systems, Heisenbergstraße 3, 70569 Stuttgart, Germany; ∥ Department of Chemistry, 9183 Ludwig-Maximilian University of Munich (LMU), Munich 81377, Germany; ⊥ Department of Chemistry and Center for Sustainable Systems Design (CSSD), 26578 University of Paderborn, Warburger 100, D-33098 Paderborn, Germany; # College of Engineering, Koc University, Rumelifeneri, 34450 Sarıyer/Istanbul, Turkey; ∇ State Key Laboratory of Luminescent Materials and Devices, Institute of Polymer Optoelectronic Materials and Devices, Guangdong Basic Research Center of Excellence for Energy and Information Polymer Materials, 26467 South China University of Technology, Guangzhou 510640, P. R. China

## Abstract

Covalent organic frameworks (COFs) have been emerging as versatile reticular materials due to their tunable structures and functionalities, enabled by precise molecular engineering at the atomic level. While the integration of multiple components into COFs has substantially expanded their structural complexity, the strategic engineering of diverse functionalities within a single framework *via* the random distribution of linkers with varying lengths remains largely unexplored. Here, we report a series of highly crystalline mixed-length multivariate COFs synthesized using azobenzene and bipyridine as linkers, where tuning the ratio of linkers and incorporating palladium effectively modulates the balance between near-infrared (NIR) light absorption and catalytic sites for NIR-generation of hydrogen peroxide (H_2_O_2_). Capitalizing on the deep tissue penetration of NIR light and the generated H_2_O_2_ as reactive oxygen species, as a proof of concept, the optimal mixed-length multivariate COF reduces breast cancer cell viability by almost 90% after 1 h of irradiation in a combined *in vitro* photodynamic therapy and drug delivery.

## Graphical abstract

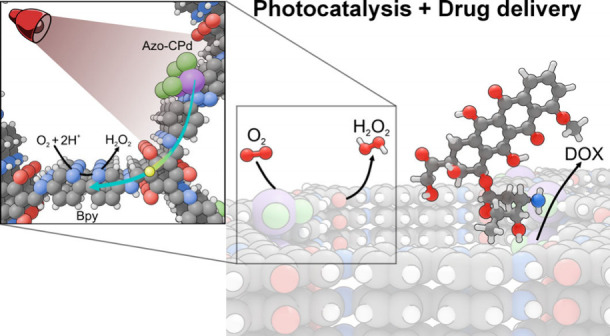

## Introduction

Covalent organic frameworks (COFs) are extended, molecularly precise crystalline solids with tunable porosity, constructed by covalent interconnections of organic building blocks. Since the first report of COFs in 2005,^1^ the design and synthesis of novel functional COFs have become central foci of reticular chemistry. Typically, COFs are synthesized using a minimal set of linkers—usually one or two—to construct periodic and symmetric framework structures. In the past decades, significant progress has been made in the development of multivariate COFs, which expand the diversity of COFs, typically through the incorporation of bifunctional linkers of uniform length, linkers with differing lengths in 1:2 and 2:1 ratios,^2^ and combinations of tetratopic, tritopic, and ditopic linkers.^3^ Challenging the constraint of uniform linker lengths and fixed linker ratios in constructing ordered frameworks could greatly enhance the structural diversity of COFs, paving the way for novel designs and advancing their molecular-level tunability. Therefore, efforts to introduce a random distribution of linkers with varying lengths within a single lattice, akin to the formation of inorganic solid solutions, have only recently emerged. COF solid solutions can thus be understood as a subcategory of multivariate COFs that is characterized by a random distribution of linkers sharing the same crystal lattice, with proportions varying continuously within certain limits.^4^ To account for this relationship between multivariate COFs and COF solid solutions, while emphasizing the fact that also linkers with variable lengths can be accommodated across an extended solid solution range, we refer to the latter as mixed-length multivariate COFs (ML multivariate) in the following.^5–7^ Indeed, ML multivariate COFs provide the unique opportunity to finely tune the lattice parameters by continuously varying the linker ratios, thereby introducing a broader range of structural complexity, while offering a simple and highly effective approach for integrating multiple and possibly synergistic functionalities into a single-phase COF. Dichtel and co-workers demonstrated the first synthesis of ML multivariate COF based on an imine linkage, revealing that ML multivariate COFs are formed specifically when all the linkers are present as monomeric species at the start of the polymerization, with framework strain playing a crucial role in stabilizing and developing the COF structure.^5^ Suzuki et al. investigated the synthesis of 12 boronic-acid-derived ML multivariate COFs, finding that flexible linkers and larger lattice parameters facilitate their formation and that the addition of a third component enables the creation of single-phase frameworks even when the node-linker pair cannot form a binary COF.^6^ However, despite these initial advancements, further development of functional ML multivariate COFs, as well as synthetic strategies to preserve high crystallinity while continuously varying the linker ratio, remains crucial to realize the full potential of this unique class of materials. Moreover, most reports on ML multivariate COFs have focused primarily on their synthesis, while their application and rational design for targeted functions—particularly those leveraging the integration of multiple, synergistic functionalities—remain largely unexplored.

A promising application of multifunctional ML multivariate COFs is in photodynamic therapy (PDT), where the integration of light absorption properties, catalytic activity for reactive oxygen species (ROS) generation, and drug delivery capabilities can offer enhanced therapeutic outcomes. Indeed, PDT has garnered increasing attention as an oncological intervention tool due to its noninvasive nature and precise spatial targeting, which contribute to minimal recurrence, reduced side effects, and the avoidance of cumulative radiation exposure. Achieving effective PDT requires the development of a photocatalyst that efficiently generates ROS upon light excitation, thereby enabling targeted cancer cell elimination. To maximize tissue penetration, an ideal photocatalyst should possess the capability to absorb near-infrared (NIR) light in the range of 760 to 1000 nm, ensuring deeper light penetration.^8,9^ Furthermore, it is crucial for the photocatalyst to exhibit high efficacy in generating ROS through a photocatalytic process. Moreover, using porous materials as photocatalysts offers the opportunity to load targeted molecules for drug delivery, enabling a combined therapeutic effect where PDT and drug release occur simultaneously. Although a variety of porous materials, such as metal–organic frameworks (MOFs) and COFs, have been explored as the light harvesters for PDT,^10–12^ the precise modulation of NIR light absorption and catalytic properties within a single porous photocatalytic system—capable of simultaneously carrying targeted molecules for drug delivery—has not yet been achieved.

To address this challenge, we here present a ML multivariate COF system that is an effective photocatalyst in PDT of breast cancer. This ML multivariate COF system consists of a *C*_3_-symmetric linker, 1,3,5-triformylphloroglucinol (*Tp*), and two *C*_2_-symmetric linkers with different lengths: cyclopalladated azobenzene (*Azo*) serving as a near-infrared light absorber, and a bipyridine (*Bpy*) based linker acting as a catalytic site for H_2_O_2_ production. Under optimized synthetic conditions, the resulting ML multivariate COFs, denoted as TpAzo_1–*x*_Bpy_*x*_ COFs (where x = 0.0, 0.3, 0.5, 0.7, and 1.0, as shown in Figure 1), retain high crystallinity when varying the *Azo*/*Bpy* ratio, and display a linear correlation between the linkers ratio and lattice size, demonstrating that *Azo* and *Bpy* are randomly distributed in a hexagonal lattice, akin to the situation of inorganic solid solutions. The modulation of near-infrared light absorption and catalytic sites enables the ML multivariate COF to achieve optimal H_2_O_2_ production of 504.1 ± 8.5 μmol g^–1^ h^–1^ under irradiation at 810 nm in the absence of any sacrificial agent. Leveraging the excellent ROS generation under the illumination of near-infrared light and the drug delivery capacity due to the suitable pore size, as a proof of concept, the optimal ML multivariate COF was further applied in combined PDT with pharmaceutical treatment for breast cancer *in vitro*.

**Figure 1:**
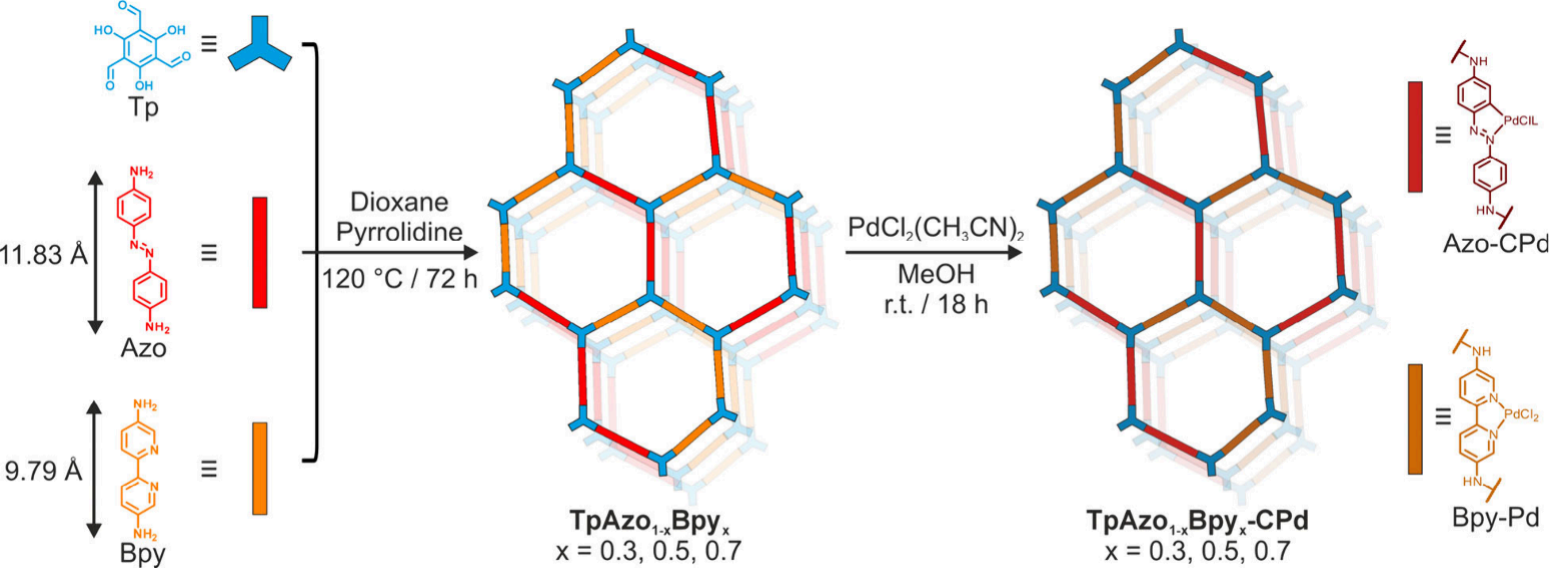
Schematic representation of TpAzo_1–*x*_Bpy_*x*_ COFs and their palladation.

## Results and Discussion

The syntheses of all studied COFs were carried out under similar solvothermal reaction conditions, using 1,4-dioxane as the reaction solvent and pyrrolidine as the condensation catalyst.^13^ Cyclopalladation was introduced to the *Azo* linker *via* a postsynthetic procedure that we recently developed (Figure 1).^14^ The two selected *C*_2_-symmetric linkers, *Azo* and *Bpy*, have lengths of 11.83 Å and 9.79 Å, respectively, as depicted in Figure 1. The direct condensation reactions between *Tp* and *Azo*, or between *Tp* and *Bpy*, result in the formation of crystalline TpAzo and TpBpy COFs, respectively. Both TpAzo and TpBpy COFs show five prominent diffraction peaks in their powder X-ray diffraction (PXRD) patterns, as shown in Figure 2a, which can be assigned to the 100, 110, 200, 210, and 001 reflections and indexed on hexagonal cells in space group *P6/m* (Figures S1 and S2). The ML multivariate COFs, TpAzo_1–*x*_Bpy_*x*_, were synthesized using mixed *Azo*/*Bpy* linkers while maintaining a constant total amine: aldehyde ratio during the condensation reaction. The five prominent Bragg diffraction peaks observed in the PXRD patterns of TpAzo and TpBpy are retained in the ML multivariate COFs, indicating the preservation of crystallinity. Moreover, as the *Bpy* content increases, the diffraction peaks corresponding to the *ab* plane systematically shift to higher 2θ values, while locating between those of TpBpy and TpAzo COFs (Figures 2b and 2c). Meanwhile, the 001 diffraction peak, associated with the interlayer stacking, remains at the same position (Figure 2d). The observations align with a continuous lattice contraction as a result of the increasing *Bpy* content (Figure 2e), following Vegard’s law and consistent with the shorter length of *Bpy* leading to a reduced lattice size, and suggest that the 2D topology of TpBpy and TpAzo COFs is preserved in TpAzo_1–*x*_Bpy_*x*_. To rule out the possibility that the diffraction peaks of TpAzo_1–*x*_Bpy_*x*_ arise from a physical mixture of TpBpy and TpAzo phases, PXRD measurement was performed on a mechanically mixed sample of TpBpy and TpAzo COFs. As shown in Figure S3, the PXRD pattern of this physical mixture exhibits two distinct 100 diffraction peaks, which are clearly different from those observed for TpAzo_1–*x*_Bpy_*x*_, thereby confirming the single-phase nature of the ML multivariate COFs. We also note that the crystallinity of TpAzo_1–*x*_Bpy_*x*_ is influenced by the synthetic procedure. The following is a comparison of synthetic Methods 1 and 2, in which all experimental details are preserved except for the order of addition of the linkers. When *Azo* and *Bpy* linkers were first dissolved to form a homogeneous solution before the addition of *Tp* (Method 1 in Figure S4), a linear correlation is observed between the *Bpy/Azo* ratio and the full width at half-maximum (FWHM) of the 100 reflections in the PXRD pattern of TpAzo_1–*x*_Bpy_*x*_ (Figure 2f). This result indicates that high crystallinity is obtained for TpAzo_1–*x*_Bpy_*x*_ by decreasing the *Bpy/Azo* ratio. However, when all three linkers were initially mixed in the solid state and subsequently dioxane was added as the reaction solvent (Method 2 in Figure S4), the resulting TpAzo_1–*x*_Bpy_*x*_ COFs exhibited significantly broader 100 reflections in the PXRD patterns (Figure S5), suggesting reduced crystallinity. We attribute this difference to the different nucleation processes during the initial polymerization stages. In Method 2, the extremely fast reaction of *Tp* and the inhomogeneous mixing of *Azo* and *Bpy* linkers promote the growth of uneven domains and disordered polymers, which are difficult to transform into a crystalline framework due to the limited reversibility of the *β*-ketoenamine linkage. In contrast, Method 1 facilitates the formation of a well-ordered ML multivariate COFs, since all linkers are fully dissolved prior to the reaction.^15^ Given the better crystallinity, TpAzo_1–*x*_Bpy_*x*_ COFs were prepared by Method 1 in the following sections.

**Figure 2:**
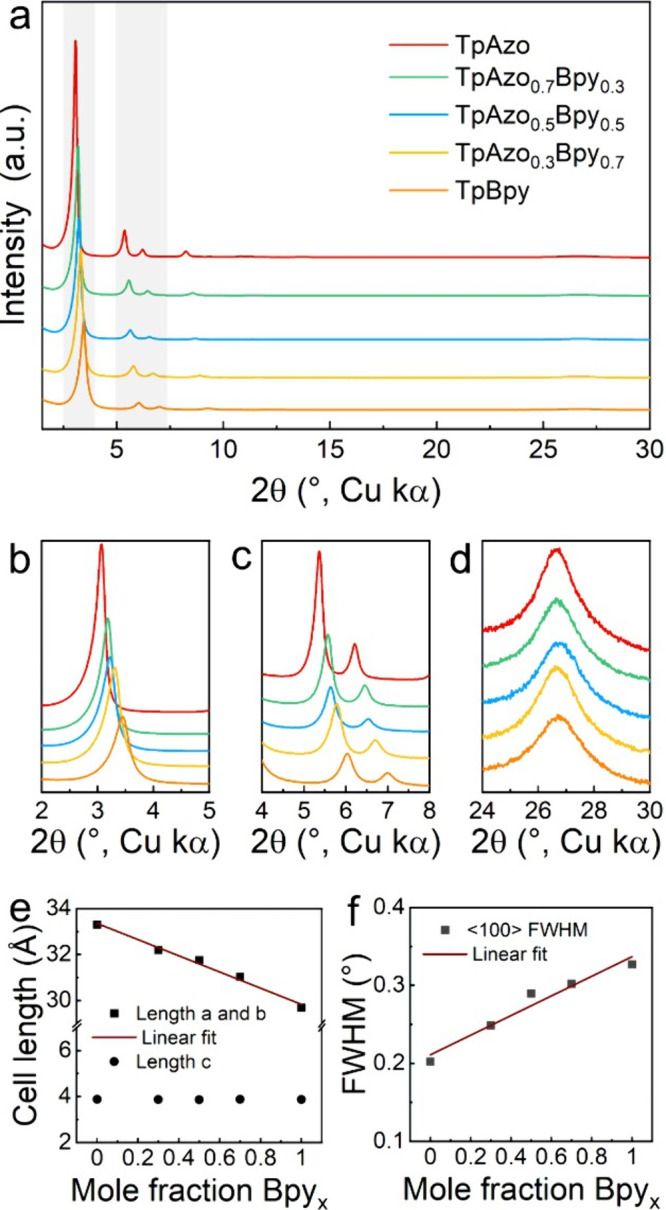
Structure analysis of TpAzo_1–*x*_Bpy_*x*_ COFs. PXRD patterns (a, the shaded areas highlight the 100, 110, and 200 reflections in TpAzo_1–*x*_Bpy_*x*_ COFs) and a close-up of the region corresponding to the 100 (b), 110, and 200 (c), and 001 (d) reflections of the TpAzo_1–*x*_Bpy_*x*_ COFs. Correlation between *Bpy* mole fraction and lattice parameters (e), and FWHM of the 100 reflection as a descriptor of the crystallinity of TpAzo_1–*x*_Bpy_*x*_ COFs.

Next, solid-state nuclear magnetic resonance (ssNMR) and liquid NMR of the digested samples were carried out to investigate the composition of the TpAzo_1–*x*_Bpy_*x*_ COFs. Overall, ^13^C-ssNMR showed the characteristic aromatic backbone signals in the range of 105 ppm to 150 ppm and the carbonyl signal of the keto-enamine form at 184 ppm (full signal assignment in Figures S6–S8).^16^ Specifically, as the molar ratio of the *Bpy* linker increases for TpAzo_1–*x*_Bpy_*x*_ COFs, the signal assigned to the aromatic ring carbon in the *Azo* linker at 113 ppm decreases, while the signal of the carbon in the *Bpy* aromatic ring at 135 ppm increases (shaded areas in Figure 3a). Additionally, chemical digestion of the TpAzo_1–*x*_Bpy_*x*_ COFs was conducted to determine the actual ratio between *Azo* and *Bpy* linkers. The digestion of β-ketoenamine-linked COFs presents an enormous challenge due to their remarkable stability in highly acidic and basic solutions.^15^ Nevertheless, we found that methylamine (MeNH_2_) can be used as an efficient digestion agent for *Tp*-based COFs (experimental details shown in the Supporting Information). The ^1^H NMR signals corresponding to the isolated *Azo* and *Bpy* linkers of the digested samples were analyzed in D_2_O (assignment in Figures S9–S11). The actual linker mole fraction in the TpAzo_1–*x*_Bpy_*x*_ COFs was estimated by calculating the integrals of the signals at 6.54 and 6.94 ppm from the *Azo* and *Bpy* linkers, respectively (shaded area in Figure 3b). Therefore, the proximity to 1.0 of the linear correlation slope between the expected and estimated linker mole fraction (0.992 ± 0.047) suggested that both *Azo* and *Bpy* linkers exhibit similar reactivity toward COF formation and preclude linker segregation for the synthesis of Method 1 (Figure 3c). Additionally, a trend was found in the Fourier transform infrared (FT-IR) spectra, where a characteristic band at 1391 cm^–1^, corresponding to the aromatic fingerprint of the *Bpy* linker, increases with the increasing *Bpy*/*Azo* ratio, while a band at 1555 cm^–1^, corresponding to the aromatic fingerprint of the *Azo* linker, decreases (Figure S12). The porosity of the COFs was investigated by N_2_ sorption isotherms (Figure 3d). In all the studied cases, the COFs present typical isotherms of mesoporous materials with an inflection at the low relative pressure range (around 0.1 P P_0_^–1^). The pore size distribution (PSD) calculated from N_2_ isotherms shows a monomodal distribution (Figure 3e). A linear correlation as a function of the *Bpy* mole ratio is observed for the PSD maxima (Figure 3f), in line with the lattice change indicated by the PXRD patterns and further proving the formation of TpAzo_1–*x*_Bpy_*x*_ ML multivariate COFs. In the Brunauer–Emmett–Teller (BET) surface areas of the studied COFs, a correlation with the *Bpy* mole fraction was not observed (Figure S14).

**Figure 3:**
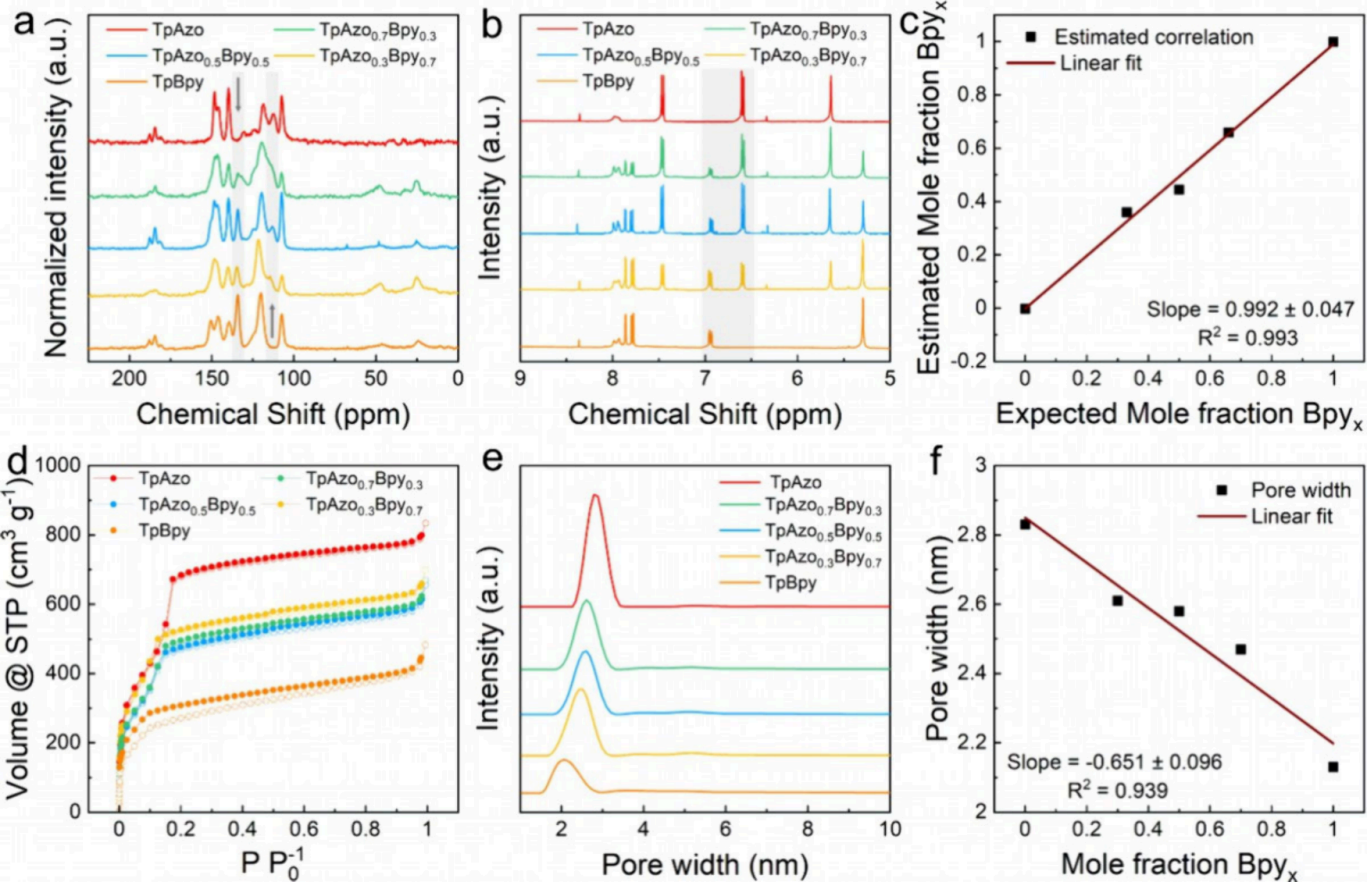
Characterization of TpAzo_1–*x*_Bpy_*x*_ COFs. ^13^C solid-state NMR (a, the shaded areas highlight the analyzed signals at 113 and 145 ppm), digested NMR (b, the shaded areas highlight the integrated signals at 6.54 and 6.94 ppm), and correlation between the expected and estimated mole fraction of *Bpy* (c) of TpAzo_1–*x*_Bpy_*x*_ COFs. N_2_ sorption isotherms at 77 K (d), pore size distributions (e), and correlation between the pore size distribution maximum and *Bpy* mole fraction (f) of TpAzo_1–*x*_Bpy_*x*_ COFs.

Next, we introduced atomic Pd *via* a cyclopalladation reaction into the *Azo* group of TpAzo_1–*x*_Bpy_*x*_ COFs *via* a postsynthetic cyclopalladation reaction (details provided in the Supporting Information), and the resulting ML multivariate COFs are denoted as TpAzo_1–*x*_Bpy_*x*_-CPd. The successful incorporation of palladacycles in TpAzo_1–*x*_Bpy_*x*_-CPd was confirmed by a series of characterizations, taking TpAzo_0.5_Bpy_0.5_-CPd COF as an example. First, inductively coupled plasma optical emission spectroscopy (ICP-OES) measurements show that all TpAzo_1–*x*_Bpy_*x*_-CPd COFs contain a considerable amount of palladium, ranging from 9.4 wt % to 18.4 wt % (Table S1). Transmission electron microscopy (TEM) images show no evidence of Pd nanoparticles, and the crystallinity is retained after incorporating palladacycles in TpAzo_0.5_Bpy_0.5_-CPd COF (Figures 4a and S15), which is further substantiated by PXRD patterns (Figure S16). Scanning transmission electron microscopy characterization coupled with energy-dispersive X-ray spectroscopy (STEM-EDX) indicates a homogeneous Pd distribution in TpAzo_0.5_Bpy_0.5_-CPd COF with the presence of chloride as the ligand attached to the palladacycles (Figure 4b). X-ray photoelectron spectroscopy (XPS) data further verify that palladium exists in TpAzo_0.5_Bpy_0.5_-CPd COF in the form of Pd­(II) ions, rather than metallic Pd(0), as indicated by the characteristic 3*d*_5/2_ and 3*d*_3/2_ core levels of Pd­(II) ions at 338.2 and 343.5 eV, respectively (Figure S17).^17^ Additionally, ^13^C solid-state NMR characterization confirms the formation of C–Pd bonds, evidenced by the aromatic carbon signal at 159 ppm, which becomes more pronounced as the mole fraction of the *Azo* linker increases (shaded area in Figure 4c).^18^ Liquid NMR spectra of the digested TpAzo_0.5_Bpy_0.5_-CPd by MeNH_2_ show signals at 7.11 and 6.24 ppm, corresponding to the aromatic hydrogen atoms in the cyclopalladated *Azo* linker (shaded area in Figure 4d).^19^

**Figure 4:**
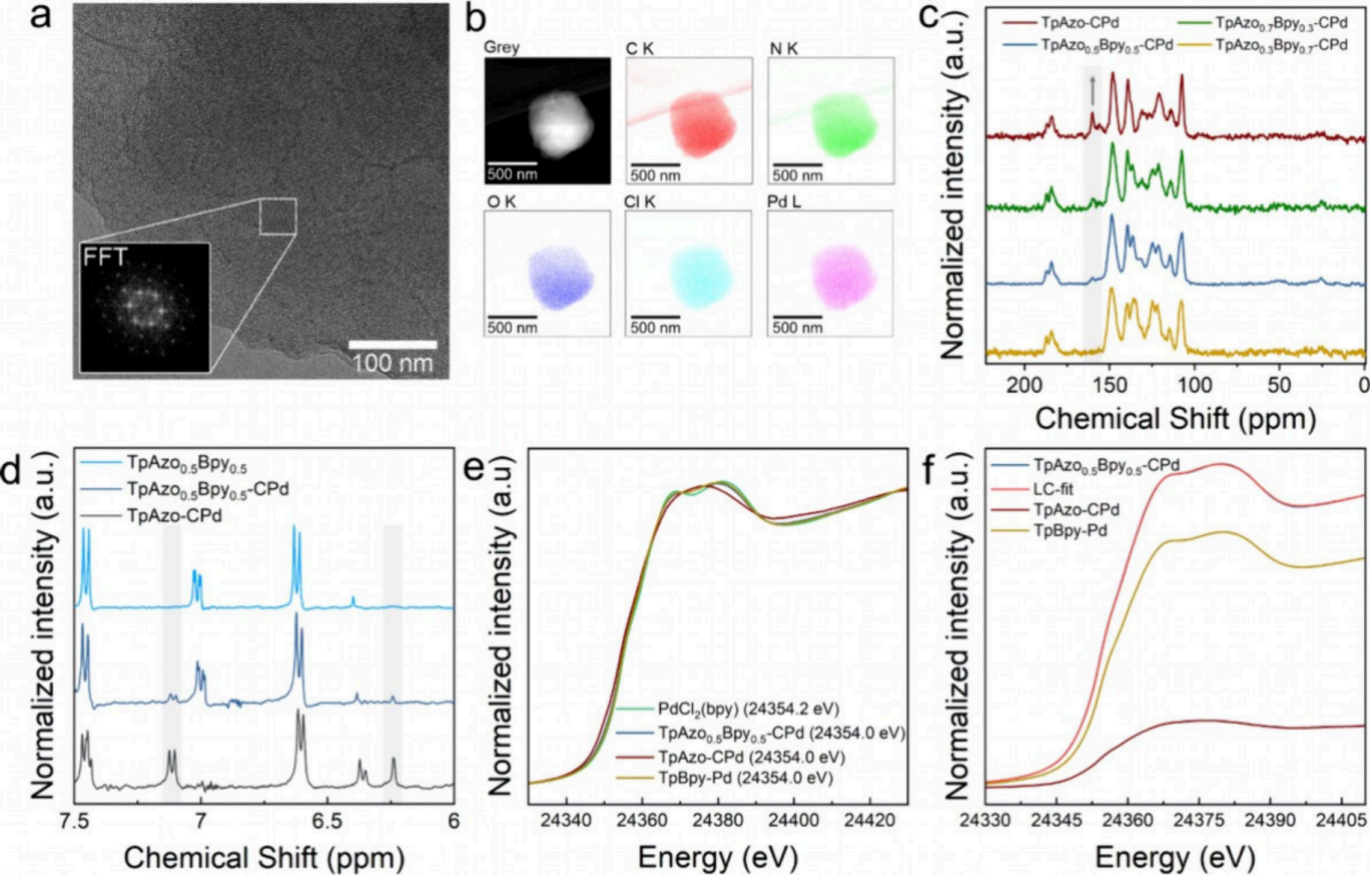
Characterization of palladated TpAzo_1–*x*_Bpy_*x*_-CPd COFs. a) TEM image of TpAzo_0.5_Bpy_0.5_-CPd. The inset shows the Fast-Fourier transformation (FFT) of the selected area. b) STEM-EDX elemental mapping analysis of TpAzo_0.5_Bpy_0.5_-CPd COF as an example of palladium distribution along the COF particle. c) ^13^C solid-state NMR spectra of TpAzo_1–*x*_Bpy_*x*_-CPd COFs (the shaded area highlights the signals at 159 ppm assigned to the C–Pd atoms). d) ^1^H-NMR of digested TpAzo_0.5_Bpy_0.5_, TpAzo_0.5_Bpy_0.5_-CPd, and TpAzo-CPd COFs to confirm the cyclopalladation of *Azo* linker in TpAzo_0.5_Bpy_0.5_-CPd COF (the shaded areas highlight the analyzed signals at 6.24 and 7.11 ppm). e) Experimental XANES spectra of PdCl_2_(bpy), TpAzo_0.5_Bpy_0.5_-CPd, TpAzo-CPd and TpBpy-Pd with absorption edge energies *E*_0_ in brackets. f) LC-XANES-fit for the spectrum of TpAzo_0.5_Bpy_0.5_-CPd using the XANES spectra of TpAzo-CPd and TpBpy-Pd as components.

Aside from anchoring on the *Azo* group, *Bpy* also possesses a strong coordination affinity for palladium. Therefore, X-ray absorption spectroscopy (XAS) measurements, including XANES (X-ray absorption near edge structure) and EXAFS (Extended X-ray absorption fine structure), were performed on TpAzo_0.5_Bpy_0.5_-CPd as a representative sample to differentiate the Pd coordination on *Bpy* and *Azo* residues. For this purpose, TpAzo-CPd, TpBpy-Pd, TpAzo_0.5_Bpy_0.5_-CPd COFs, and molecular PdCl_2_(bpy) were also analyzed as reference materials (Figure 4e,f). In contrast to other spectroscopic methods, XAS is independent of the aggregation state of the analyzed matter and represents the method of choice to obtain both electronic and structural information for homogeneous complexes and their immobilized analogues.^20–23^ The edge energy in a XANES spectrum correlates with the electron density and thus with the oxidation state of the excited atom. A comparison of the edge positions and absorption edge energies *E*_0_ of the investigated Pd samples reveals that in all cases, palladium is in the Pd­(II) oxidation state, in line with the results of XPS. Moreover, the XANES (Figure 4e) and Fourier-transformed (FT) EXAFS (Figure S28) spectra display substantial agreement in signal intensities among PdCl_2_(bpy), TpBpy-Pd, and TpAzo_0.5_Bpy_0.5_-CPd, indicating considerable structural similarities. Thus, together with the spectroscopic evidence shown above (Figure 4c and 4d), the coexistence of both species—cyclopalladation of *Azo* and palladation of *Bpy*—is proven. This observation is further validated by a linear combination (LC)-fit of the XANES spectrum of TpAzo_0.5_Bpy_0.5_-CPd using TpBpy-Pd and TpAzo-CPd as components (Figure 4f). Based on this fitting analysis, the coordination mode of Pd­(II) centers in TpBpy-Pd is predominant in TpAzo_0.5_Bpy_0.5_-CPd, accounting for approximately 79%, whereas the remaining 21% exhibits a bonding situation that is observed in TpAzo-CPd. Since the LC-XANES-fit with TpAzo-CPd and TpBpy-Pd summed up to 100%, the presence of a third Pd­(II)-coordination mode in TpAzo_0.5_Bpy_0.5_-CPd is ruled out. Further structural information about the local structure of Pd­(II) metal centers in TpAzo_0.5_Bpy_0.5_-CPd gained by extended X-ray absorption fine structure (EXAFS)-analysis is discussed in the Supporting Information (Table S4, Figure S29, Table S6).

The light absorption properties of TpAzo_1–*x*_Bpy_*x*_-CPd COFs were measured using visible-to-near-infrared spectroscopy (Vis-NIR spectroscopy). As previously reported, the cyclopalladation of Azo-containing COFs induces a considerable red-shift in the Vis-NIR spectra (Figure 5a), effectively extending light absorption into the NIR region.^14^ Notably, although the *Bpy* units in TpAzo_1–*x*_Bpy_*x*_-CPd COFs can also coordinate with Pd­(II) ions, TpBpy-Pd does not show intense absorption in the NIR region (Figure S30), which indicates cyclopalladated *Azo* units are the primary contributors to NIR light absorption. Accordingly, an increase in the *Bpy* fraction leads to a blue shift in the absorption and a corresponding increase in the optical band gap (Figure 5b). Since effective photosensitizers must possess suitable thermodynamic potentials to drive oxygen reduction for reactive oxygen species (ROS) generation, the conduction band minimum (CBM) of TpAzo_1–*x*_Bpy_*x*_-CPd COFs was determined *via* electrochemical measurements in a nonaqueous electrolyte (Figure S33). The results reveal that TpAzo_1–*x*_Bpy_*x*_-CPd COFs exhibit CBM values ranging from – 0.66 V to – 1.87 V vs NHE, which are more negative than the standard reduction potentials (E^0^) of oxygen reduction reactions, such as O_2_/O_2_^•–^ and O_2_/H_2_O_2_, suggesting that TpAzo_1–*x*_Bpy_*x*_-CPd COFs possess sufficient thermodynamic driving force for the oxygen reduction (Figure S34).^24^ In addition, valence band maximum (VBM) was estimated between 0.78 and 0.99 V vs NHE for TpAzo_1–*x*_Bpy_*x*_-CPd COFs. Subsequently, the photocatalytic performance of TpAzo_1–*x*_Bpy_*x*_-CPd ML multivariate COFs for hydrogen peroxide production, a crucial ROS, was evaluated under near-infrared light irradiation. Photocatalytic measurements were conducted by dispersing the COF in pure water and illuminating the system with an 810 nm LED for 1 h under continuous oxygen flow, and hydrogen peroxide was quantified by the triiodide colorimetric method.^25^ The resulting H_2_O_2_ production rate is summarized in Figure 5c. Among the studied COFs, TpAzo-CPd shows the lowest H_2_O_2_ production rate of 195.5 ± 4.9 μmol g^–1^ h^–1^, whereas the incorporation of *Bpy* units increases the H_2_O_2_ production rate. The highest H_2_O_2_ production rate of 504.1 ± 8.5 μmol g^–1^ h^–1^ is achieved for TpAzo_0.5_Bpy_0.5_-CPd, exhibiting a more than 1.5-fold increase and an apparent quantum yield of 0.66% at 810 nm in the absence of a sacrificial agent (Figure S35). It is noted that TpBpy-Pd shows an H_2_O_2_ production rate of 366.5 ± 6.8 μmol g^–1^ h^–1^, lower than that of TpAzo_0.5_Bpy_0.5_-CPd COF, highlighting the critical role of NIR light absorption facilitated by cyclopalladated *Azo* units in enhancing photocatalytic performance. Given that *Bpy* has been reported to act as efficient catalytic sites for H_2_O_2_ generation,^26^ a control COF with benzidine to replace *Bpy* was synthesized, denoted as TpAzo_0.5_Bz_0.5_-CPd (COF characterizations in Figures S36–S39). This control COF exhibits a lower H_2_O_2_ production rate (230.6 ± 5.5 μmol g^–1^ h^–1^), further supporting the role of *Bpy* as the catalytic sites in TpAzo_1–*x*_Bpy_*x*_-CPd COFs. The above results show that increasing the *Bpy/Azo* ratio in the COFs increases the number of active sites, but compromises the NIR light absorption driven by the Azo-CPd fragment. This establishes a clear interplay between the different linkers, where the optimal ratio of efficient active sites and NIR light absorption must be considered. Additionally, when TpAzo_0.5_Bpy_0.5_-CPd is illuminated in the absence of oxygen, the H_2_O_2_ production rate dramatically decreases to 36.3 ± 1.7 μmol g^–1^ h^–1^, confirming that oxygen serves as the reactant in H_2_O_2_ formation. These control experiments collectively demonstrate that the enhanced H_2_O_2_ production of TpAzo_0.5_Bpy_0.5_-CPd arises from the systematic modulation of NIR light absorption and catalytic activity for H_2_O_2_ generation.

**Figure 5:**
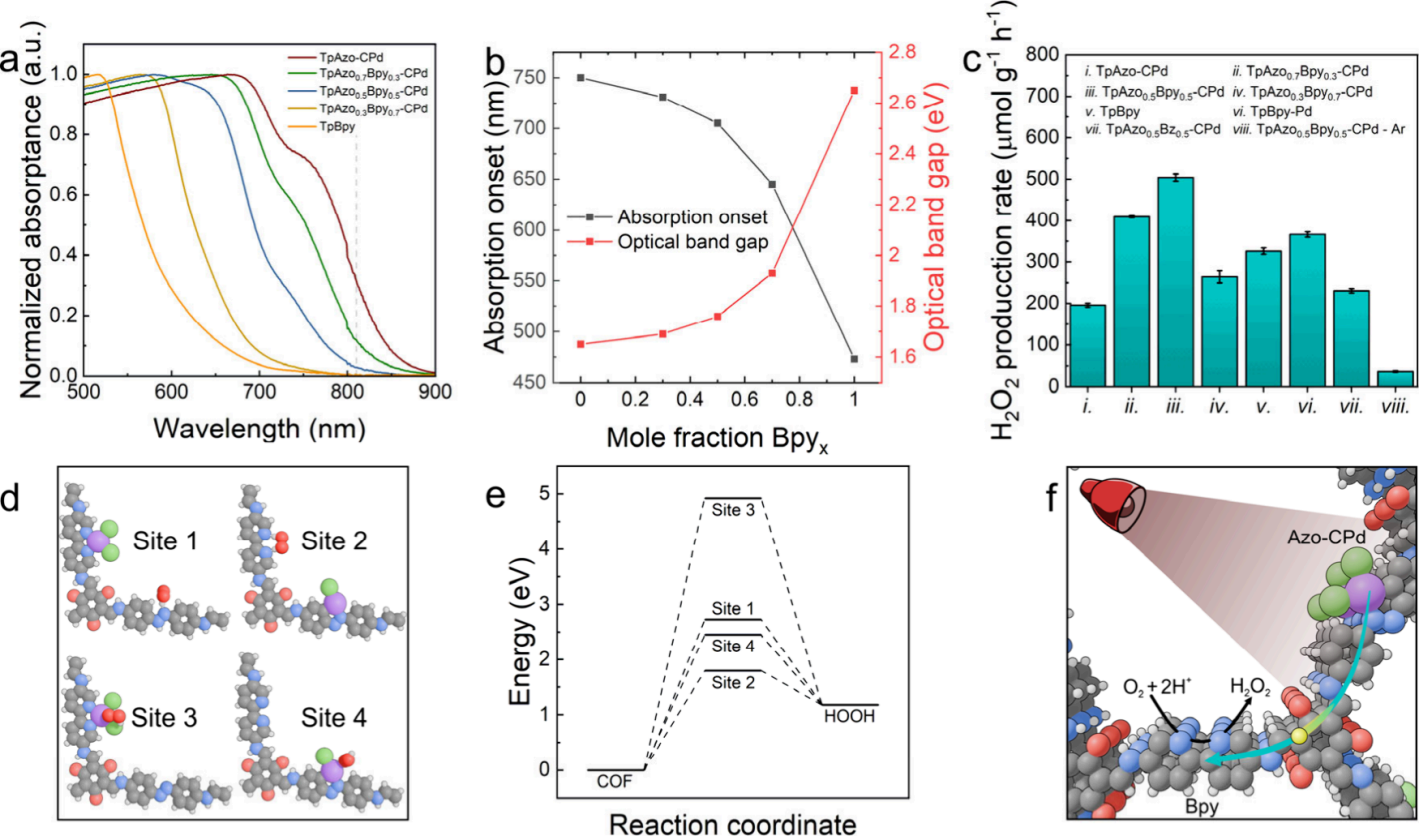
Photocatalytic study of TpAzo_1–*x*_Bpy_*x*_-CPd COFs. a) Vis-NIR spectra of TpAzo_1–*x*_Bpy_*x*_-CPd COFs. b) Correlation between the light absorption onset, optical band gap, and *Bpy* mole fraction. c) H_2_O_2_ production rate of different COFs. d) Proposed reactive sites for TpAzo_0.5_Bpy_0.5_-CPd COF. In gray, carbon atoms; white, hydrogen; red, oxygen; blue, nitrogen; purple, palladium; and green, chlorine. The calculated fragments consist of three stacked layers, but only one layer is shown for simplicity (for complete fragments, see Figure S45). e) Computed energy profiles of the reaction mechanisms corresponding to the four different reactive sites shown in Figure 5d. Note that all steps shown are based on fully balanced chemical equations, ensuring their comparability. f) Schematic representation of electron/electron–hole pair (yellow circle) migration from Azo-CPd residue to *Bpy* linker upon illumination for the ORR.

The H_2_O_2_ production pathway is explored by examining production rate changes upon introducing radical scavengers, including NaIO_3_, EDTA-2Na, and *p*-benzoquinone (*p*-BQ), which are selective scavengers for photogenerated electrons, holes and O_2_^•–^/^•^OOH radicals, respectively.^27,28^ Performing the photocatalytic reaction under the same conditions described above but adding 10 mM NaIO_3_ and 50 mM EDTA-2Na as scavengers results in a significant decrease in H_2_O_2_ production (as can be observed in Figure S41 using the peroxide test strips), confirming the involvement of photogenerated charges in H_2_O_2_ generation. In contrast, the incorporation of 1 mM *p*-BQ in the photocatalytic system leads to only a minor reduction in H_2_O_2_ production (Figure S43). This suggests that the two-step 1e^–^ pathway, where superoxide anion radicals serve as intermediates, is not the predominant reaction mechanism in this photocatalytic system. The primary reaction pathway thus follows a one-step 2e^–^ process.^28^ Electron paramagnetic resonance (EPR) measurements further support this conclusion, as no radical signal is detected by EPR after 10 min of irradiation (Figure S44) using 100 mM 2,2,6,6-tetramethylpiperidine (TEMP) and 5,5-dimethyl-1-pyrroline N-oxide (DMPO) as spin trapping agent for single oxygen and superoxide ion, respectively. To gain further insight into the reaction pathway and the reactive sites responsible for H_2_O_2_ production in TpAzo_1–*x*_Bpy_*x*_-CPd COFs, density functional theory (DFT) calculations were performed. The energies of four potential reactive sites (within the conductor-like screening model – COSMO) were compared, including the nitrogen atoms of the *Azo* residue, the nitrogen atoms of the *Bpy* unit, the Pd­(II) center coordinated on the *Bpy* moiety, and the Pd­(II) center in the Azo-CPd residue (denoted as site 1–4 in Figure 5d and Figure S45, respectively). The results demonstrate that the reactive site formed by the *Bpy* nitrogen atoms possesses the lowest energy, as shown in Figure 5e, pointing to its role as the primary catalytic site. Given that NIR light absorption is mediated by the Azo-CPd units while the catalytic sites are located at the *Bpy* units, we presume that a synergistic interplay between these two components takes place within the TpAzo_1–*x*_Bpy_*x*_-CPd frameworks. Thus, a plausible mechanism for H_2_O_2_ generation by TpAzo_1–*x*_Bpy_*x*_-CPd COFs involves NIR light absorption by the Azo-CPd unit, followed by electron or electron–hole pair migration to the Bpy moiety (Figure 5f). The nitrogen atoms in the reduced bipyridine unit then act as adsorption sites for molecular oxygen, where a one-step 2e^–^ process occurs to produce H_2_O_2_—consistent with the mechanism previously reported for the TpBpy COF.^26^ Further characterization of the photophysical properties, such as exciton lifetimes by photoluminescence spectroscopy, was not possible due to the lack of emission in these materials.

Given the superior performance of TpAzo_0.5_Bpy_0.5_-CPd COF in photocatalytic H_2_O_2_ production under NIR light compared to all tested samples (Figure 5c), we explored its potential for biomedical applications. As a proof of concept, we focused on carcinomas located near the skin, such as breast cancer. It is important to note that these experiments are at an early stage and require further development before being considered for clinical therapy. Due to the abundance of oxygen in the biological cells and the deep penetration of NIR light, TpAzo_0.5_Bpy_0.5_-CPd COF is expected to generate H_2_O_2_, a key ROS, *via* photocatalytic oxygen reduction within biological systems, thereby enabling PDT for *in vivo* breast cancer treatment.^29^ Furthermore, since the high porosity of TpAzo_0.5_Bpy_0.5_-CPd COF also allows for loading anticancer drugs (such as the well-known anticancer drug doxorubicin, DOX), a combined therapeutic strategy integrating PDT and drug delivery is demonstrated toward cancer treatment in the following (Figure 6a).

**Figure 6:**
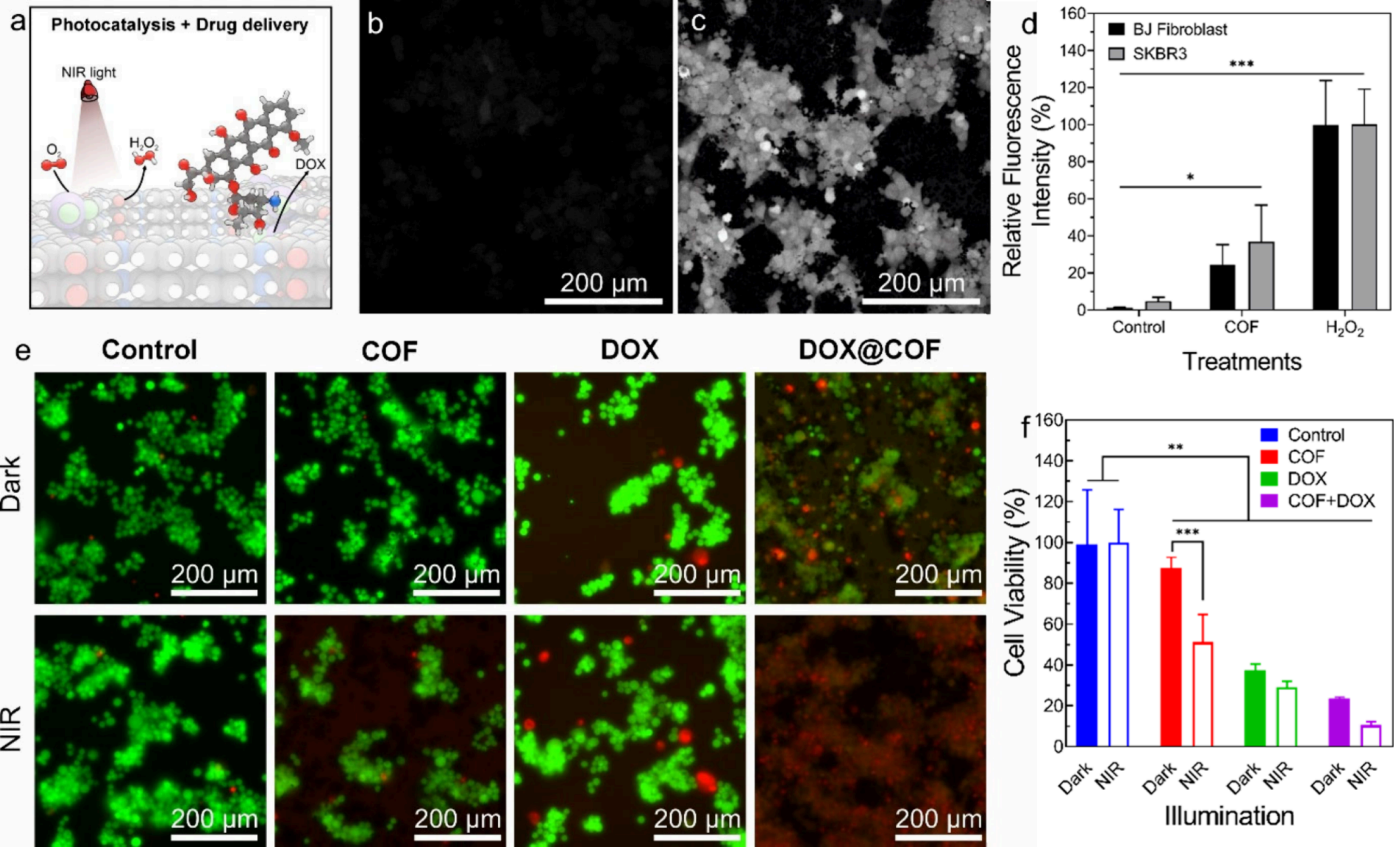
Combined phototherapy and drug delivery in TpAzo_0.5_Bpy_0.5_-CPd toward *in vitro* breast cancer treatment. a) Schematic representation of the cooperative effect between photocatalytic ORR to produce H_2_O_2_ and drug delivery. Gray atoms represent carbon, white hydrogen, red oxygen, blue nitrogen, green chloride, and purple palladium. H_2_DCFDA-based fluorescence assay for the *in vitro* ROS measurement in SKBR3 cell culture without (b) and with (c) TpAzo_0.5_Bpy_0.5_-CPd COF. d) Relative fluorescence intensities for H_2_DCFDA-based ROS measurement under various conditions of BJ and SKBR3 cell cultures. e) Calcein-AM-based and EthD-1-based fluorescent live (green) and dead (red) cell viability imaging of the SKBR3 cell culture for cell viability in dark and under 810 nm NIR illumination. f) Luciferase-based luminescence cell viability assay of the SKBR3 cell line under various conditions. * indicates *p* < 0.05, ** indicates *p* < 0.01, *** indicates *p* < 0.001, where “p” indicates statistical significancy.

Before *in vitro* breast cancer treatment, the biocompatibility of TpAzo_0.5_Bpy_0.5_-CPd COF with two different cell cultures was investigated: BJ cells, representing healthy fibroblasts surrounding the tumor, and SKBR3 cells, modeling breast cancer tumor cells. Using a Luciferase-based quantitative cell viability assay, the median lethal doses (LD_50_) of TpAzo_0.5_Bpy_0.5_-CPd COF are determined to be 453.9 μg mL^–1^ for BJ fibroblasts and 267.2 μg mL^–1^ for SKBR3 cell cultures (n = 4; Figure S46, where “n” indicates number of samples for each group). These results were further validated using Calcein-AM and EthD-1-based fluorescent live cell staining (n = 4; Figures S47 and S48). Based on these findings, a TpAzo_0.5_Bpy_0.5_-CPd COF concentration of 200 μg mL^–1^ was selected for subsequent experiments. ROS generation under cell culture conditions upon NIR irradiation *in vitro* was quantified using a fluorescence-based ROS detection method, specifically the 2′, 7′-dichlorodihydrofluorescein diacetate (H_2_DCFDA) assay, which shows a fluorescence intensity proportional to the ROS concentration.^30^ As shown in the qualitative analysis in Figures 6b and 6c, no fluorescence is observed in the absence of TpAzo_0.5_Bpy_0.5_-CPd COF after irradiation at 810 nm (n = 6; Figure 6b and Figure S49a). In contrast, when TpAzo_0.5_Bpy_0.5_-CPd COF is present, fluorescence is detected (n = 6; Figure 6c and Figure S49b), indicating that ROS generation is facilitated by TpAzo_0.5_Bpy_0.5_-CPd COF upon NIR light irradiation. Even more, TpAzo_0.5_Bpy_0.5_-CPd COF showed a relative fluorescence intensity of 36.9 ± 19.8% compared to the fluorescence intensity of a 100 μM H_2_O_2_ aqueous solution in the SKBR3 cell culture, which is 100.1 ± 19.0% (n = 6; Figure 6d). Subsequently, the cytotoxic effect of the photogenerated ROS in the breast cancer cells was studied, and cell viability was evaluated by Luciferase-based luminescence and Calcein-AM-based fluorescent live cell viability assays. Qualitative analysis using Calcein-AM staining (green fluorescence) shows that the number of live cells remains nearly unchanged in the presence of COF under dark conditions, although the COF slightly interferes with EthD-1 (red fluorescence) due to its absorbance close to the red visible light spectrum (Figures 6e, S50, and S50). However, after 1 h NIR irradiation, a noticeable decrease in cell viability is observed compared to the dark condition. Quantitative luminescence assay further confirms this effect, showing a reduction in cancer cell viability from 100.0 ± 16.1% in the control group to 51.2 ± 13.4% upon exposure to TpAzo_0.5_Bpy_0.5_-CPd COF under NIR irradiation (n = 6; Figure 6f). These results provide strong evidence of the desired phototherapeutic effect, likely correlated with the H_2_O_2_ generation by TpAzo_0.5_Bpy_0.5_-CPd COF from photocatalytic oxygen reduction. Moreover, DOX was loaded into TpAzo_0.5_Bpy_0.5_-CPd COF via physisorption as a chemotherapeutic agent to enable combined PDT and drug delivery. To optimize the DOX loading capacity, TpAzo_0.5_Bpy_0.5_-CPd COF was exposed to various DOX aqueous solutions, identifying an optimal ratio of 400 μg mL^–1^ DOX solution per 100 μg of COF, achieving a maximum DOX loading of 40.7 ± 10.0 wt % (Figure S50). After loading of DOX to the COF particles, drug release of TpAzo_0.5_Bpy_0.5_-CPd COF was investigated in various conditions, including various pH, H_2_O_2_, glutathione levels, and presence of NIR light, and the constant drug release from COF is demonstrated (Figures S53–56). The DOX-loaded TpAzo_0.5_Bpy_0.5_-CPd COF exhibits a significantly enhanced therapeutic effect, further reducing cell viability to 10.6 ± 1.7% under 810 nm illumination, compared to 37.3 ± 3.2% for free DOX in the dark. This NIR-induced photodynamic therapeutic effect of the COF under NIR exposure was also demonstrated in a breast cancer organoid model using Calcein-AM-based live-cell viability and immunofluorescence imaging methods­(Figures S57 and S58). The DOX-loaded COF treatment group under NIR exposure has lower viability and proliferation compared to the control group. NIR-induced ROS generation from COF has also increased the oxidized glutathione levels in the breast cancer cells (Figure S59). This pronounced reduction demonstrates the effectiveness of the combined therapy, highlighting the unique synergism between PDT and drug delivery.

## Conclusions

In summary, we have synthesized a series of multivariate COFs based on statistically distributed *Bpy* and *Azo* linkers, which form a ML multivariate COF series with a highly crystalline single-phase average structure and gradually tunable unit cell metrics and pore sizes. Site-isolated, single Pd­(II) ions rather than Pd NPs were successfully incorporated into the COF backbone, simultaneously forming azobenzene-based palladacycles and coordinative bonds with the *Bpy* linker. Both Azo-CPd and *Bpy* linkers act in tandem to produce H_2_O_2_ in a dye-sensitized catalytic scheme: While the Azo-CPd units act as photosensitizers, harvesting low-energy photons, the *Bpy* units provide catalytic sites for the photocatalytic ORR *via* a two-electron pathway. Indeed, by systematically tuning the *Bpy/Azo* ratio, we achieve an ideal balance between NIR light absorption and photocatalytic generation of H_2_O_2_, a potent reactive oxygen species (ROS). This example highlights the synergistic interplay of molecular building blocks for the photocatalytic generation of H_2_O_2_ in the NIR, which, together with the deep tissue penetration of NIR light, opens new avenues in photodynamic cancer therapy. To explore this possibility, H_2_O_2_ production under NIR light was combined with the drug delivery ability of the COF for the cooperative treatment of breast cancer. While covalent organic frameworks have been used for the photogeneration of H_2_O_2_ in previous studies, our work presents an example where synergies between two building blocks in a multivariate COF are leveraged for the combined photodynamic and pharmaceutical therapy of tumors in a biomedical setting.^31,32^ This work thus sets the stage for the rational design of NIR-responsive porous materials for theranostic applications and cooperative therapies based on drug delivery and photodynamic therapy, as shown here. As this study represents the first exploration of mixed-length multivariate COFs in biomedical applications, comprehensive *in vivo* investigations—including pharmacokinetics, metabolic pathways, and systemic toxicity—are beyond its current scope. The absence of such evaluations constitutes a key limitation of this work, which will be addressed in future studies aimed at translating these materials toward biomedical applications. To advance this concept toward clinical application, future studies should focus on optimizing COF morphology, controlling particle size, and evaluating performance in *in vivo* conditions.

## Supplementary Material



## Data Availability

All data that support the findings of this study are available in the Supporting Information of this article. A data set has been created with source data at 10.18419/DARUS-4837.

## Notes

Detailed materials and methods, additional experimental details, supplementary COF characterization, photocatalytic experiments, biomedical tests, Figures S1–S59, and Tables S1–S7 (PDF)
