# Supplementary material for: Mixed-Length Multivariate Covalent Organic Framework for Combined Near-Infrared Photodynamic Therapy and Drug Delivery
Source: J Am Chem Soc. 2025 Sep 8;147(37):33472–81. doi: 10.1021/jacs.5c07787 (PMC12447492; doi:10.1021/jacs.5c07787)
Supplement: Supplementary file 1 [file ja5c07787_si_001.pdf]

Mixed-Length Multivariate Covalent Organic Framework for Combined Near-Infrared Photodynamic Therapy and Drug Delivery

Andrés Rodríguez-Camargo[1,2], Erdost Yildiz[3], Diego Juela[1,2], Felix Richard Fischer[2], Daniel Graf[4], Bibhuti Bhusan Rath[1], Christian Ochsenfeld[4], Matthias Bauer[5], Metin Sitti[3,6], Liang Yao[7]*, Bettina V. Lotsch[1,2,4]*

[1]Nanochemistry Department
Max Planck Institute for Solid State Research
Heisenbergstraße 1, 70569 Stuttgart, Germany
E-mail: b.lotsch@fkf.mpg.de

[2]Department of Chemistry
University of Stuttgart
Pfaffenwaldring 55, 70569 Stuttgart, Germany

[3]Physical Intelligence Department
Max Planck Institute for Intelligent Systems
Heisenbergstraße 3, 70569 Stuttgart, Germany

[4]Department of Chemistry
Ludwig-Maximilian University of Munich (LMU)
Munich 81377, Germany

[5]Department of Chemistry and Center for Sustainable Systems Design (CSSD)
University of Paderborn
Warburger 100, D-33098 Paderborn, Germany

[6]College of Engineering
Koc University
Rumelifeneri, 34450 Sarıyer/Istanbul, Turkey

[7]State Key Laboratory of Luminescent Materials and Devices, Institute of Polymer Optoelectronic Materials and Devices, Guangdong Basic Research Center of Excellence for Energy and Information Polymer Materials
South China University of Technology
Guangzhou 510640, P. R. China
Email: liangyao@scut.edu.cn

Supporting information

Experimental methods

Chemicals

All chemicals were purchased from commercial suppliers without any further purification: 4,4'-Diaminoazobenzene (Azo, Thermo Scientific, 95%), benzidine (Bz, Sigma-Aldrich, 98%), 2,4,6-Triformylphloroglucinol (Tp, TCI, 98%), 1,4-dioxane (extra dry, Acros organics, 99.5%), tetrahydrofuran (THF, Carl-Roth, 99%), pyrrolidine (Py, Sigma-Aldrich, 99%), methanol (MeOH, Carl-Roth, 99.9%), bis(acetonitrile)dichloropalladium(II) ($PdCl_2(CH_3CN)_2$, Sigma-Aldrich, 99%), ethylenediaminetetraacetic acid disodium salt dehydrate (EDTA-2Na, Sigma-Aldrich, 99.0-101.0%), *p*-benzoquinone (BQ, Sigma-Aldrich, ≥98%), sodium iodate ($NaIO_3$, Sigma-Aldrich, ≥99%), *tert*-butyl alcohol (TBA, Sigma-Aldrich, ≥99%), hydrogen peroxide strips test (Sigma-Aldrich), 5,5-Dimethyl-1-pyrroline N-oxide (DMPO, Sigma-Aldrich), 2,2,6,6-Tetramethylpiperidine (TEMP, Sigma-Aldrich, ≥99%).

Characterization

Inductively coupled plasma optical atomic emission spectroscopy (ICP-OES): The palladium content was quantified using a Varian Vista-PRO instrument, which is a simultaneous ICP-OES spectrometer with axial plasma manufactured by Agilent Technologies. The analysis was performed with the aid of ICP-Expert software. For sample preparation, microwave digestion was carried out using a Discover SP-D system from CEM GmbH. The samples were dissolved in nitric acid (65%) and subjected to microwave digestion at 185 °C for 25 minutes, followed by dilution with double-distilled water.

Powder X-ray diffraction (PXRD): Powder X-ray Diffraction patterns were obtained at room temperature using a Stoe Stadi P diffractometer (Cu-Kα_1) equipped with a Ge(111) primary monochromator, following the Debye-Scherrer geometry, and a triple array of Mythen 1 K detectors (Dectris). Each sample was securely sealed within 1.0 mm glass capillaries, and the measurements were taken with rotation to enhance particle statistics.

Gas sorption measurements: Nitrogen sorption measurements were conducted at 77 K using a Quantachrome Instruments Autosorb iQ 3. The pore size distribution (PSD) was determined by employing the N_2 adsorption data at 77 K and applying the QSDFT model (cylindrical pores, adsorption branch) within ASiQwin software version 3.01. Prior to measurement, the COF samples were subjected to activation under high vacuum conditions at 120 °C for a duration of 12 hours. For the determination of the BET surface area, a specific pressure range (P/P_0 = 0.05–0.2) was selected.

Nuclear magnetic resonance spectroscopy (NMR): Liquid-state ^{1}H-NMR spectra of digested samples were acquired using a JEOL ECZ 400S 400 MHz spectrometer and the samples were prepared by mixing 300 µL of digested COF solution in $MeNH_2$ with 300 µL of dimethylsulfoxide-*d*6. Solid-state (ssNMR) spectra were obtained at room temperature using 2.5 mm OD ZrO_2 rotors on a Bruker Avance III 400 MHz spectrometer equipped with Bruker BL4 or BL2.5 double resonance MAS probes. Standard instrument library pulse sequences were applied, and chemical shifts were referenced relative to adamantane (^{13}C; 38.52 ppm and 29.47 ppm).

Fourier transform infrared spectroscopy (FT-IR): Spectra were obtained in attenuated total reflection (ATR) geometry using a PerkinElmer UATR Two apparatus equipped with a diamond crystal. These spectra were subjected to background correction. FT-IR data are presented with a wavenumber (cm^{-1}) scale.

Supercritical CO_2 activation: Drying of the COF samples with supercritical CO_2 was performed on a Leica EM CPD300 critical point dryer.

Vis-NIR absorptance spectroscopy (Vis-NIR): Vis-NIR spectra were acquired on a Cary 5000 spectrometer (referenced to barium sulphate) using an integration sphere in absorptance mode. Subsequently, the optical band gaps were estimated by Tauc plot assuming a direct transition.

UV-Vis absorption spectroscopy (UV-Vis): UV-Vis absorption spectra were recorded in an Agilent Technologies Cary 60 spectrometer using a quartz cuvette (1 cm path length).

Transmission electron microscopy (TEM): High-resolution-TEM (HR-TEM) images were collected using a Philips CM30 ST instrument operating at 300 kV with a LaB6 cathode. The samples were meticulously dried onto copper lacey carbon grids provided by Plano. Image analysis and fast Fourier transform (FFT) were performed with ImageJ software. Scanning TEM (STEM)

where performed at a JEOL ARM 200CF scanning transmission electron microscope equipped with a cold field emission electron source, a DCOR probe corrector (CEOS GmbH), and a 100 mm^2 JEOL Centurio EDX detector. Pathfinder Software from Thermo Fisher Scientific was used for EDX data analysis.

Zeta Potential Measurements: The zeta potential was determined using a Malvern nano Zs zetasizer. Dispersions of 2 mg mL^{-1} of COF in 10 mM aqueous NaCl were sonicated 10 min prior to zeta potential measurements.

X-ray photoelectron spectroscopy (XPS): XPS measurements were conducted using a Thermo Scientific Theta Probe system. A monochromatic X-ray source (Al-Kα, 1486.6 eV photon energy, 100 W operating power, 400 μm surface probe diameter) was employed to stimulate photoemission. The XPS spectra were captured at pass energy of 200 eV (survey scan, 1 eV step, 50 ms dwell time) with multiple pass energy steps (single peaks, snap scan mode with 500 frames, 1-second scan time per frame). To address peak shifts due to sample charging, a flood gun was used during the measurements. The binding energies of all peaks were calibrated against the adventitious sp^3 carbon reference peak at 284.8 eV. Background correction was applied using a smart background function. The peaks were then fitted with Voigt functions, comprising approximately 30% Lorentzian and 70% Gaussian components, using the ThermoAvantage software.

X-ray absorption spectroscopy (XAS): Samples were measured at the Palladium K-edge (24350 eV) in transmission mode at the P65 beamline (PETRA III, DESY). Measurements were carried out at room temperature and up to 1000 eV above the Pd K-edge. For energy selection, a Si(311) double crystal monochromator (DCM) was used with a resolving power of 1.4 x 10^{-4} resulting in an averaged experimental resolution at Pd K-edge of 3.4 eV. For energy calibration, a Pd(0) foil was measured simultaneously with both the homogeneous and immobilized Pd samples. The quantification of the absorption edge energy E_0 was performed by identifying the first inflection point in the XANES spectrum of the Pd foil. $PdCl_2$(bpy) was measured in the solid state as a self-supporting wafer using boron nitride as a binder. The concentration was calculated for an edge jump of 0.3. TpAzo-CPd, TpBpy-Pd, and $TpAzo_{0.5}Bpy_{0.5}$-CPd were measured as pure powder. Spectra showed no radiation damage. XANES- and EXAFS-analysis of the experimental spectra

was accomplished with the aid of the Demeter package.[1] Background subtraction and normalization of the XAS raw data as well as the LC-XANES-Fit were performed with the Athena software. EXAFS fitting was carried out using the Artemis software applying the full multiple scattering approach (FMS). During the multiple-parameter EXAFS fitting procedure, the parameters for each sample were fitted individually.

Electron paramagnetic resonance (EPR): EPR spectra were obtained using a BRUKER EMXnano. The experiments involved 2.0 mg of $TpAzo_{0.5}Bpy_{0.5}$-CPd COF in water (1 mL), following 5 minutes of sonication and 5 minutes of oxygenation. Before illumination with NIR LED (810 nm) for 10 minutes, 0.1 mmol of the specified spin trap was added.

Electrochemistry: Cyclic voltammetry measurements were conducted to determine the HOMO and LUMO energy levels of COFs. These measurements were performed using a WaveDriver 200 EIS Pine Bipotentiostat. The experimental setup involved a three-electrode configuration, with Ag/Ag^{+} serving as the reference electrode, Pt wire as the counter electrode, and a 1 cm x 1 cm carbon paper as the working electrode. First, for the preparation of the working electrode, COF ink was prepared by dispersing COF in ethanol (2mg/mL) and stirring overnight. Then, 75 μL of COF ink was dropcasted onto each side of a carbon paper electrode. Anhydrous acetonitrile with 0.1 M tetrabutylammonium hexafluorophosphate was used as an electrolyte under the Ar atmosphere. Before the measurement, the electrochemical cell was purged with argon for 10 min. Red-Ox onset potentials (E_{onset}) were determined from linear fits within the voltammograms and the potentials versus Fc/Fc^{+} ($E_{Fc/Fc+}$) were subsequently converted according to the previously established method.[2]

Quantum-chemical calculations: Following the approach outlined in Ref.[3], DFT calculations to investigate potential reaction sites and their corresponding reaction mechanisms were performed. For all calculations, the FermiONs++[4-6] program package developed in the Ochsenfeld group was employed; structure optimizations were conducted with the DL-FIND[7] library. The structure optimizations as well as the single point calculations were conducted using the PBEh-3c functional developed by Grimme and co-workers[8] in combination with the def2-mSVP basis set and the respective effective core potentials. In all calculations, the solvent was treated implicitly, using the Conductor-like Screening Model (COSMO)[9] with the dielectric constant set to $\varepsilon = 78.39$. For visualization, Avogadro[10] was employed.

Photocatalysis

H_2O_2 quantification: The quantification of H_2O_2 was conducted through a spectrophotometric method based on the triiodide procedure, as previously reported.[11] Specifically, 1 mL of filtered reaction solution (passed through a 0.22 µm hydrophilic PTFE filter) was mixed with 1 mL of a 0.4 M aqueous potassium iodide (KI) solution and 1 mL of an aqueous 0.1 M potassium hydrogen phthalate (KHP) solution. The resulting mixture was shielded from light for 30 minutes before UV-Vis absorption measurement. To establish a calibration curve, fresh solutions of KI and KHP were prepared weekly, and the curve points were generated using a commercial 30 wt% H_2O_2 solution and Milli-Q water.

H_2O_2 production: In a typical photocatalytic experiment, 5 mg of COF was sonicated in 10 mL of Milli-Q water for 10 min. Next, the dispersion was incorporated into a glass reactor with a quartz top window as we reported previously.[12] The COF dispersion was stirred (200 rpm) and purged with oxygen gas (100 mL/min) for 10 min before illumination. Finally, the reaction was continuously purged and stirred for 1 hour under top illumination using a Thorlabs 810 nm mounted LED (M810L5) at full power (1.6 mW cm^{-2}).

Kinetics of H_2O_2 production: In a typical photocatalytic experiment, 5 mg of COF was sonicated in 10 mL of Milli-Q water for 10 min. Subsequently, the dispersion was incorporated into a glass reactor with a quartz top window, stirred (200 rpm), and purged with oxygen gas (100 mL/min) for 30 min before illumination. The reactor was then sealed, and the flow of oxygen gas was stopped. The reaction was continuously stirred under illumination with an 810 nm LED. After a certain time, the stirring was stopped and the dispersion was kept undisturbed for two minutes to separate the aqueous phase (top) from the benzyl alcohol phase (bottom). Next, 0.7 mL of the aqueous phase containing H_2O_2 was taken with the aid of a syringe, needle, and PTFE filter. Subsequently, 0.7 mL of Mili-Q water was added back to the reaction to keep the reaction volume constant. Before restarting the illumination, the reactor was purged with O_2 gas for 10 min. This procedure was repeated after the completion of the kinetic experiment.

H_2O_2 production in the presence of scavengers: Photocatalytic experiments were conducted as described above, with the addition of specific scavengers to the catalytic medium to achieve final concentrations of 10 mM p-BQ, 10 mM $NaIO_3$, and 50 mM EDTA-2Na.

Cell culture and NIR illumination conditions: For cell culture experiments, SKBR3 human breast cancer (HTB-30, ATCC) and BJ human skin fibroblast (CRL-2522, ATCC) cell lines were

used. While SKBR3 cells were cultured in McCoy's 5A modified medium (16600082, Gibco) with 10% fetal bovine serum (FBS, A5256801, Gibco), BJ cells were cultured in Dulbecco's Modified Eagle Medium (DMEM, 11965092, Gibco) with 10% FBS in a 37 °C, 5% CO_2 incubator. 24 hours before all cell culture experiments, 30000 cells per well were seeded to glass bottom chambered cover slides (μ-slide 8-well, ibidi). NIR light was applied with the maximum intensity (1190 mW output power and 1.6 mW cm^{-2} maximum irradiance) of an 810 nm LED light source (M810L5, Thorlabs) inside a dark 37 °C, 5% CO_2 cell culture incubator.

Measurements of reactive oxygen species: The intracellular reactive oxygen species (ROS) were measured by the H_2DCFDA assay kit (D399, Molecular Probes, Invitrogen). After one hour of NIR illumination on SKBR3 and BJ cells, the cells were washed once with phosphate-buffered saline (PBS, 10010023, Gibco) and then incubated with 20 μM H2DCFDA in the medium for 45 minutes in the humidified cell culture incubator before imaging to allow H2DCFDA to enter the cells. One-hour incubation of cells with 100 μM H_2O_2 was used as the positive control group. After the H_2DCFDA incubation and washing with PBS in dark conditions, live cell fluorescence images were taken from the samples in the same light intensity, exposure, time point, and magnification with a confocal fluorescence microscope (SP8 DMi8, Leica), and average fluorescence intensity from segmented cell areas were measured with a custom code in MATLAB software (R2021a, MathWorks). Relative fluorescence intensity was calculated with cells without any treatment (negative control) and cells with a one-hour H_2O_2 treatment (positive control) groups. The ROS production levels in the positive control group were accepted as 100% in the relative fluorescence intensity comparison.[13]

Measurement of glutathione levels in cell culture: The oxidized and total glutathione measurements from cell culture experiments were made with the GSH/GSSG-Glo Assay Kit (Promega). SKBR3 cells were seeded in a 96-well plate at a density of 5,000 cells per well and cultured overnight. After treatment under various conditions, the growth medium was replaced with a physiological buffer containing the compound. Total glutathione is measured using a total glutathione lysis reagent, which includes both reduced (GSH) and oxidized glutathione (GSSG). Oxidized glutathione is measured using an oxidized glutathione lysis reagent. The plate is agitated and incubated for five minutes at room temperature. Then, the Luciferin Generation Reagent is added, and the plate is incubated for 30 minutes. Finally, the luciferin detection reagent is

introduced. After a 15-minute incubation, luminescence is measured using a multimode plate reader (Infinite M Plex, Tecan). The GSH/GSSG ratio is then calculated from the resulting luminescence data.

Drug loading experiments: The drug loading experiments were done by measuring the optical density (OD) of the supernatants relative to concentration-standardized OD of doxorubicin (DOX, 44583, Sigma-Aldrich) loaded $TpAzo_{0.5}Bpy_{0.5}$ COF particles with a multimode plate reader (Infinite M Plex, Tecan) at 480 nm.[14] The particles (200 µg/mL) were dispersed in DOX solution with various concentrations (100-800 µg/mL), and these mixtures were stirred in the dark for 24 hours to allow the drug to be adsorbed. After 24 h, the suspension was centrifuged, and the supernatant was used to measure the drug loading. The maximum DOX loading capability for the particles was 81.24 ± 10.22 µg/200 µg $TpAzo_{0.5}Bpy_{0.5}$ COF particles. Therefore, 80 µg DOX and 200 µg $TpAzo_{0.5}Bpy_{0.5}$ COF were used for further experiments. After the drug loading procedure, drug release by time under various conditions, including various pH, H_2O_2, and glutathione concentrations, is also measured from the supernatant with a multimode plate reader as mentioned above.

Cell viability assays: The cell viability was measured using luciferase (CellTiter-Glo, Promega) and Calcein-AM and ethidium homodimer-1-based assays (Live/Dead, Thermo Fisher Scientific) after 24 hours of incubation at 37 °C and 5% CO_2. For the luciferase assay, an equal volume of the luciferase reagent to the treatment medium was added to each well at the end of incubation. The samples were put on an orbital shaker for two minutes to improve cell lysis, then incubated for 10 minutes at room temperature to stabilize luminescence signals. After incubation, the luminescence was measured with a multimode plate reader (Infinite M Plex, Tecan). The relative cell viability of each group was determined by comparing the luminescence signal levels of the treatment samples with the control samples. During the Live/Dead assay, Calcein-AM-stained live cells (green) and ethidium homodimer-1-stained dead cells (red) were imaged with a fluorescence microscope (BZ-X, Keyence).

Breast cancer organoid model: The breast cancer organoid model is produced by modification of a well-established cancer organoid model with SKBR3 human breast cancer cells and BJ dermal fibroblasts.[15] 5000 cells from each cell line were mixed and seeded into the ultra-low attachment round-bottom 96-well plates (Corning) for 4 days of incubation. After 4 days of incubation,

organoids are carried to Engelbreth-Holm-Swarm mouse sarcoma extracellular matrix (Matrigel, Corning) coated glass-bottom 35 mm culture dishes (Nunc, Thermo Scientific) and incubated for 24 hours before various treatments. After various treatments with COF particles, which are mentioned above, organoids are stained with Calcein-based Live/Dead assay or immunofluorescence markers in a similar fashion to 2D cell culture experiments.

Immunofluorescence Staining: The organoids were fixed with 4% paraformaldehyde and permeabilized with phosphate-buffered saline with 0.1% Triton-X-100 after treatments. Then, organoids were blocked in a Superblock solution (37515, Thermo Scientific). Organoids were incubated with Alexa Fluor 488-tagged Ki67 antibody (ab197234, Abcam) as a proliferation marker and Alexa Fluor 555-tagged alpha-tubulin antibody (ab275113, Abcam) as a cytoskeleton marker for 90 minutes at 37 ^{o}C and washed three times with phosphate-buffered saline with 0.1% Tween-20. After antibody incubations, all samples were mounted with a DAPI-supplemented mounting medium (ab104139, Abcam) to observe nuclei. Finally, immunofluorescence imaging was done using the Leica TCS SP8 DMi8 confocal laser scanning microscope (Leica, Wetzlar, Germany).

Statistical analysis: For cell culture experiments, the statistical comparisons between two groups were performed using t-tests, while one-way ANOVA was employed to analyze differences across multiple groups. For experiments involving multiple groups measured across different time points, two-way ANOVA was conducted using GraphPad Prism 8 software. "n" indicates the number of samples for each group, and "p" indicates statistical significance. Results were considered statistically significant when $p < 0.01$. All data are presented as mean ± SD, and experiments were performed at least three times to ensure reproducibility.

Synthesis procedures

COFs synthesis – Method 1: In general, pyrrolidine was used as a catalyst to obtain highly crystalline COFs.[16] In a typical $TpAzo_{1-x}Bpy_x$ COF synthesis, 0.38 mmol of amines (Azo and Bpy combination according to the mole fraction x. For instance, 40.5 mg of Azo and 35.6 mg of Bpy for $TpAzo_{0.5}Bpy_{0.5}$ COF) were dissolved within a 6 mL Biotage high-precision glass vial in 3 mL of anhydrous 1,4-dioxane. Subsequently, 0.24 mmol of Tp (50.3 mg) was added to the vial and sonicated for 10 minutes. Then, a magnetic stir bar was incorporated into the vial, 60 µL of pyrrolidine was added, and it was sealed with a septum cap. The reaction was kept at 120 °C on a

hot plate with stirring (300 rpm) for 3 days. Upon completion of the reaction period, the vial was allowed to cool naturally to room temperature and then unsealed. The resulting COF powder was filtered and rinsed with THF. To eliminate any residual linkers and catalysts, the COF powder was subjected to 24 hours of Soxhlet extraction in THF. Finally, the solvent was exchanged with methanol, and the powder was dried using supercritical CO_2.

COFs synthesis – Method 2: Method 2 was adapted from Method 1 but differed in the sequence of reactant addition.[16] In this case, 0.38 mmol of amines and 0.24 mmol of Tp were directly combined in the glass vial. Next, the linkers were dissolved in 3 mL of anhydrous 1,4-dioxane, and 60 µL of pyrrolidine were added. The vial was sealed with a septum cap and subjected to 10 minutes of sonication to achieve a homogenous dispersion. The reaction was kept at 120 °C on a hot plate with stirring (300 rpm) for 3 days. The workup was done following the same protocol as Method 1.

COFs palladation: $TpAzo_{1-x}Bpy_x$-CPd COF synthesis was based on the impregnation method. Typically, 30 mg of $TpAzo_{1-x}Bpy_x$ COFs was dispersed in 5 mL of methanol by 10 minutes of sonication. Then, to this dispersion, a certain amount of a 2 mg mL^{-1} $PdCl_2(CH_3CN)_2$ solution in methanol was added dropwise for 10 minutes. The amount of palladium was calculated according to the stoichiometry ratio 1 to 1 with azo units in $TpAzo_{1-x}Bpy_x$ COFs. For instance, to $TpAzo_{0.5}Bpy_{0.5}$ COF 6.4 mL of 2 mg mL^{-1} $PdCl_2(CH_3CN)_2$ solutions were added. Subsequently, the COF dispersion was stirred for 18 h. Upon completion of the reaction time, the resulting powder was filtered, thoroughly washed with a substantial volume of methanol, and air-dried overnight.

Supporting Figures

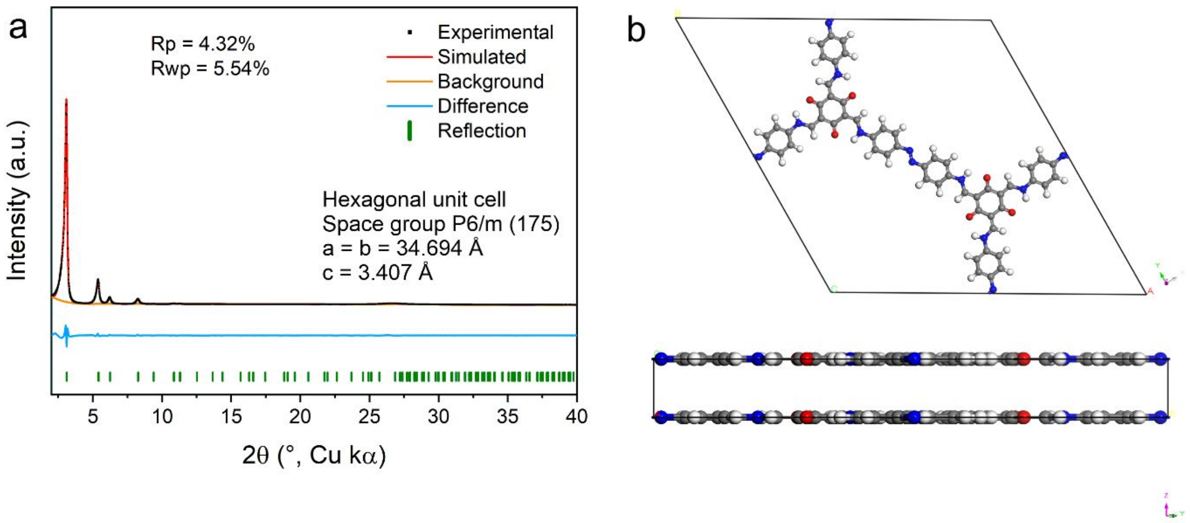

Figure S1. a) Refinement of TpAzo COF. b) Simulated TpAzo structure with AA stacking mode.

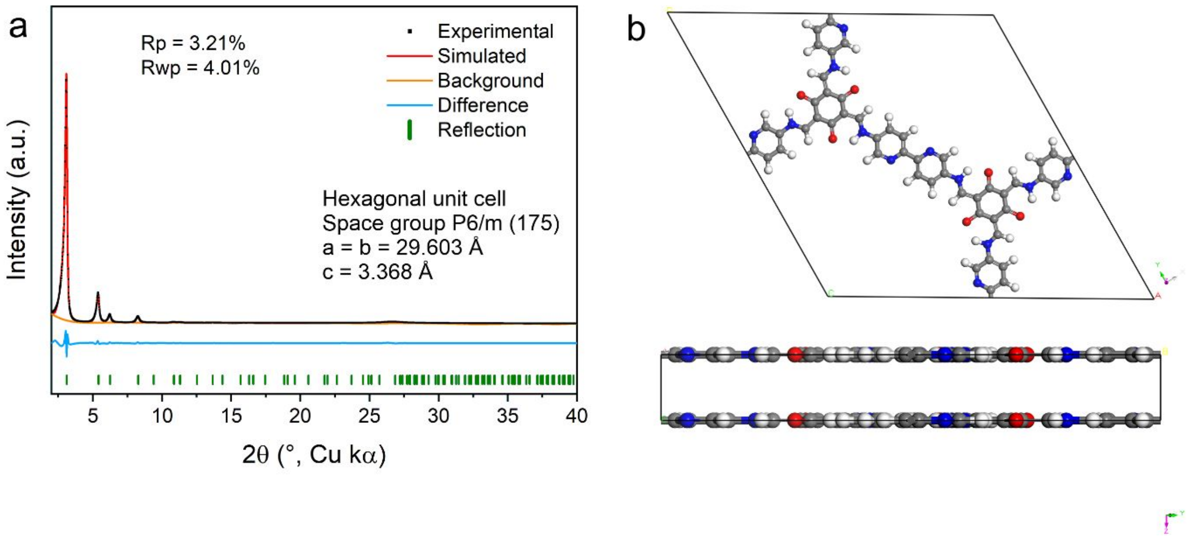

Figure S2. a) Refinement of TpBpy COF. b) Simulated TpBpy structure with AA stacking mode.

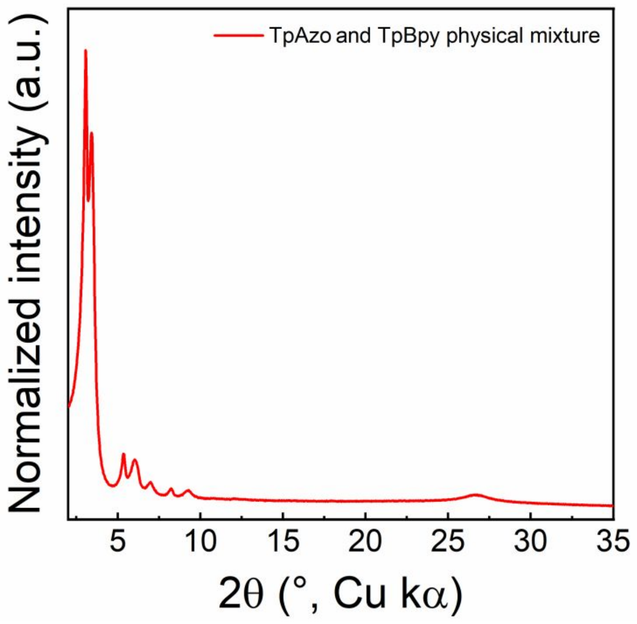

Figure S3. PXRD patterns of the physical mixture of TpAzo and TpBpy COFs.

Calculations of unit cell parameters in a hexagonal lattice:

Assuming a hexagonal crystal system for all COFs, the lattice parameters can be calculated using Bragg´s equation:

$$\frac{1}{d_{hkl}^2} = \frac{4}{3}\left(\frac{h^2+hk+k^2}{a^2}\right) + \left(\frac{l^2}{c^2}\right) \qquad \textbf{Equation 1}$$

Where d_{hkl} is the lattice distance between (hkl) planes, hkl corresponds to the Miller indices of a plane, a is the lattice parameter in the h and k direction, and c in the l direction.[17]

For the plane (100); $h = 1$, $k = 0$, $l = 0$.

$$a = \frac{2\sqrt{3}d}{3} \qquad \textbf{Equation 2}$$

For the plane (001); $h = 0$, $k = 0$, $l = 1$.

$$c = d \qquad \textbf{Equation 3}$$

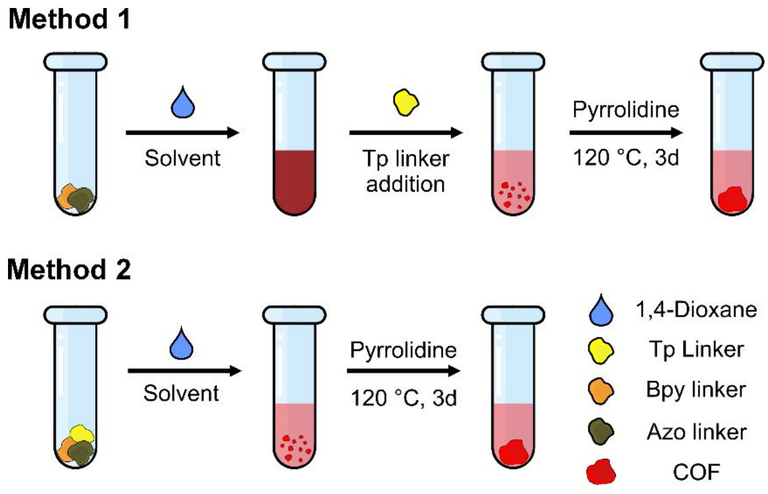

Figure S4. Schematic of the synthetic methods used in the synthesis of $TpAzo_{1-x}Bpy_x$ COFs.

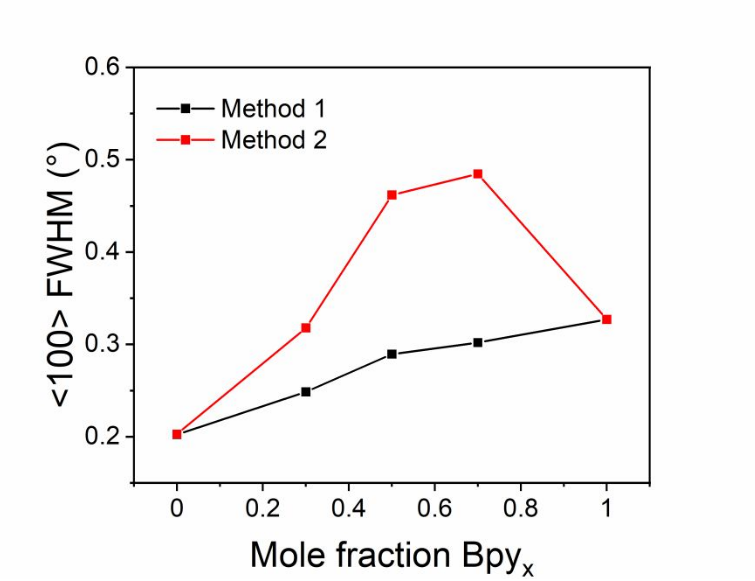

Figure S5. The full width at half maximum (FWHM) of the 100 reflection, as a descriptor of the crystallinity of $TpAzo_{1-x}Bpy_x$ COFs synthesized by two different methods.

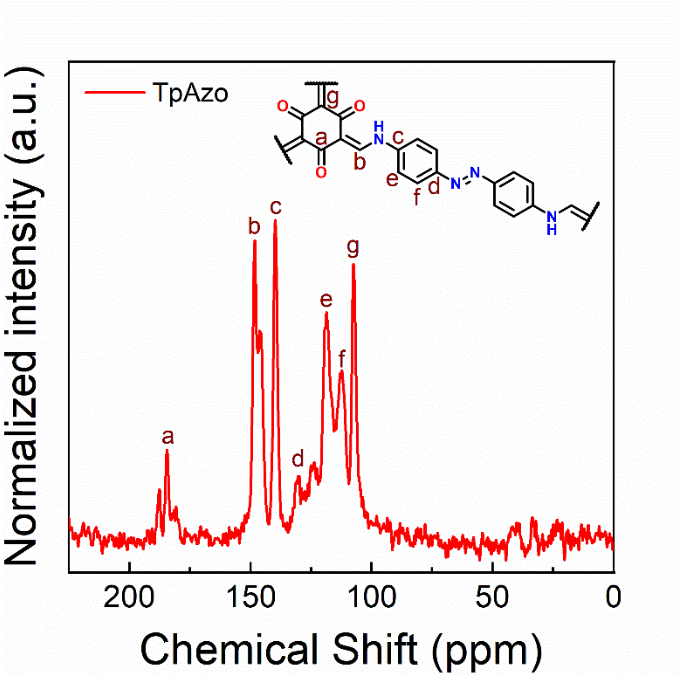

Figure S6. ^{13}C-ssNMR spectrum of TpAzo and the assignment of the carbon signals.

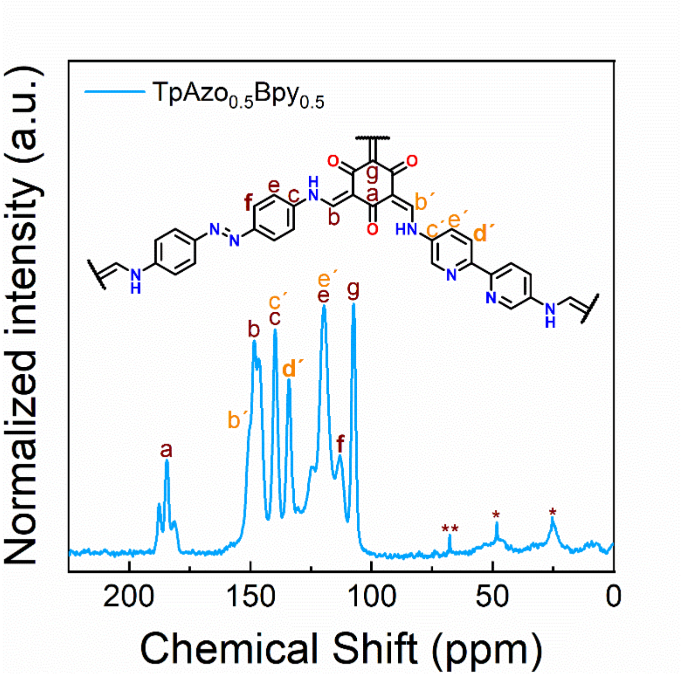

Figure S7. ^{13}C-ssNMR spectrum of $TpAzo_{0.5}Bpy_{0.5}$ and the assignment of the carbon signals. The single asterisks indicate signals from pyrrolidine (catalyst) and double asterisks indicate signals from 1,4-dioxane (solvent).

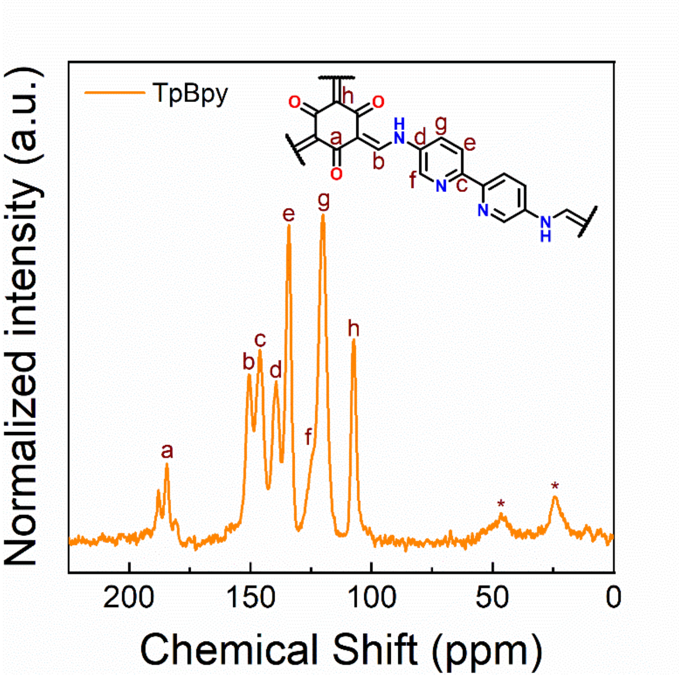

Figure S8. ^{13}C-ssNMR spectrum of TpBpy and the assignment of the carbon signals. The asterisks indicate signals from pyrrolidine (catalyst).

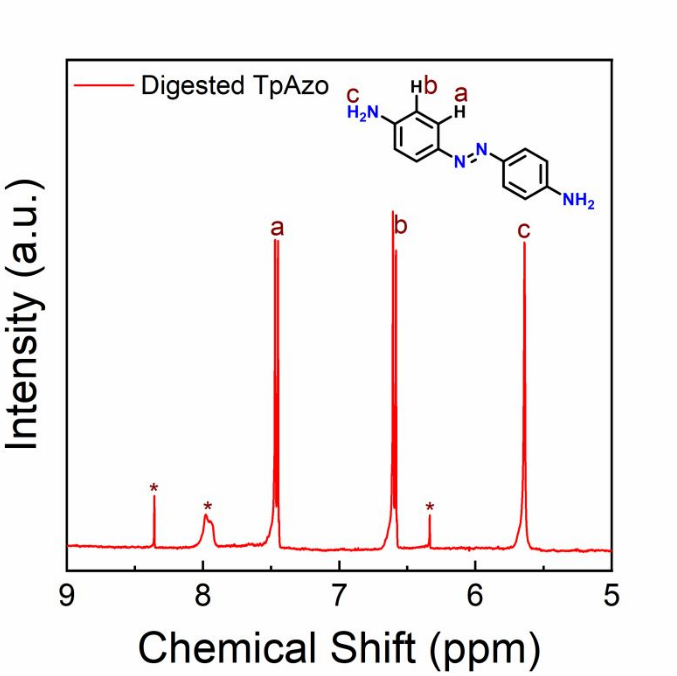

Figure S9. ^{1}H-NMR spectrum of digested TpAzo and the assignment of the protons. The asterisks indicate residual signals from Tp-MeNH$_2$.

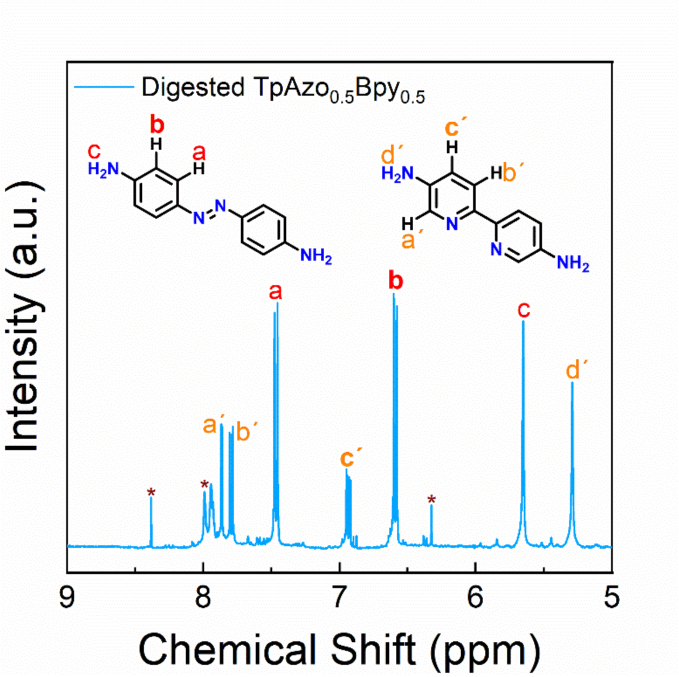

Figure S10. ^{1}H-NMR spectrum of digested $TpAzo_{0.5}Bpy_{0.5}$ and the assignment of the protons. The asterisks indicate residual signals from Tp-$MeNH_2$.

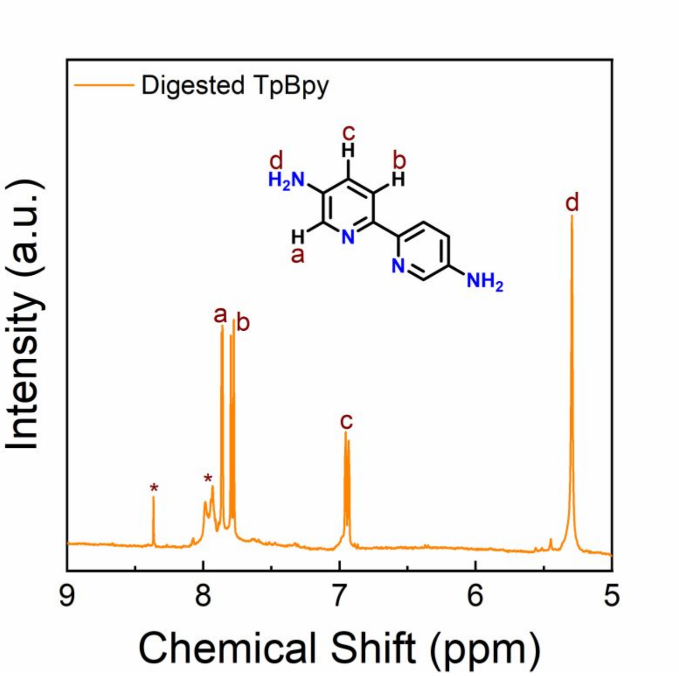

Figure S11. The ^{1}H-NMR spectrum of digested TpBpy and the assignment of the protons. The asterisks indicate residual signals from Tp-$MeNH_2$.

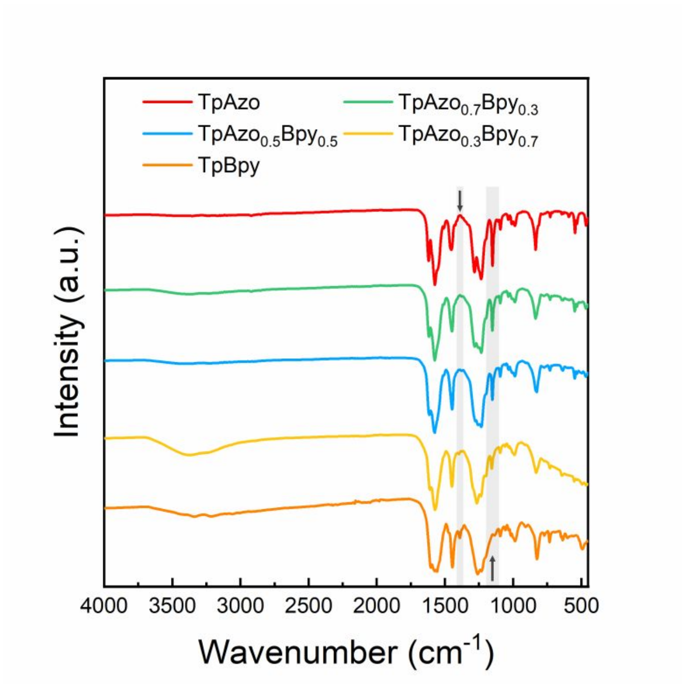

Figure S12. FTIR spectra of TpAzo$_{1\text{-}x}$Bpy$_x$ COFs.

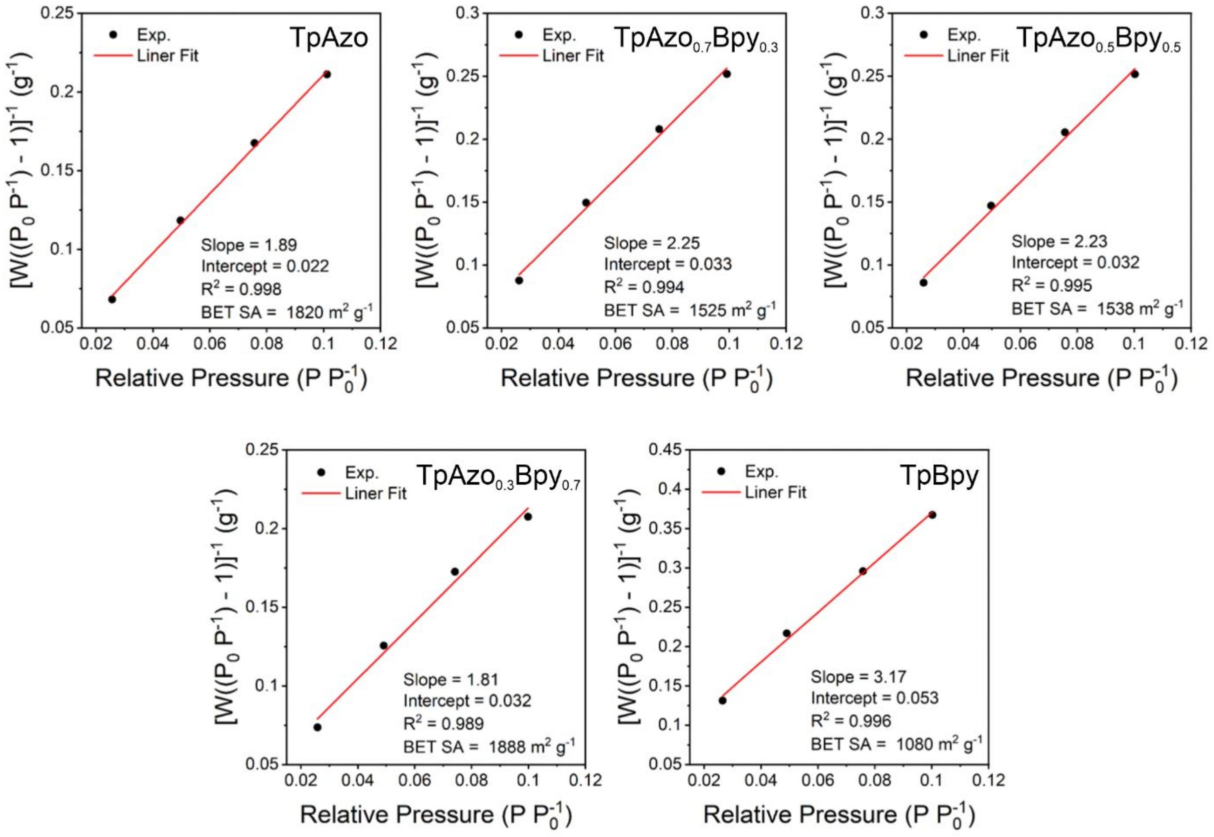

Figure S13. BET plots of $TpAzo_{1-x}Bpy_x$ COFs and their linear fitting.

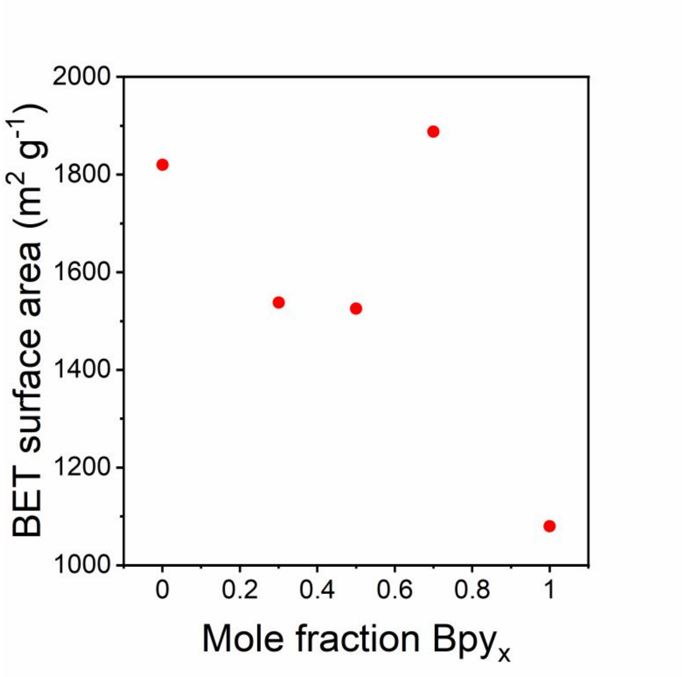

Figure S14. BET surface areas of $TpAzo_{1-x}Bpy_x$ COFs. No correlation is observed between the Bpy mole fraction and the BET surface area of the resulting COFs.

Table S1. Pd content of the COFs determined by ICP-OES.

COF	Pd content (wt%)	
**TpAzo-CPd_1**	12.5	12.2 ± 0.3
**TpAzo-CPd_2**	11.9	
**$TpAzo_{0.7}Bpy_{0.3}$_1**	16.0	15.9 ± 0.1
**$TpAzo_{0.7}Bpy_{0.3}$_2**	15.7	
**$TpAzo_{0.5}Bpy_{0.5}$_1**	14.4	14.5 ± 0.1
**$TpAzo_{0.5}Bpy_{0.5}$_2**	14.6	
**$TpAzo_{0.3}Bpy_{0.7}$_1**	9.3	9.4 ± 0.1
**$TpAzo_{0.3}Bpy_{0.7}$_2**	9.4	
**TpBpy-Pd_1**	17.5	18.4 ± 1.0
**TpBpy-Pd_2**	19.4	
**$TpAzo_{0.5}Bz_{0.5}$_1**	13.4	13.5 ± 0.1
**$TpAzo_{0.5}Bz_{0.5}$_2**	13.7	

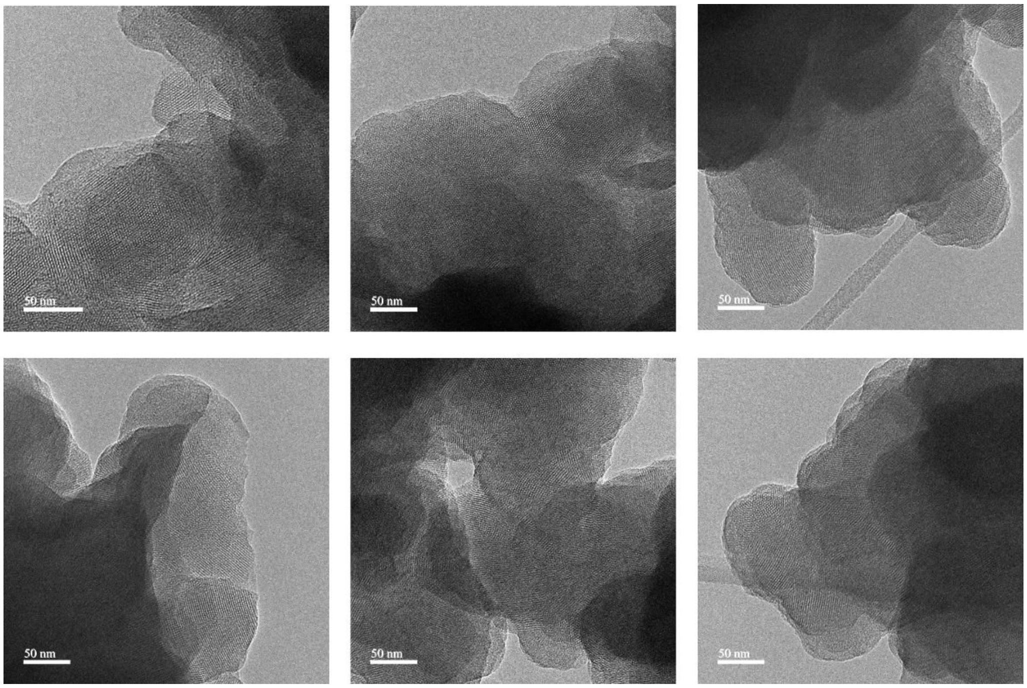

Figure S15. TEM images of $TpAzo_{0.5}Bpy_{0.5}$-CPd show the preserved crystallinity of the COF and the absence of dark spots characteristic of Pd NPs.

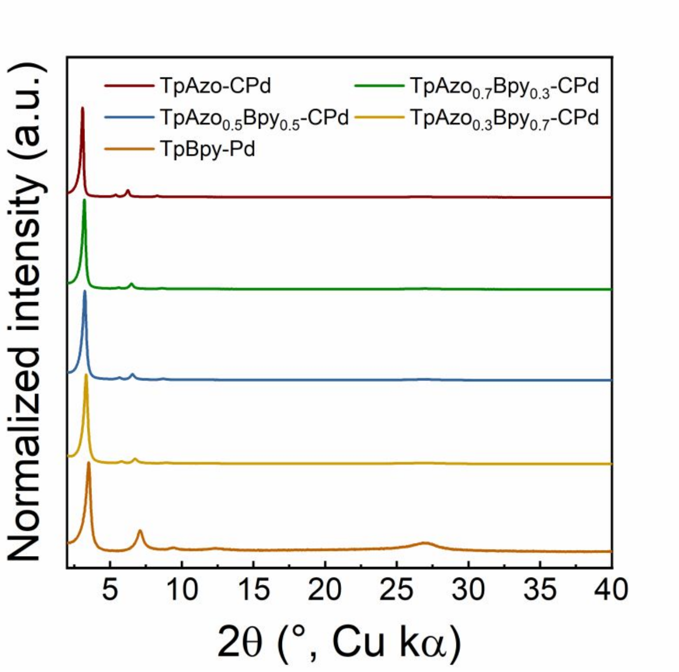

Figure S16. PXRD patterns of the $TpAzo_{1-x}Bpy_x$-CPd COFs (*i.e.* palladated $TpAzo_{1-x}Bpy_x$ COFs).

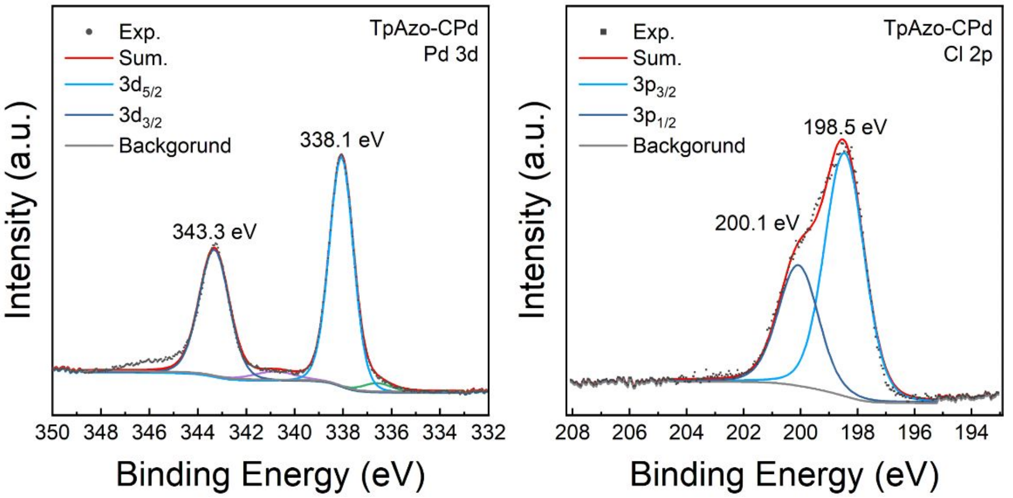

Figure S17. Pd (left) and Cl (right) XPS spectra of TpAzo-CPd COF.

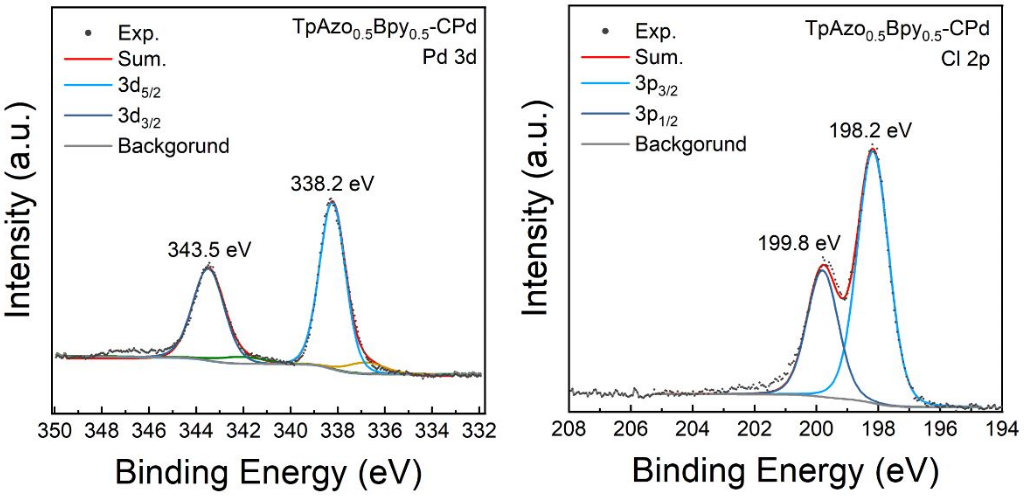

Figure S18. Pd (left) and Cl (right) XPS spectra of the $TpAzo_{0.5}Bpy_{0.5}$-CPd COF (*i.e.* palladated $TpAzo_{0.5}Bpy_{0.5}$ COF).

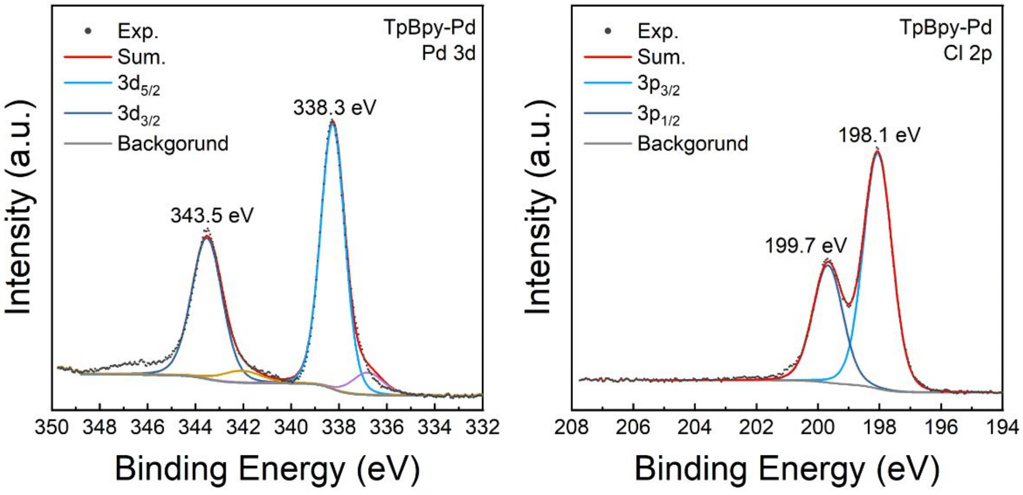

Figure S19. Pd (left) and Cl (right) XPS spectra of the TpBpy-Pd COF (*i.e.* palladated TpBpy COF).

Table S2. The calculated atomic composition of palladated COFs from XPS spectra.

COF	Cl atomic%	Pd atomic%	Atomic ration Cl/Pd
**TpAzo-CPd**	3.6	2.3	1.6
**TpAzo$_{0.5}$Bpy$_{0.5}$-CPd**	3.4	2.0	1.7
**TpBpy-Pd**	6.5	3.6	1.8

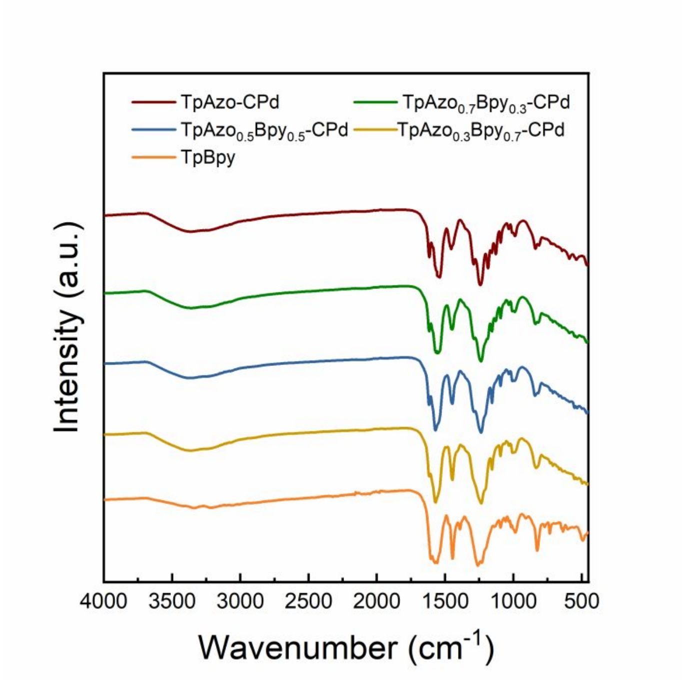

Figure S20. FTIR spectra of $TpAzo_{1-x}Bpy_x$-CPd COFs.

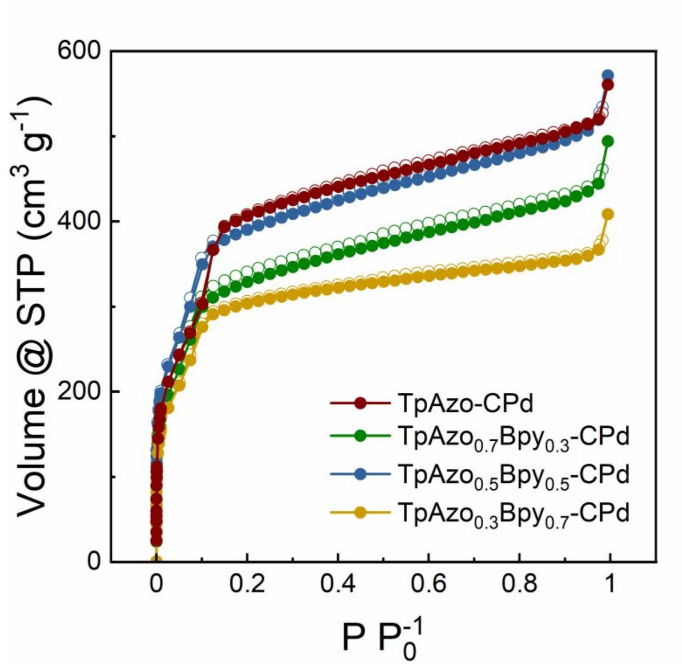

Figure S21. N_2 sorption isotherms of $TpAzo_{1-x}Bpy_x$-CPd COFs (solid dots represent the adsorption and empty dots represent the desorption).

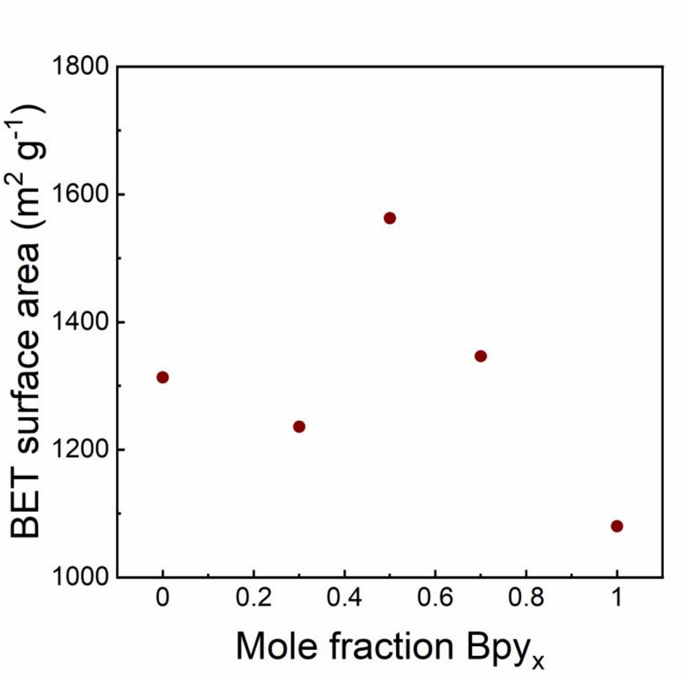

Figure S22. BET surface areas of $TpAzo_{1-x}Bpy_x$-CPd COFs. No correlation is observed between the Bpy mole fraction and the BET surface area of the resulting COF.

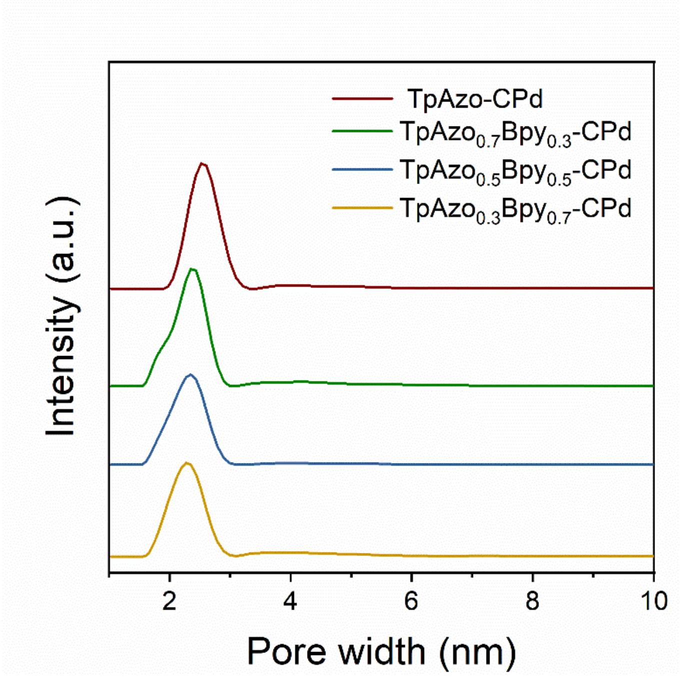

Figure S23. Pore size distributions of $TpAzo_{1-x}Bpy_x$-CPd COFs.

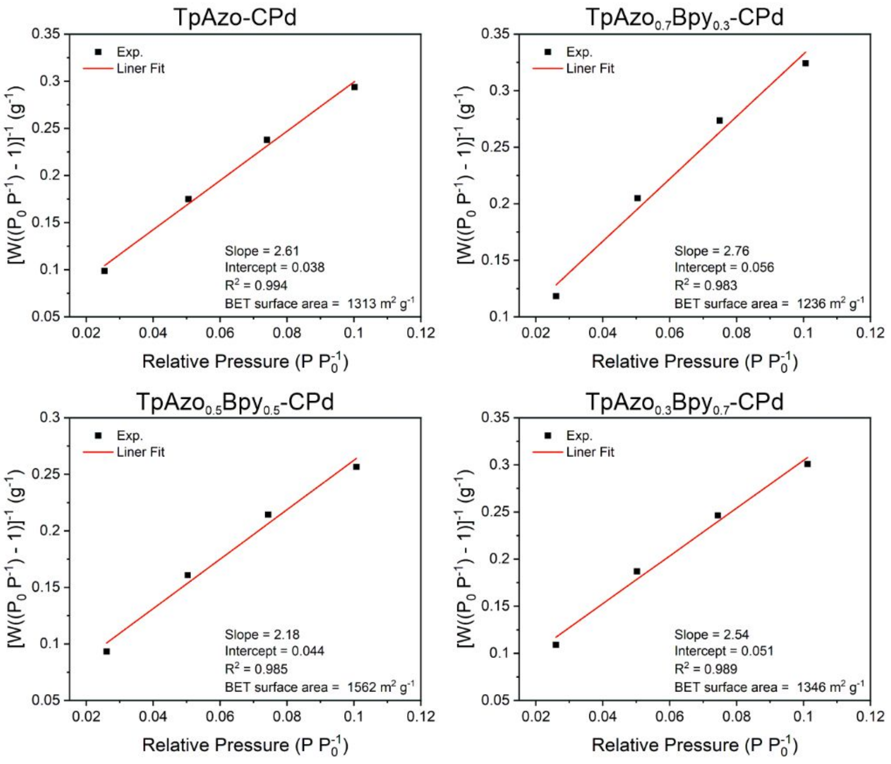

Figure S24. BET plots of $TpAzo_{1-x}Bpy_x$-CPd COFs and their linear fitting.

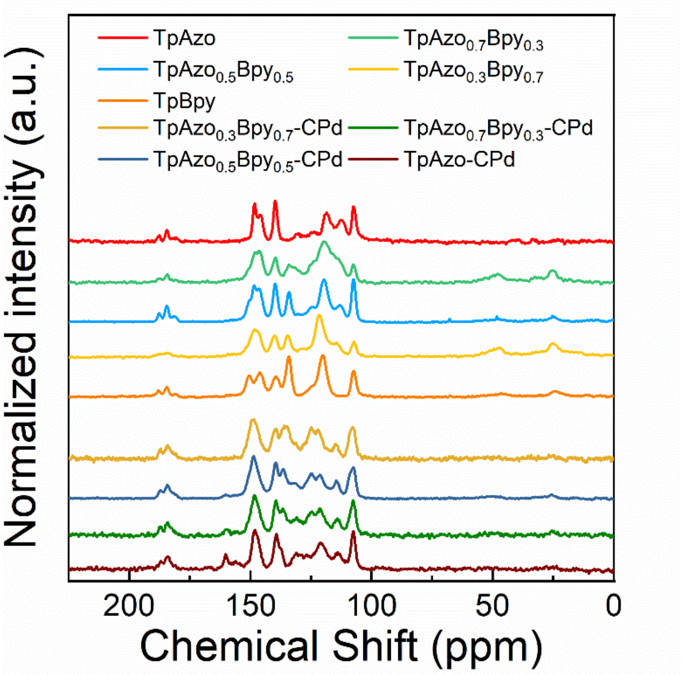

Figure S25. ^{13}C-ssNMR spectra of pristine $TpAzo_{1-x}Bpy_x$ and $TpAzo_{1-x}Bpy_x$-CPd COFs.

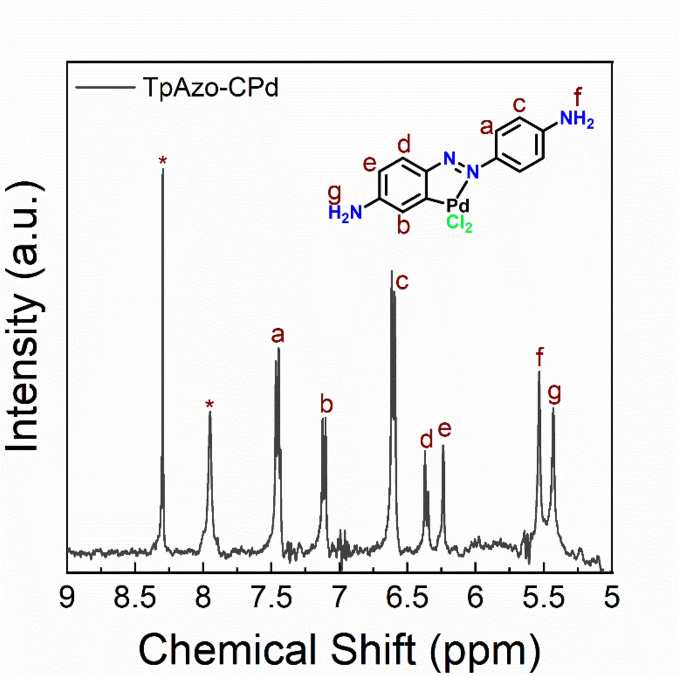

Figure S26. ^{1}H-NMR spectrum of digested TpAzo-CPd and its signals assignment. The asterisks indicate residual signals from Tp-MeNH$_2$.

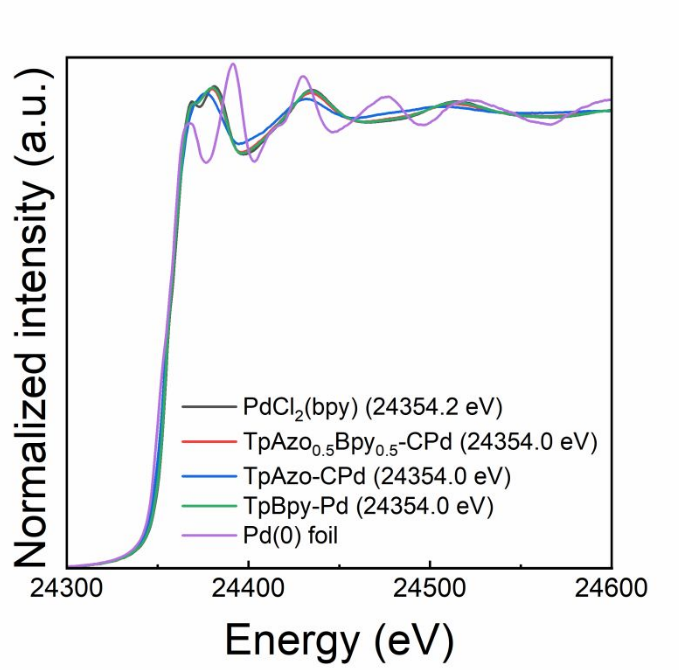

Figure S27. XANES spectra (including the beginning of the EXAFS part) of PdCl$_2$(bpy) (black), TpAzo$_{0.5}$Bpy$_{0.5}$-CPd (red), TpAzo-CPd (blue), TpBpy-Pd (green) and a of Pd(0) foil (purple) used for energy calibration.

Table S3: Edge energies of $PdCl_2$(bpy), $TpAzo_{0.5}Bpy_{0.5}$-CPd, TpAzo-CPd, TpBpy-Pd, and of Pd(0) foil used as reference.

**Sample**	**Edge energy E_0 (eV)**
$PdCl_2$(bpy)	24354.2
$TpAzo_{0.5}Bpy_{0.5}$-CPd	24354.0
TpAzo-CPd	24354.0
TpBpy-Pd	24354.0
Pd(0) foil	24350.0

EXAFS Analysis

The *k*- and *R*-ranges of the EXAFS-analysis of compounds $PdCl_2$(bpy), $TpAzo_{0.5}Bpy_{0.5}$-CPd, TpAzo-CPd, TpBpy-Pd together with the respective values for *R*-factor, reduced Chi-square and S_0^2-parameter are collected in **Table S4**. **Figure S28** compares the Fourier-transformed EXAFS-spectra of $PdCl_2$(bpy), $TpAzo_{0.5}Bpy_{0.5}$-CPd, TpAzo-CPd, and TpBpy-Pd, whereas **Figure S5** illustrates the fitted function, experimental data, residual plot as well as first shell contributions for these compounds in the *R*- and *k*-space. **Table S5** shows coordination numbers (*N*), bond lengths ($R + \Delta R$), and Debye-Waller factors (σ^2) for all scattering paths of $PdCl_2$(bpy), $TpAzo_{0.5}Bpy_{0.5}$-CPd, TpAzo-CPd, TpBpy-Pd obtained by EXAFS fitting with the Artemis program. For the TpAzo-CPd-sample, two possible bonding situations were evaluated: TpAzo-CPd (with 2xMeCN) describes the coordination of cyclometallated 4,4'-diaminoazobenzene together with two MeCN ligands, TpAzo-CPd (with 1xMeCN, 1xCl) the coordination of 4,4'-diaminoazobenzene together with one MeCN ligand and one Cl anion.

The EXAFS results for bond lengths and coordination number of $PdCl_2$(bpy) are in reasonable agreement with its crystallographic data (Table S4).[18]

Table S4: First shell coordination numbers (*N*), bond lengths ($R + \Delta R$) and Debye-Waller factors (σ^2) of TpBpy-Pd, TpAzo-CPd, $TpAzo_{0.5}Bpy_{0.5}$-CPd and $PdCl_2$(bpy) as well as comparison with crystallographic data.

Scattering path	*N*	$R + \Delta R$ [Å]	σ^2 [Å^2]	Crystallographic data[5] [Å]
**$PdCl_2$(bpy)**				
Pd-N	1.9(1)	2.025(9)	0.0022(6)	2.017
Pd-Cl	1.9(1)	2.289(6)	0.0026(3)	2.294
**TpBpy-Pd**				
Pd-N	1.7(1)	2.017(6)	0.0023(4)	-
Pd-Cl	2.1(1)	2.290(4)	0.0034(2)	-
**TpAzo-Pd**				
Pd-C	0.9(1)	1.941(12)	0.0026(6)	-
Pd-N^1	1.0(2)	2.028(14)	0.0021(6)	-
Pd-N^2	1.0(3)	2.188(33)	0.0022(6)	-
Pd-Cl	1.0(1)	2.289(17)	0.0038(7)	-
**$TpAzo_{0.5}Bpy_{0.5}$-CPd**				
Pd-N	1.5(1)	2.038(5)	0.0021(5)	-
Pd-Cl	1.8(1)	2.295(4)	0.0030(2)	-

Benchmarking the EXAFS analysis results of TbBpy-Pd against its homogeneous analog $PdCl_2$(bpy) (Table S4) reveals almost the same bond lengths and coordination number of the Pd(II) centers in both samples. This is in good agreement with the similar XANES spectra of both compounds (Figure 4e).

The chemical environment of the Pd(II) centers in TpAzo-CPd is best described by a fitting model assuming that the ligand sphere comprises a cyclopalladated diaminobenzene-moiety (Figure 1), along with an acetonitrile and a chlorido ligand. The acetonitrile unit derives from $PdCl_2(CH_3CN)_2$ used as a homogeneous precursor for the immobilization process. Alternatively, the substitution of both chlorido ligands in $PdCl_2(CH_3CN)_2$ with the cyclopalladated diaminobenzene-moiety would also have been feasible. However, EXAFS analysis based on this assumption results in an unreasonable coordination number of CN = 6 to 7 to compensate for the loss in signal intensity of the heavy chloride back scatterer as well as worse fit quality in terms of *R*-factor and reduced Chi-square parameter. For this reason, this coordination mode is excluded from further consideration. The coordination of acetonitrile can be in principle also exchanged by the coordination of MeOH or H_2O. This is the result of the very similar backscattering amplitudes of nitrogen and oxygen, which makes it difficult to distinguish between them by EXAFS.

Finally, EXAFS-analysis confirms that the bond lengths of the donating atoms in the first coordination shell of $TpAzo_{0.5}Bpy_{0.5}$-CPd and TpBpy-Pd are in reasonable agreement with each other. Moreover, multiplying the degeneracy of the Pd–Cl scattering path in TpBpy-Pd by the factor of 0.8; results approximately in the degeneracy of the corresponding scattering path in $TpAzo_{0.5}Bpy_{0.5}$-CPd (Table S5). According to the LC-XANES-Fit (Figure 4f), the factor of 0.8 reflects the proportion of the Pd(II)-coordination mode in TpBpy-Pd being present in $TpAzo_{0.5}Bpy_{0.5}$-CPd. Since the number of coordinating chloride anions in TpBpy-Pd is twice as high as in TpAzo-CPd (Table S4), the lower degeneracy of the Pd-Cl-scattering path in $TpAzo_{0.5}Bpy_{0.5}$-CPd compared to TpBpy-Pd is reasonable.

Therefore, EXAFS analysis data of $PdCl_2$(bpy), TpAzo-CPd, TpBpy-Pd and $TpAzo_{0.5}Bpy_{0.5}$-CPd support the corresponding XANES results and identify the bonding situation in the first coordination shell of $PdCl_2$(bpy) and TpBpy-Pd as the dominant coordination mode in $TpAzo_{0.5}Bpy_{0.5}$-CPd.

Table S5: *k*- and *R*-ranges as well as corresponding fit parameters of the analysis of compounds PdCl2(bpy), $TpAzo_{0.5}Bpy_{0.5}$-CPd, TpAzo-CPd, TpBpy-Pd. For the TpAzo-CPd-sample, two possible bonding situations were evaluated: TpAzo-CPd (with 2xMeCN) describes the coordination of cyclometallated 4,4'-diaminoazobenzene together with two MeCN ligands,

TpAzo-CPd (with 1xMeCN, 1xCl) the coordination of 4,4'-diaminoazobenzene together with one MeCN ligand and one Cl anion.

sample	*k*-range [$Å^{-1}$]	*R*-range [Å]	*R*-factor	reduced Chi-square	S_0^2-value
$PdCl_2$(bpy)	4.20 – 15.40	1.07 – 15.40	0.0044	135	1.0
$TpAzo_{0.5}Bpy_{0.5}$-CPd	4.15 – 15.30	1.10 – 4.00	0.0068	26	1.0
TpAzo-CPd (with 2x Cl)	4.15 – 15.00	1.10 – 4.00	0.0076	18	1.0
TpAzo-CPd (w/o Cl)	4.15 – 15.00	1.05 – 4.00	0.0156	35	1.0
TpBpy-Pd	4.15 – 15.30	1.05 – 4.00	0.0051	53	1.0

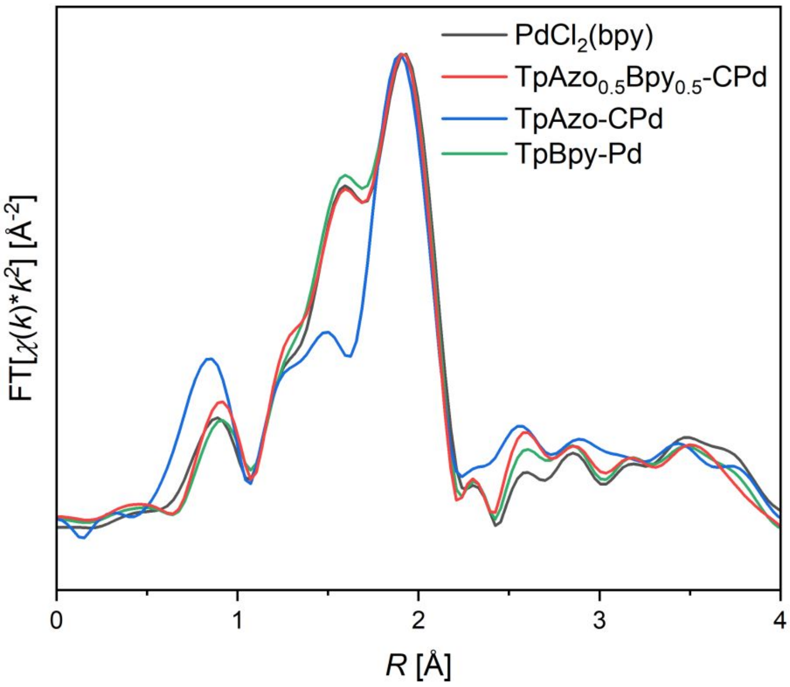

Figure S28. Comparison of the Fourier-transformed EXAFS-spectra of $PdCl_2$(bpy) (black), $TpAzo_{0.5}Bpy_{0.5}$-CPd (red), TpAzo-CPd (blue) and TpBpy-Pd (green).

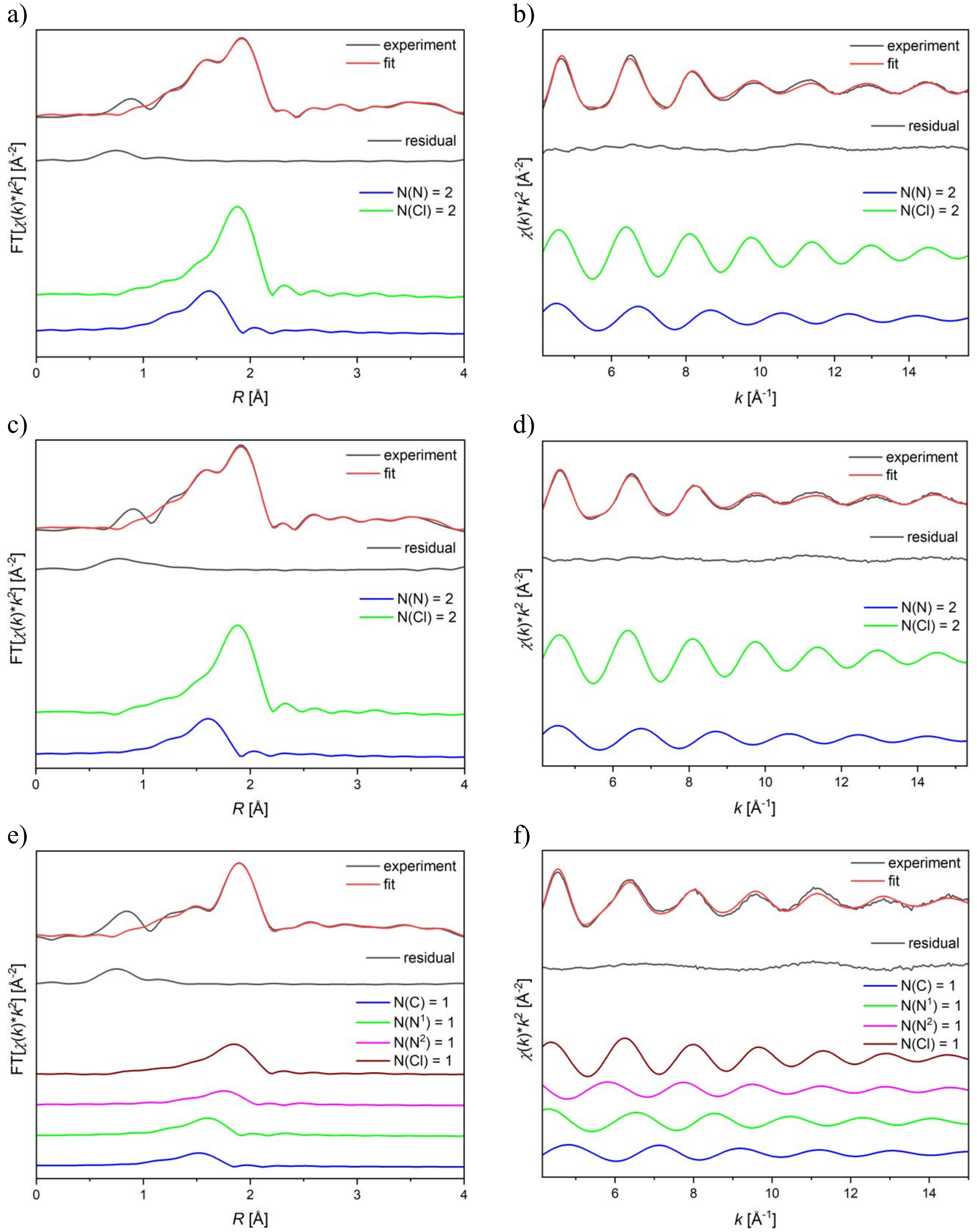
a)
experiment
fit
residual
N(N) = 2
N(Cl) = 2
FT[χ(k)*k²] [Å⁻²]
R [Å]
b)
experiment
fit
residual
N(N) = 2
N(Cl) = 2
χ(k)*k² [Å⁻²]
k [Å⁻¹]
c)
experiment
fit
residual
N(N) = 2
N(Cl) = 2
FT[χ(k)*k²] [Å⁻²]
R [Å]
d)
experiment
fit
residual
N(N) = 2
N(Cl) = 2
χ(k)*k² [Å⁻²]
k [Å⁻¹]
e)
experiment
fit
residual
N(C) = 1
N(N¹) = 1
N(N²) = 1
N(Cl) = 1
FT[χ(k)*k²] [Å⁻²]
R [Å]
f)
experiment
fit
residual
N(C) = 1
N(N¹) = 1
N(N²) = 1
N(Cl) = 1
χ(k)*k² [Å⁻²]
k [Å⁻¹]

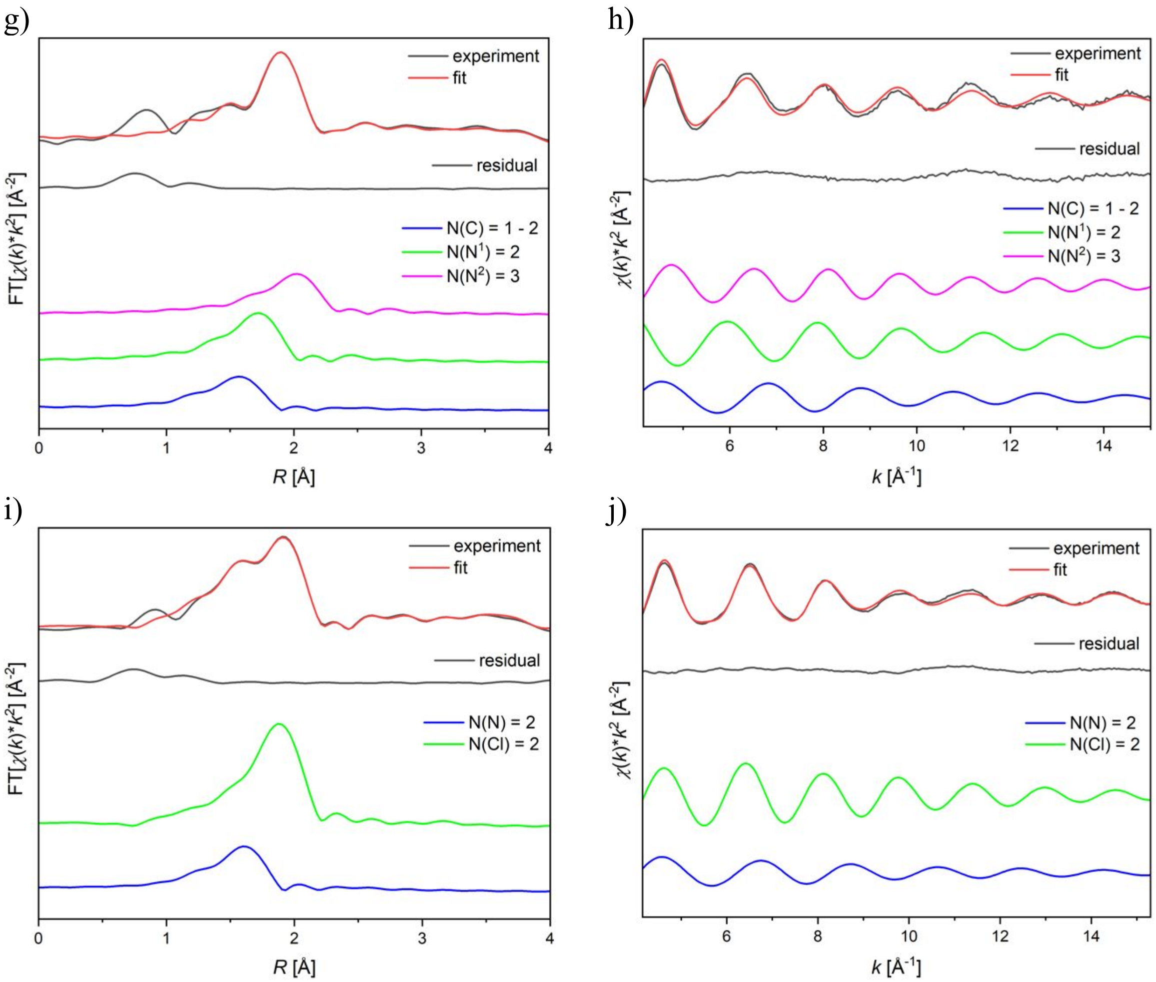

Figure S29: Fitted function compared with experimental data, residual plot, and first coordination shell paths for $PdCl_2$(bpy), $TpAzo_{0.5}Bpy_{0.5}$-CPd, TpAzo-CPd, TpBpy-Pd in the *k*- and *R*-space: a) *R*-space of $PdCl_2$(bpy), b) *k*-space of $PdCl_2$(bpy), c) *R*-space of $TpAzo_{0.5}Bpy_{0.5}$-CPd, d) *k*-space of $TpAzo_{0.5}Bpy_{0.5}$-CPd, e) *R*-space of TpAzo-CPd (with 1xMeCN, 1xCl), f) *k*-space of TpAzo-CPd (with 1xMeCN, 1xCl), g) *R*-space of TpAzo-CPd (with 2xMeCN), h) *k*-space of TpAzo-CPd (with 2xMeCN), i) *R*-space of TpBpy-Pd, j) *k*-space of TpBpy-Pd. For the TpAzo-CPd-sample, two possible bonding situations were evaluated: TpAzo-CPd (with 2xMeCN) describes the coordination of cyclometallated 4,4'-diaminoazobenzene together with two MeCN ligands, TpAzo-CPd (with 1xMeCN, 1xCl) the coordination of 4,4'-diaminoazobenzene together with one MeCN ligand and one Cl anion.

Table S6: Coordination numbers (*N*), bond lengths ($R + \Delta R$) and Debye-Waller factors (σ^2) for all scattering paths of $PdCl_2$(bpy), $TpAzo_{0.5}Bpy_{0.5}$-CPd, TpAzo-CPd, TpBpy-Pd obtained by EXAFS fitting with *the* Artemis program. Mo–CC-, Mo–NC-, Mo-CCC- and Mo-NCN scattering paths represent multiple scattering paths. For the TpAzo-CPd-sample, two possible bonding situations were evaluated: TpAzo-CPd (with 2xMeCN) describes the coordination of cyclometallated 4,4'-diaminoazobenzene together with two MeCN ligands**,** TpAzo-CPd (with 1xMeCN, 1xCl) the coordination of 4,4'-diaminoazobenzene together with one MeCN ligand and one Cl anion.

**scattering paths**	**Pd(Cl$_2$)(bpy)**			**TpBpy-Pd**		
	N	$R + \Delta R$ [Å]	σ^2 [Å^2]	*N*	$R + \Delta R$ [Å]	σ^2 [Å^2]
Pd–N	1.9(1)	2.025(9)	0.0022(6)	1.7(1)	2.017(6)	0.0023(4)
Pd–Cl	1.9(1)	2.289(6)	0.0026(3)	2.1(1)	2.290(4)	0.0034(2)
Pd–C	5.7(8)	2.873(25)	0.0075(24)	4.3(8)	2.978(18)	0.0060(21)
Pd–NC	21.2(42)	3.135(38)	0.0037(25)	22.9(62)	3.007(27)	0.0052(24)
Pd–NC	20.8(24)	2.963(25)	0.0041(29)	13.3(40)	3.201(29)	0.0053(25)
Pd–CC	18.5(33)	4.466(63)	0.0089(66)	21.7(45)	4.431(23)	0.0100(34)
Pd–CCC	14.3(55)	4.193(70)	0.0113 (80)			
Pd–NC	16.0(57)	4.321(125)	0.0075(30)	23.4(35)	4.274(21)	0.0066(19)

	**TpAzo-CPd (mit 1xMeCN, 1xCl)**		
	N	$R + \Delta R$ [Å]	σ^2 [Å^2]
Pd–C	0.9(1)	1.941(12)	0.0026(6)
Pd–N	1.0(2)	2.028(14)	0.0021(6)
Pd–N	1.0(3)	2.188(33)	0.0022(6)
Pd–Cl	1.0(1)	2.289(17)	0.0038(7)
Pd–C	3.7(6)	2.929(17)	0.0037(9)
Pd–C	2.2(8)	3.374(28)	0.0040(9)
Pd–NN	32.2(50)	2.933(16)	0.0037(10)
Pd–NC	17.4(40)	3.125(27)	0.0042(10)
Pd–CC	23.4(45)	4.170(28)	0.0128(32)
Pd–NC	32.9(59)	4.338(27)	0.0053(13)

	**TpAzo-CPd (mit 2xMeCN)**		
	N	$R + \Delta R$ [Å]	σ^2 [Å^2]
Pd–C	1.6(2)	2.003(15)	0.0029(10)
Pd–N	2.2(2)	2.158(12)	0.0023(5)
Pd–N	2.8(2)	2.481(24)	0.0025(6)
Pd–NC	4.4(7)	2.817(46)	0.0032(15)
Pd–NCN	5.5(9)	2.899(49)	0.0062(17)
Pd–NC	14.3(40)	3.097(83)	0.0046(12)
Pd–NC	14.0(95)	3.521(99)	0.0075(21)
Pd–CC	10.6(48)	4.147(74)	0.0104(52)

Pd–NC	24.8(76)	4.306(66)	0.0055(14)

	**$TpAzo_{0.5}Bpy_{0.5}$-CPd**		
	N	$R + \Delta R$ [Å]	σ^2 [Å^2]
Pd–N	1.5(1)	2.038(8)	0.0021(5)
Pd–Cl	1.8(1)	2.295(5)	0.0030(2)
Pd–C	4.0(10)	2.881(27)	0.0069(36)
Pd–NC	23.3(58)	3.140(27)	0.0044(26)
Pd–NC	16.1(45)	3.184(33)	0.0057(24)
Pd–CC	18.0(51)	4.244(41)	0.0100(59)
Pd–NC	23.8(42)	4.368(30)	0.0073(31)

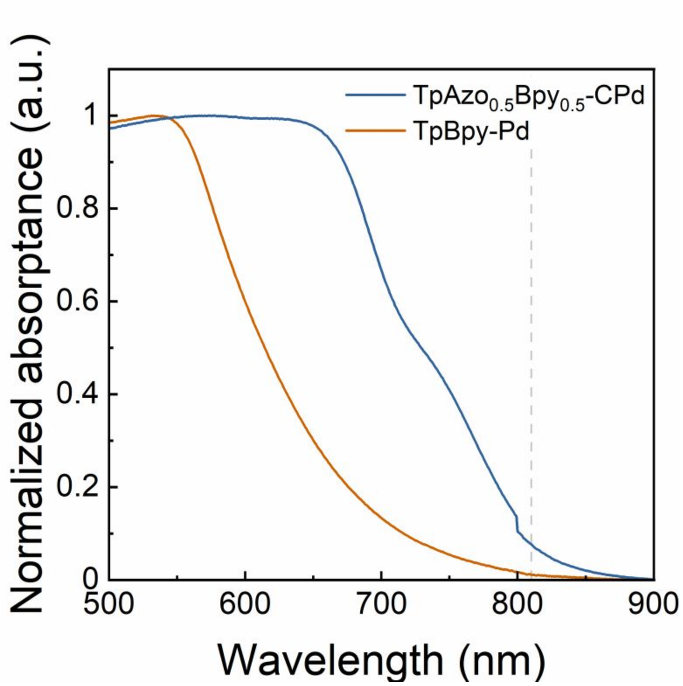

Figure S30. Vis-NIR spectra of $TpAzo_{0.5}Bpy_{0.5}$-CPd and TpBpy-Pd COFs.

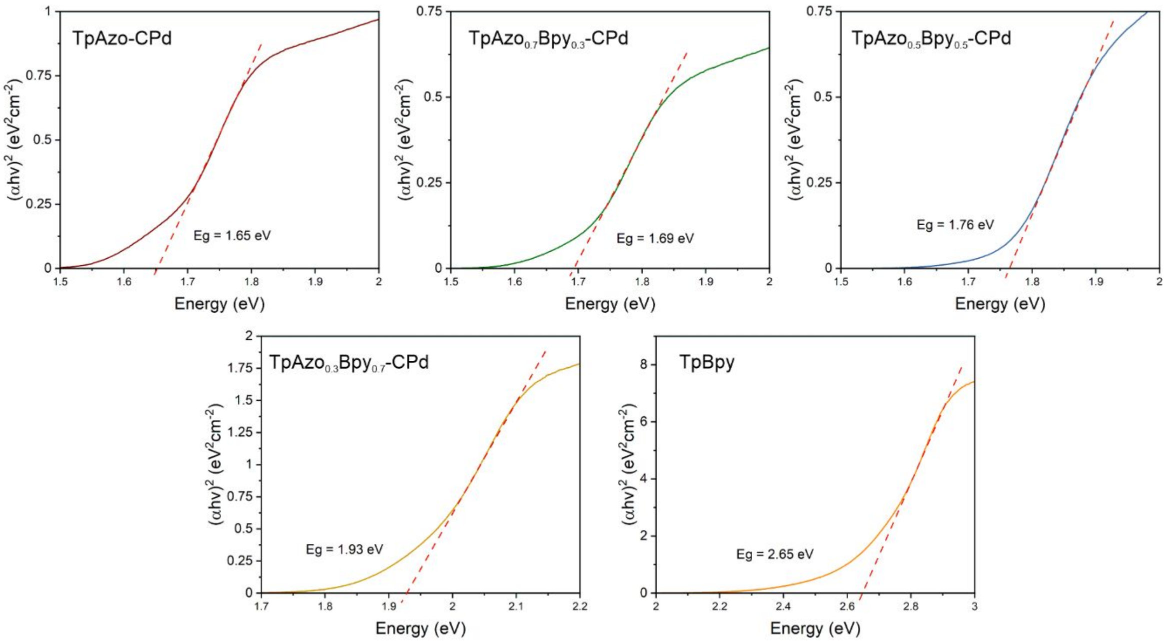

Figure S31. Tauc plot for the determination of the optical band gap of the $TpAzo_{1-x}Bpy_x$-CPd COFs.

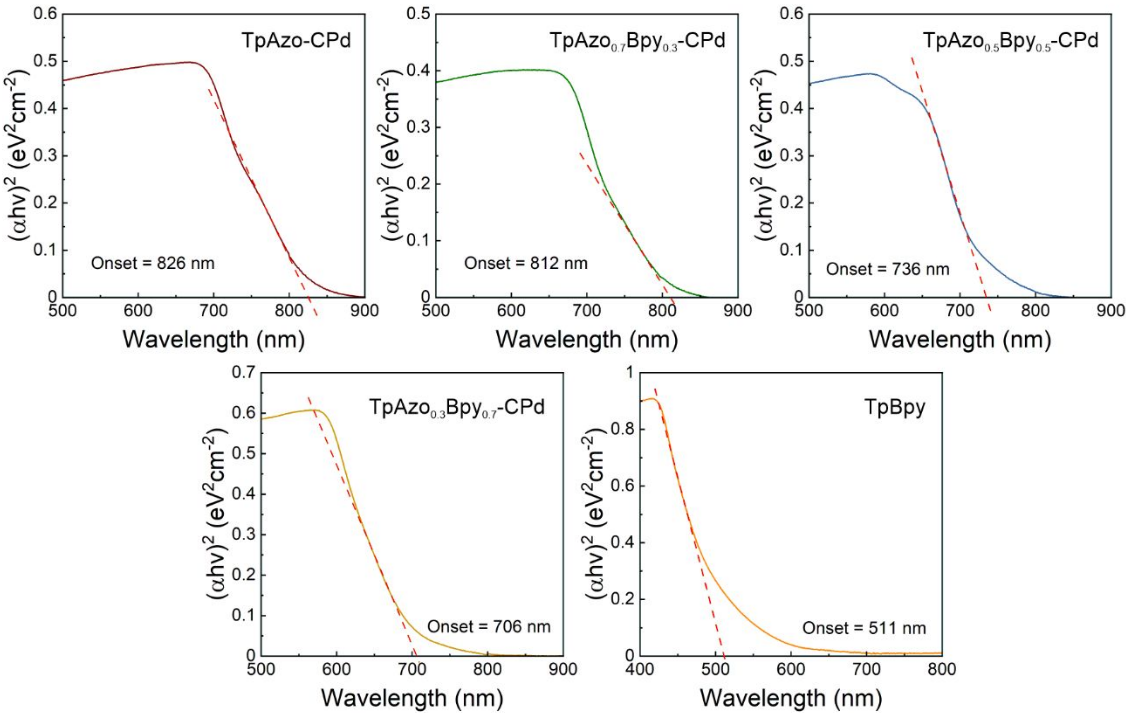

Figure S32. Vis-NIR spectra for the determination of the absorption onset of the $TpAzo_{1-x}Bpy_x$-CPd COFs.

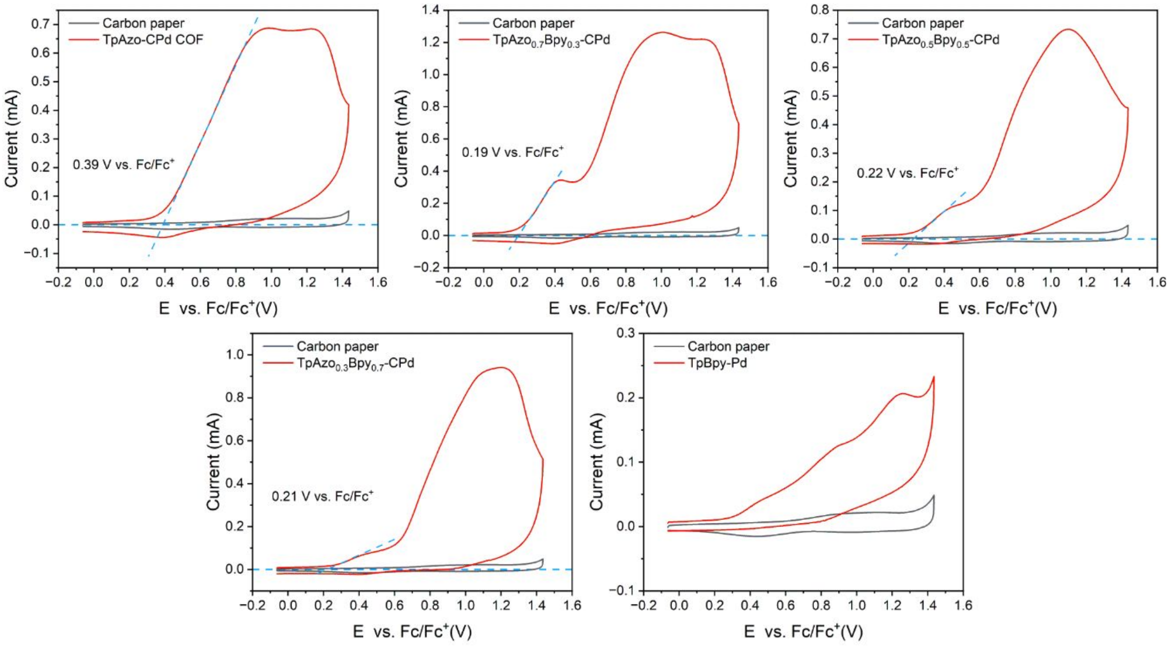

Figure S33. Cyclic voltammogram and anodic onset potential extracted of $TpAzo_{1-x}Bpy_x$-CPd COFs on carbon paper (bare carbon paper is shown as control).

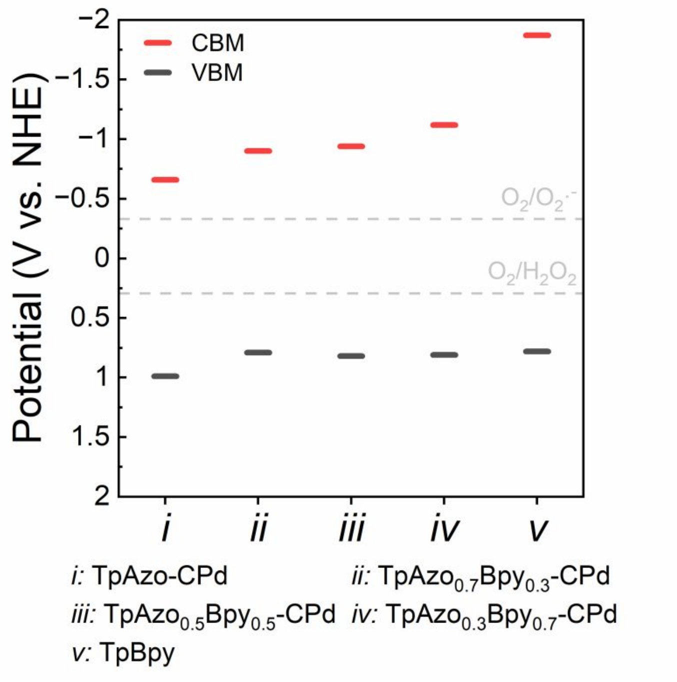

Figure S34. Energy levels of the COFs vs. Normal Hydrogen Electrode (NHE).

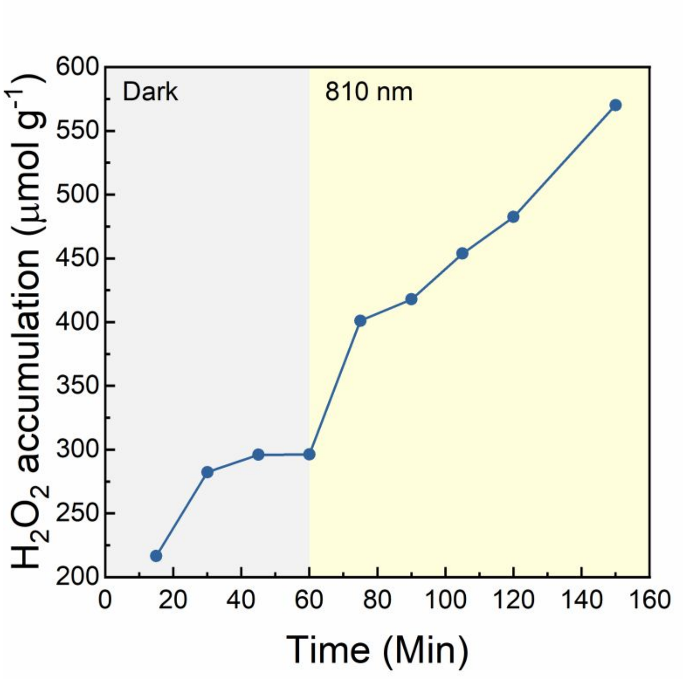

Figure S35. Photocatalytic H_2O_2 production of $TpAzo_{0.5}Bpy_{0.5}$-CPd COF under dark (grey shaded area) and 810 nm LED illumination (yellow shaded area).

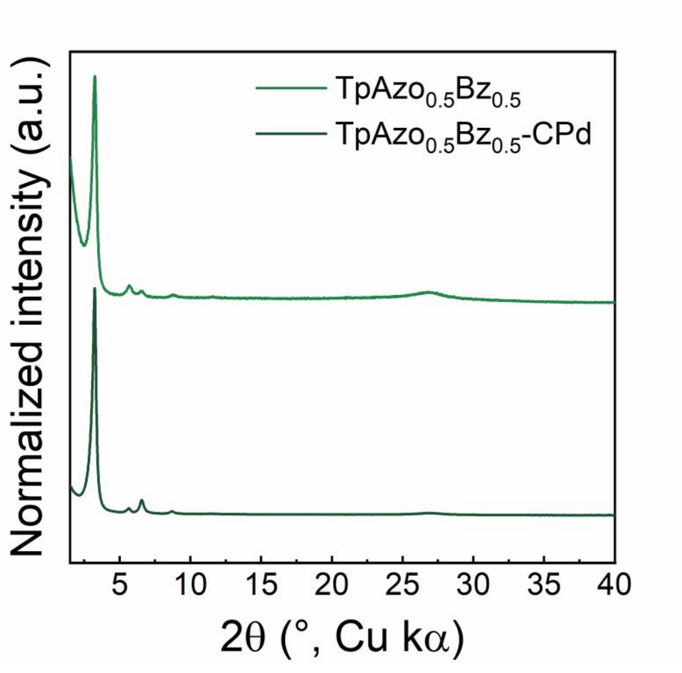

Figure S36. PXRD patterns of $TpAzo_{0.5}Bz_{0.5}$ and $TpAzo_{0.5}Bz_{0.5}$-CPd COFs.

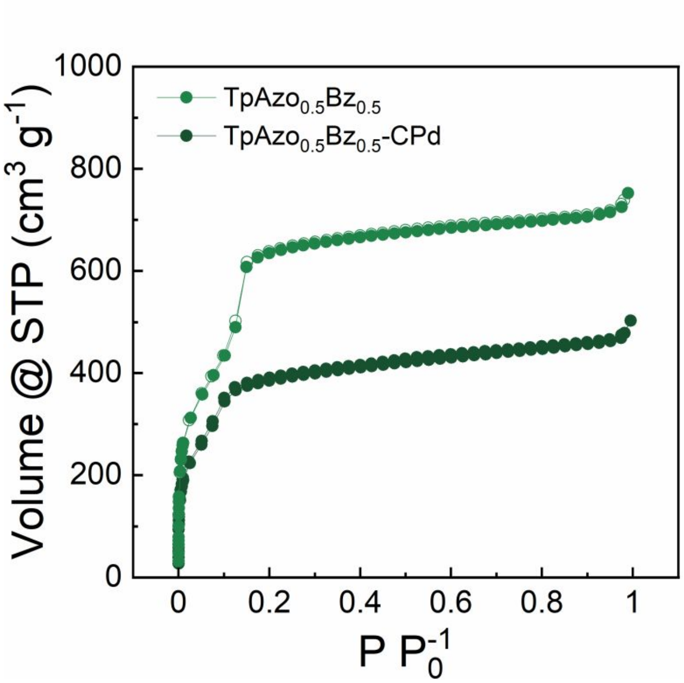

Figure S37. N_2 sorption isotherms of TpAzo$_{0.5}$Bpy$_{0.5}$ and TpAzo$_{0.5}$Bpy$_{0.5}$-CPd COFs (solid dots represent the adsorption and empty dots represent the desorption).

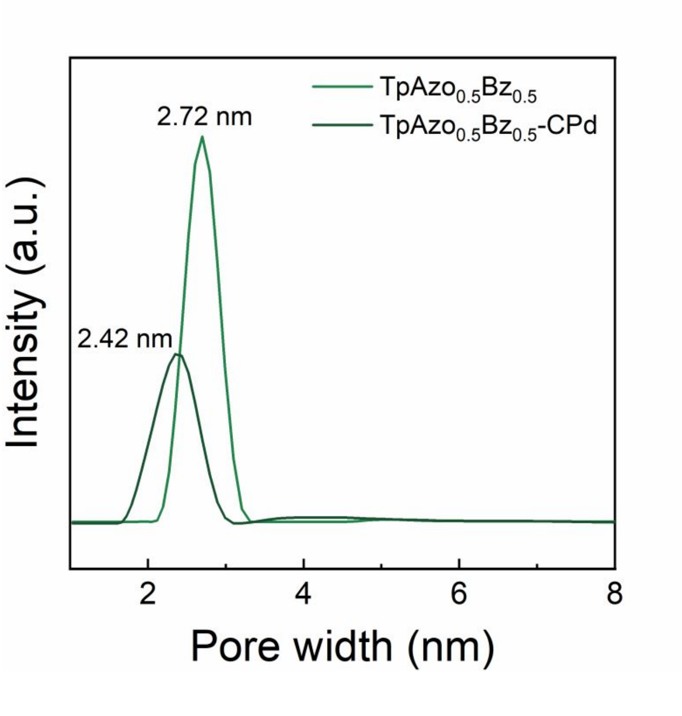

Figure S38. Pore size distribution of TpAzo$_{0.5}$Bpy$_{0.5}$ and TpAzo$_{0.5}$Bpy$_{0.5}$-CPd COFs.

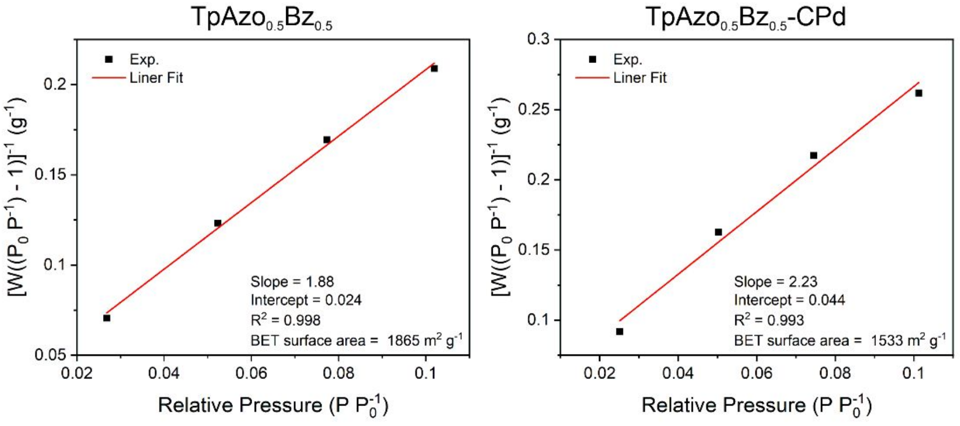

Figure S39. BET plots of $TpAzo_{0.5}Bpy_{0.5}$ and $TpAzo_{0.5}Bpy_{0.5}$-CPd COFs and their linear fitting.

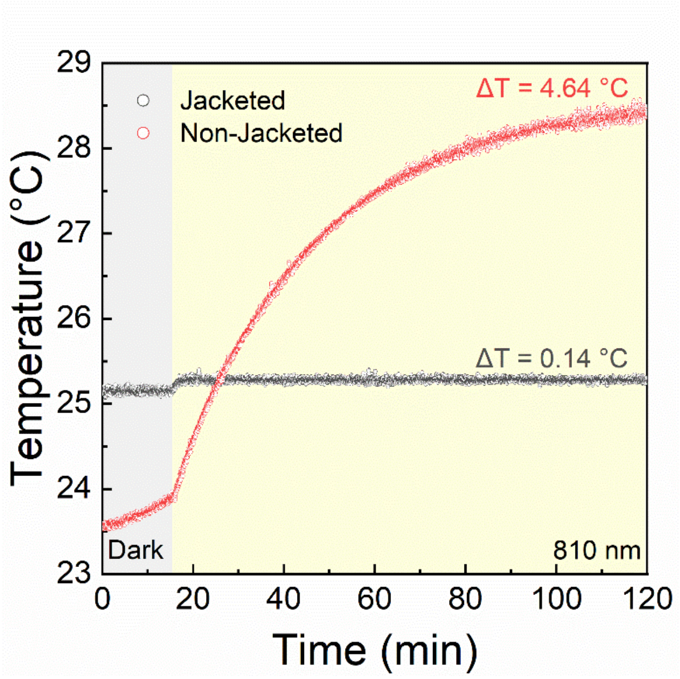

Figure S40. Temperature changes after irradiation inside a jacketed and non-jacketed photoreactor. For the experiments, a previously described reactor was used with a continuous water flow at 25.0 °C for the jacketed case and a jacket open to the air in the unjacketed case.

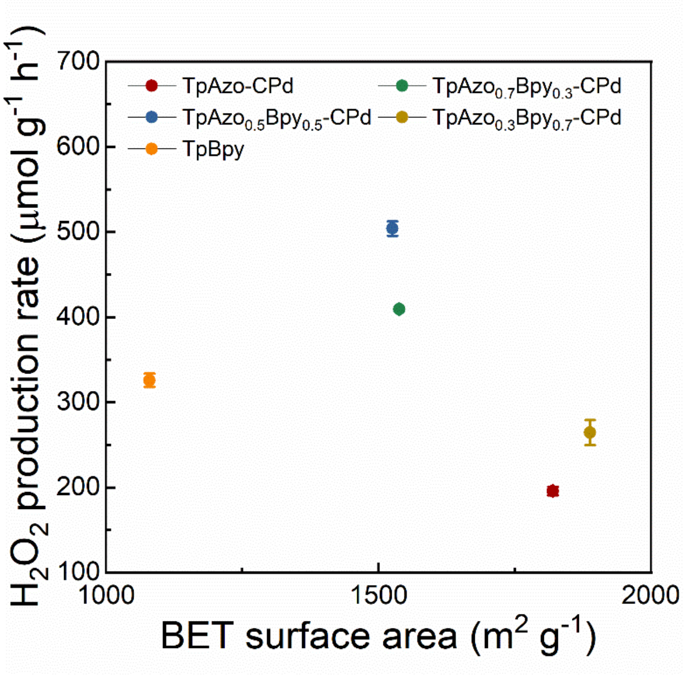

Figure S41. Correlation between calculated BET surface area and H_2O_2 production rate of different COFs.

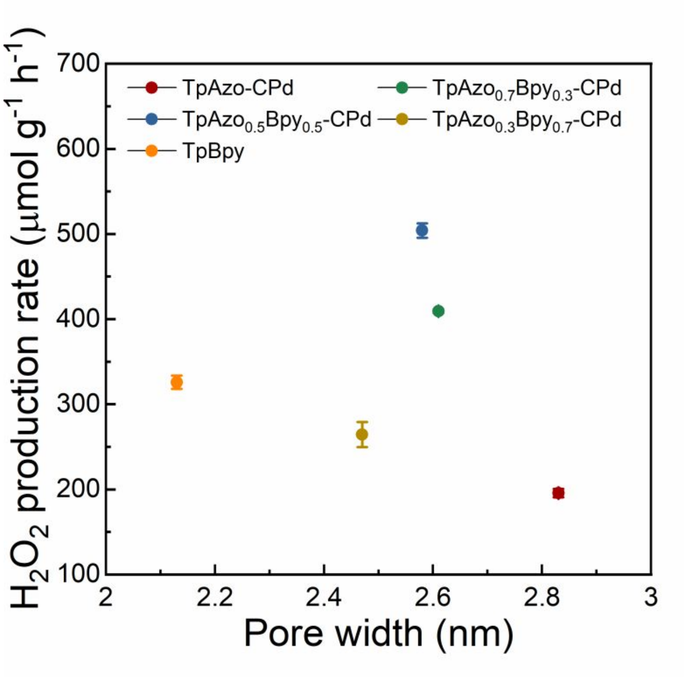

Figure S42. Correlation between calculated pore width and H_2O_2 production rate of different COFs.

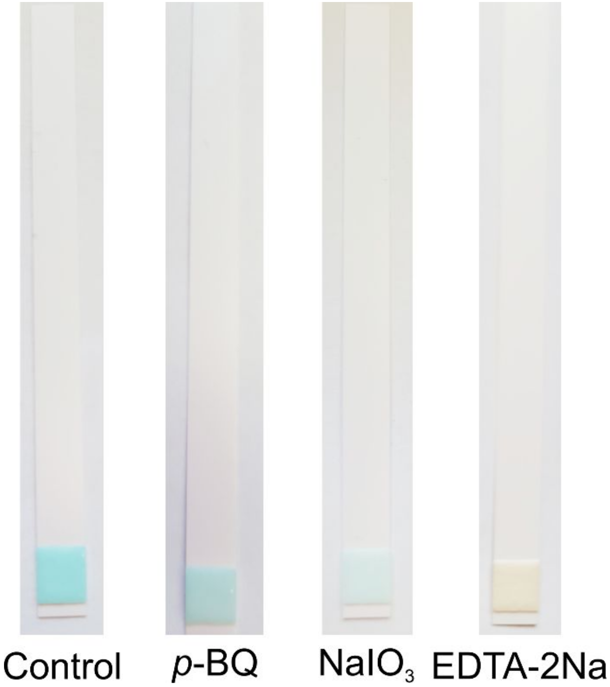

Figure S43. Qualitative determination of H_2O_2 by test strips after 1-hour illumination (810 nm LED) of $TpAzo_{0.5}Bpy_{0.5}$-CPd COF with different scavengers.

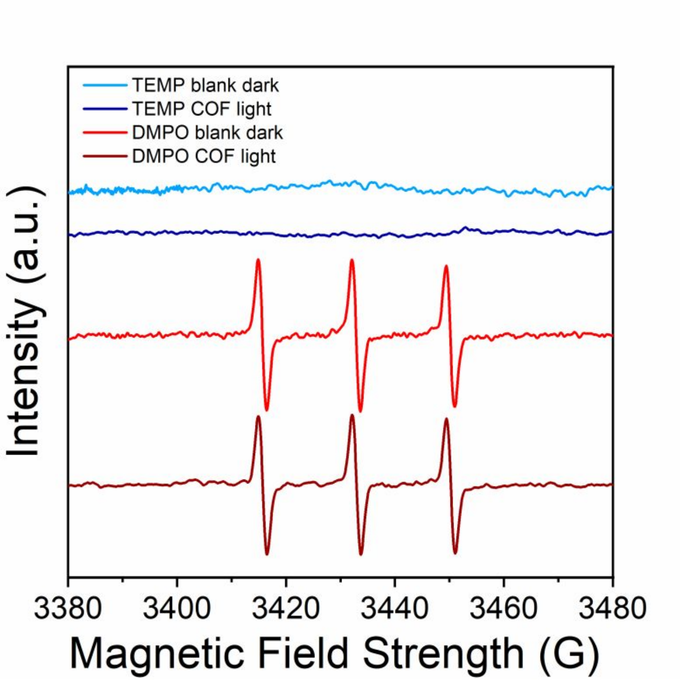

Figure S44. EPR spectra of radical scavenger experiments for $TpAzo_{0.5}Bpy_{0.5}$-CPd COF.

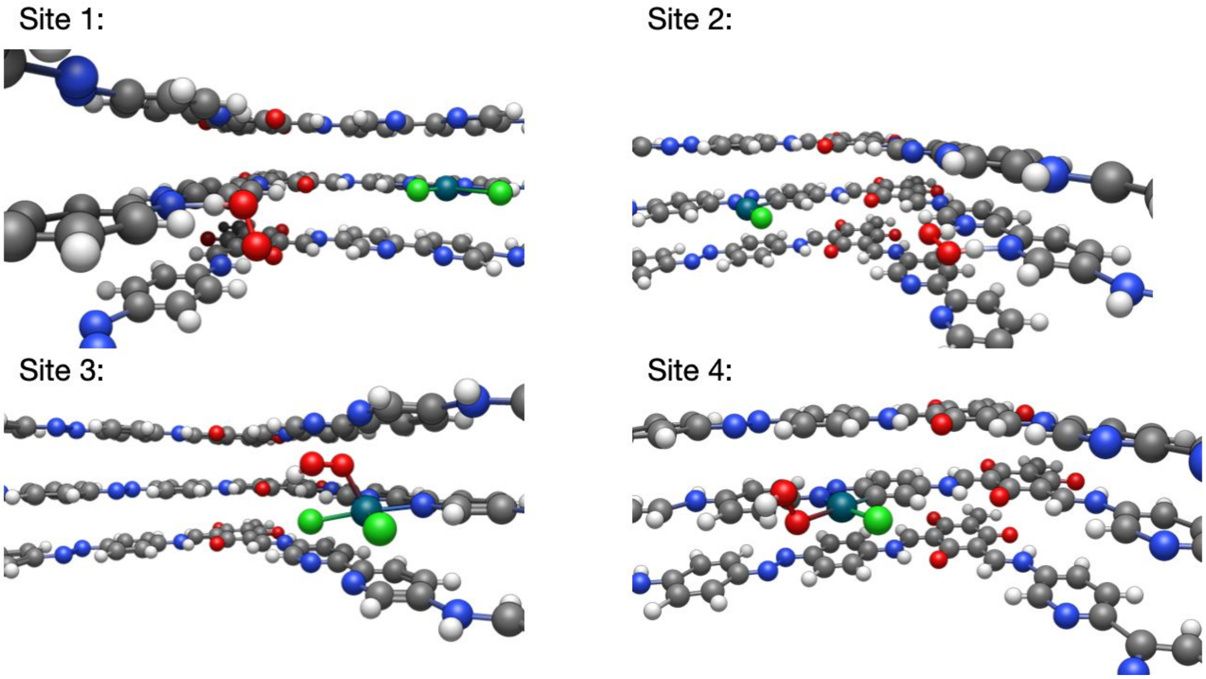

Figure S45. Proposed reactive sites for TpAzo0.5Bpy00.5-CPd COF. Three layer models were used in the quantum-chemical calculations. In gray, carbon atoms; white, hydrogen; red, oxygen; blue, nitrogen; dark blue, palladium and green, chlorine.

Table S6. Z-potential of for $TpAzo_{0.5}Bpy_{0.5}$-CPd COF in 10 mM NaCl aqueous solution.

Trial	ZP (mV)
**1**	21.3
**2**	21.7
**3**	20.1
**4**	18.4
**5**	20.6
**6**	20.5
**7**	19.4
**8**	18.5
**9**	21.7
**10**	20.8
**Average**	20.3 ± 0.4

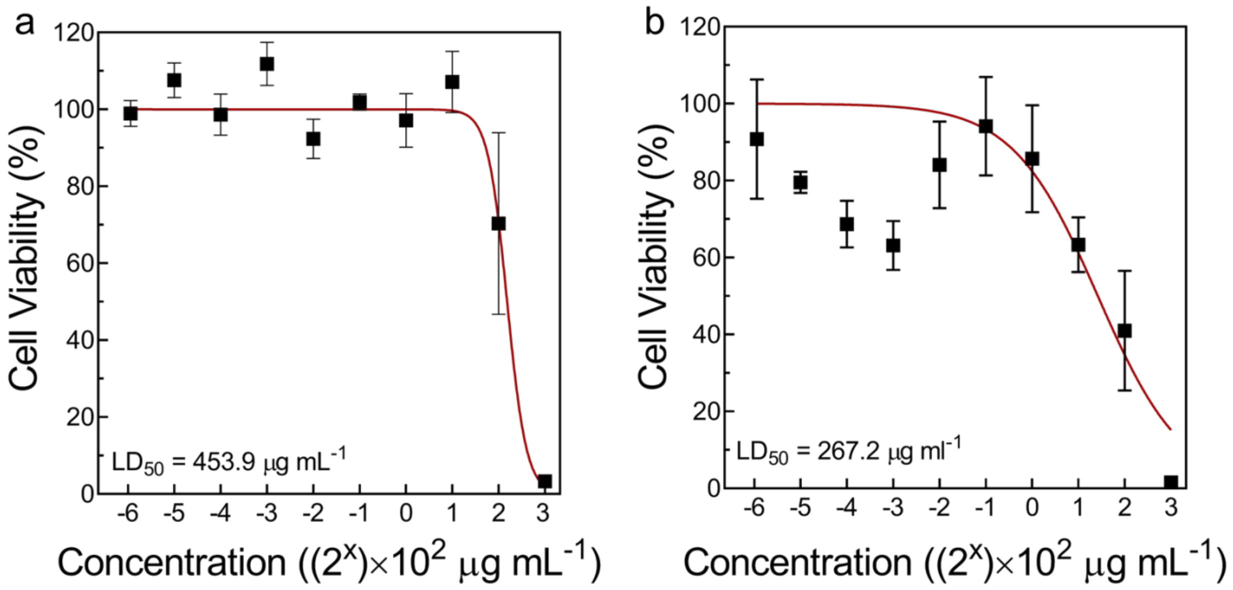

Figure S46. Luciferase-based quantitative cell viability assay results in various concentrations and estimation of the median lethal doses (LD_{50}) of $TpAzo_{0.5}Bpy_{0.5}$-CPd COF on different cell lines: (a) BJ healthy fibroblast cells and (b) SKBR3 breast cancer cells.

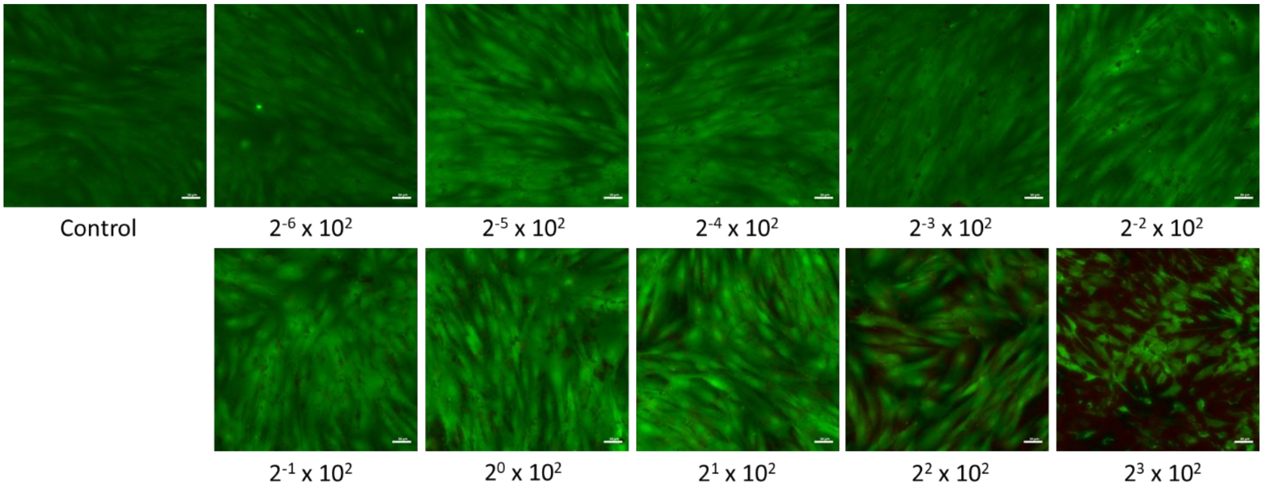

Figure S47. Calcein-AM-based fluorescent viability assay for BJ healthy fibroblast cells under various concentrations of $TpAzo_{0.5}Bpy_{0.5}$-CPd COF. The scale bar is 50 µm.

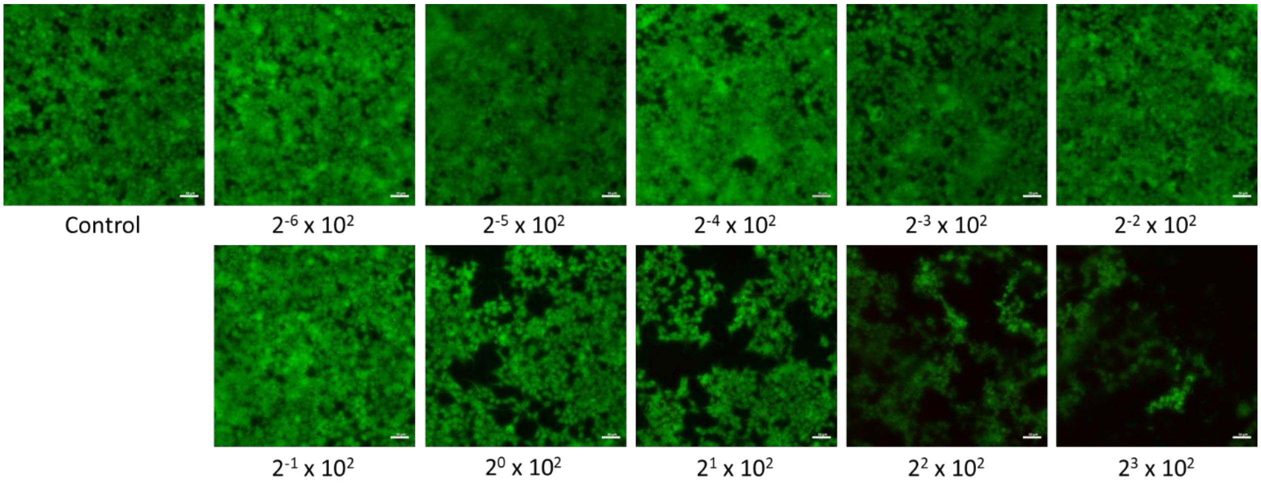

Figure S48. Calcein-AM-based fluorescent viability assay for SKBR3 breast cancer cells under various concentrations of $TpAzo_{0.5}Bpy_{0.5}$-CPd COF. The scale bar is 50 µm.

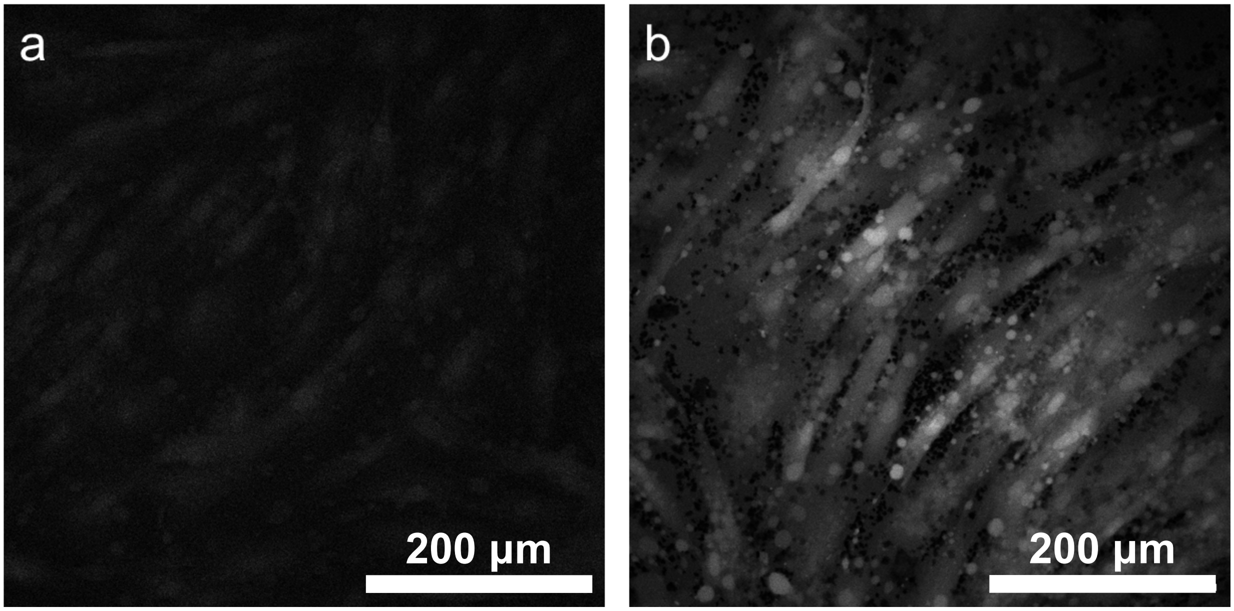

Figure S49. Fluorescent H_2DCFDA assay results for the ROS levels in a BJ fibroblast cell culture without (left) and with (right) 200 µg mL^{-1} $TpAzo_{0.5}Bpy_{0.5}$-CPd COF under 1-hour NIR light exposure.

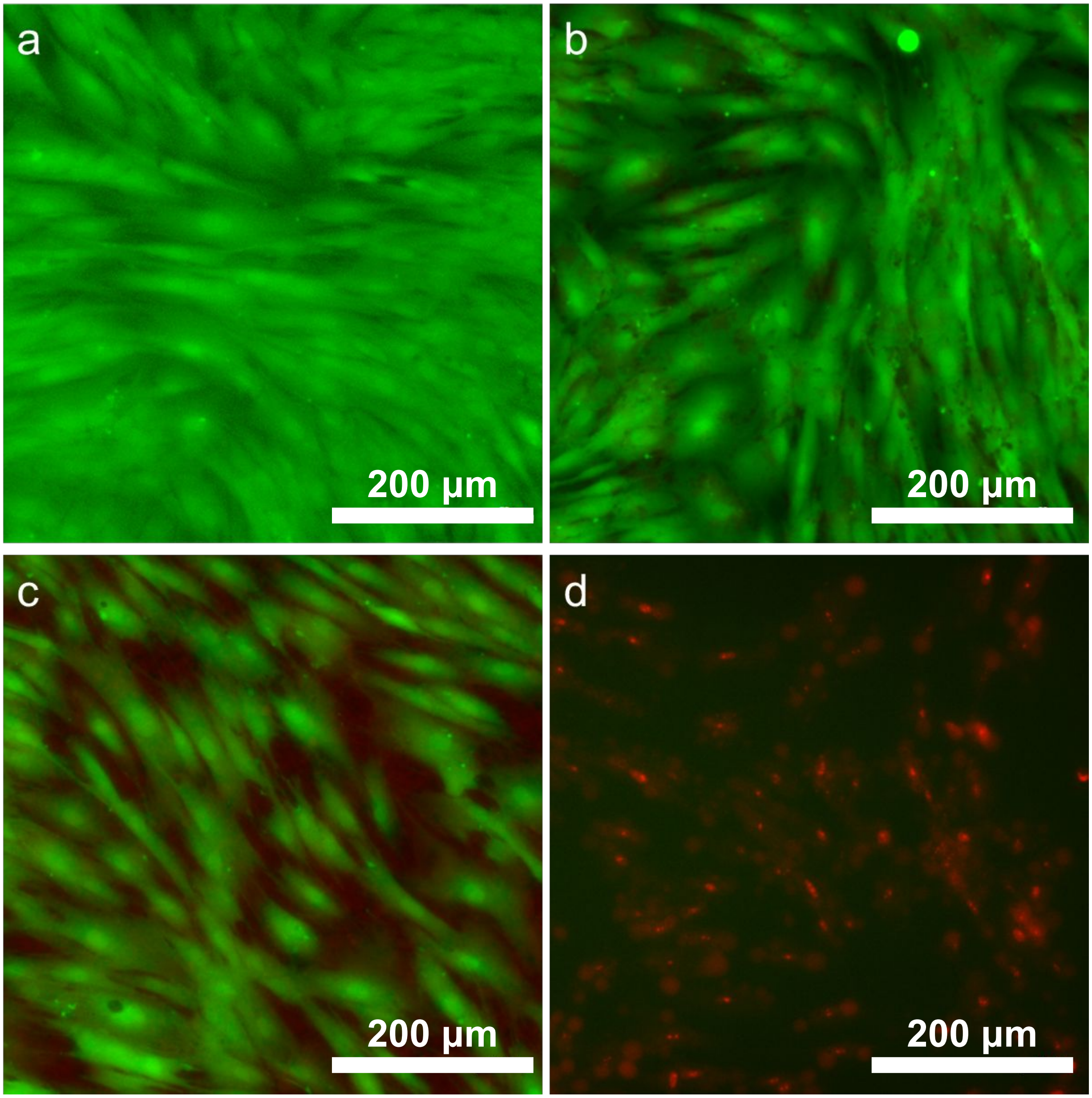

Figure S50. Calcein-AM-based fluorescent live-dead cell viability assay results for BJ fibroblast cells under various conditions: (a) Control; (b) with 200 μg mL^{-1} $TpAzo_{0.5}Bpy_{0.5}$-CPd COF, dark condition; (c) with 200 μg mL^{-1} $TpAzo_{0.5}Bpy_{0.5}$-CPd COF and NIR illumination; (d) H_2O_2 incubation. While green fluorescence indicates live cells, red fluorescence indicates dead cells.

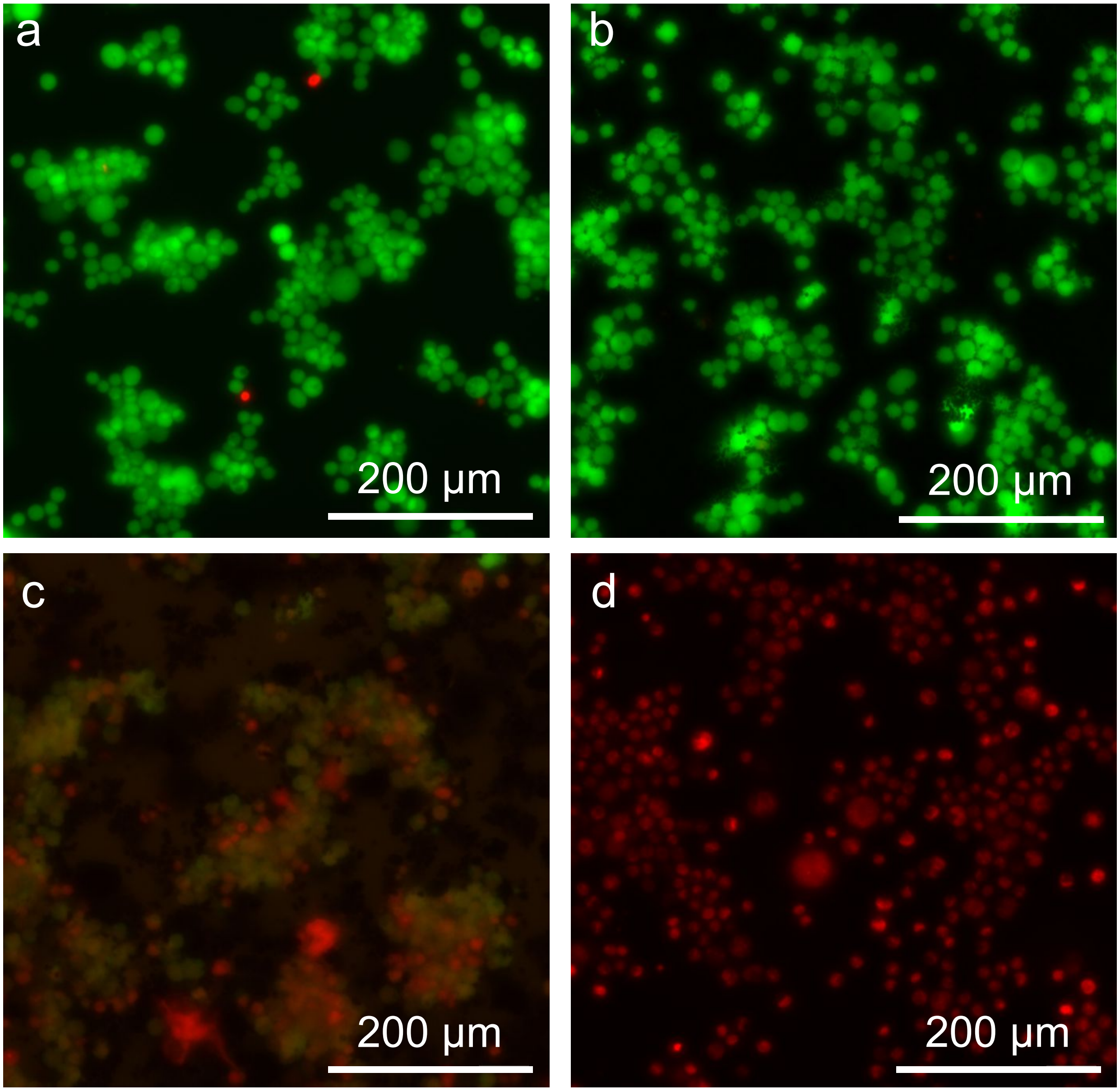

Figure S51. Calcein-AM-based fluorescent live-dead cell viability assay results for SKBR3 breast cancer cells under various conditions: (a) Control; (b) with 200 μg mL^{-1} $TpAzo_{0.5}Bpy_{0.5}$-CPd COF, dark condition; (c) with 200 μg mL^{-1} $TpAzo_{0.5}Bpy_{0.5}$-CPd COF and NIR illumination; (d) H_2O_2 incubation. While green fluorescence indicates live cells, red fluorescence indicates dead cells.

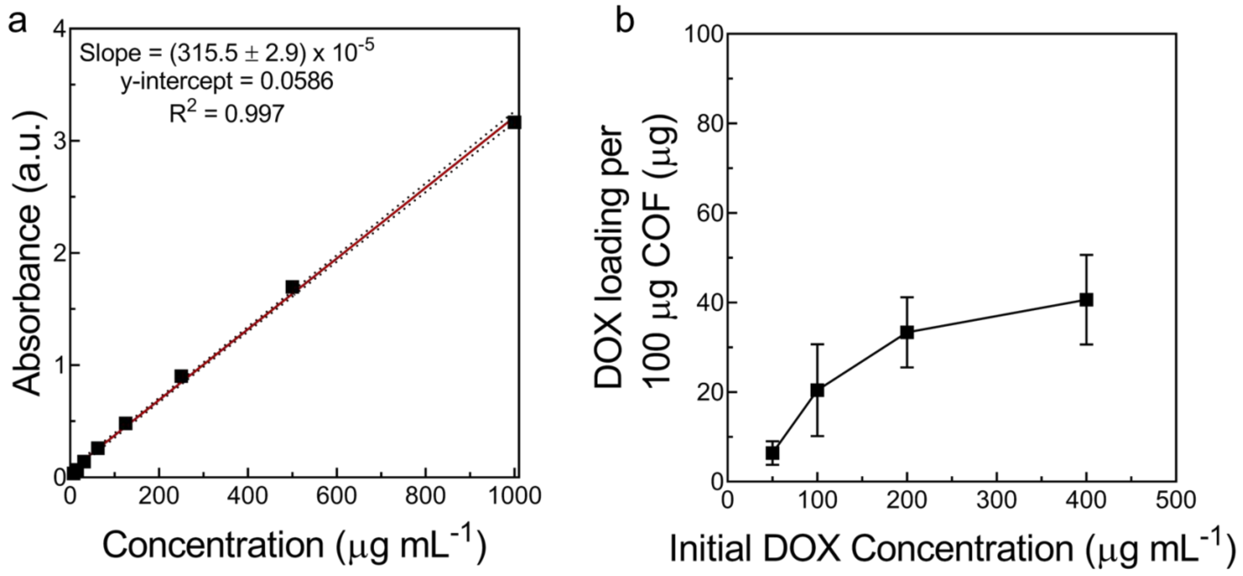

Figure S52. (a) Absorbance-concentration curve for doxorubicin (DOX) under 480 nm light illumination. **(b)** Total DOX loading in TpAzo$_{0.5}$Bpy$_{0.5}$-CPd COF using different concentrations of DOX aqueous solution for 100 μg COF.

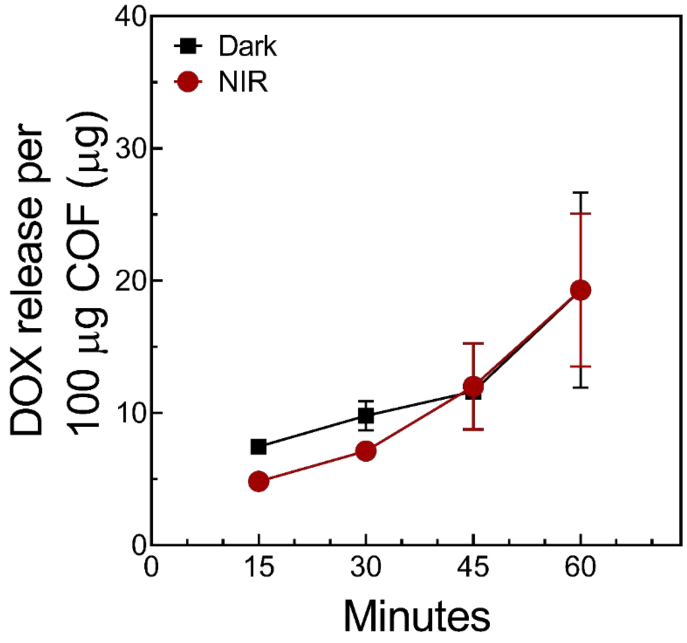

Figure S53. Measurement of DOX release from TpAzo$_{0.5}$Bpy$_{0.5}$-CPd COF in the dark and under 810 nm NIR irradiation. No significant enhancement of DOX release was observed after NIR irradiation.

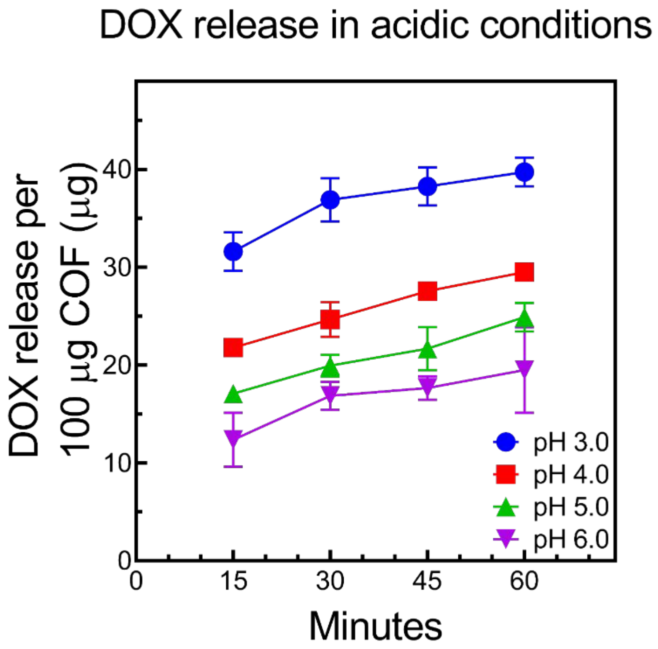

Figure S54. Measurement of DOX release in various acidic conditions from pH 3.0 to 6.0.

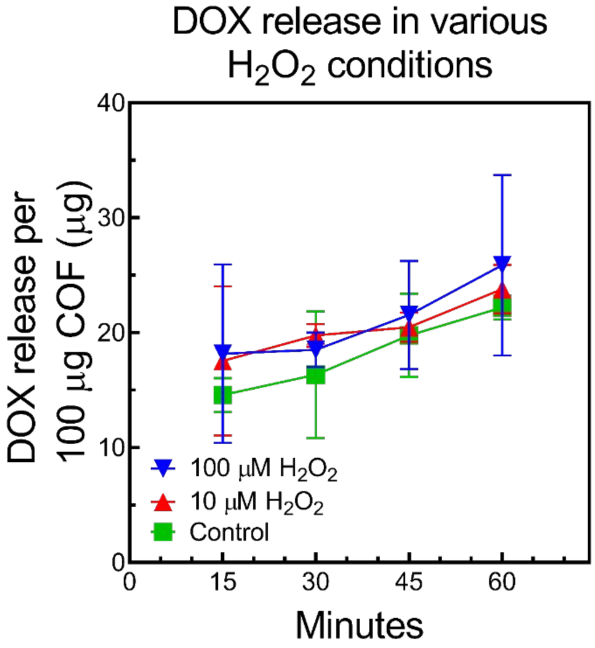

Figure S55. Measurement of DOX release in various oxidative conditions from 0 to 100 µM H_2O_2 (n=4).

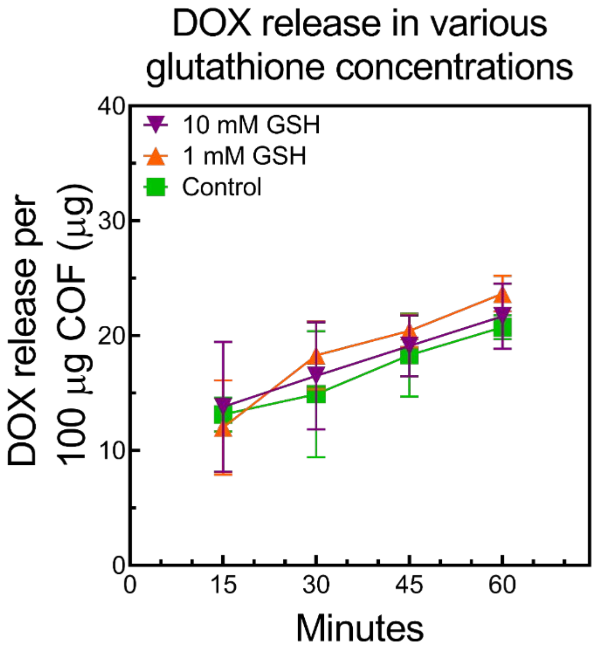

Figure S56. Measurement of DOX release in various glutathione concentrations from 0 to 10 mM glutathione (n=4).

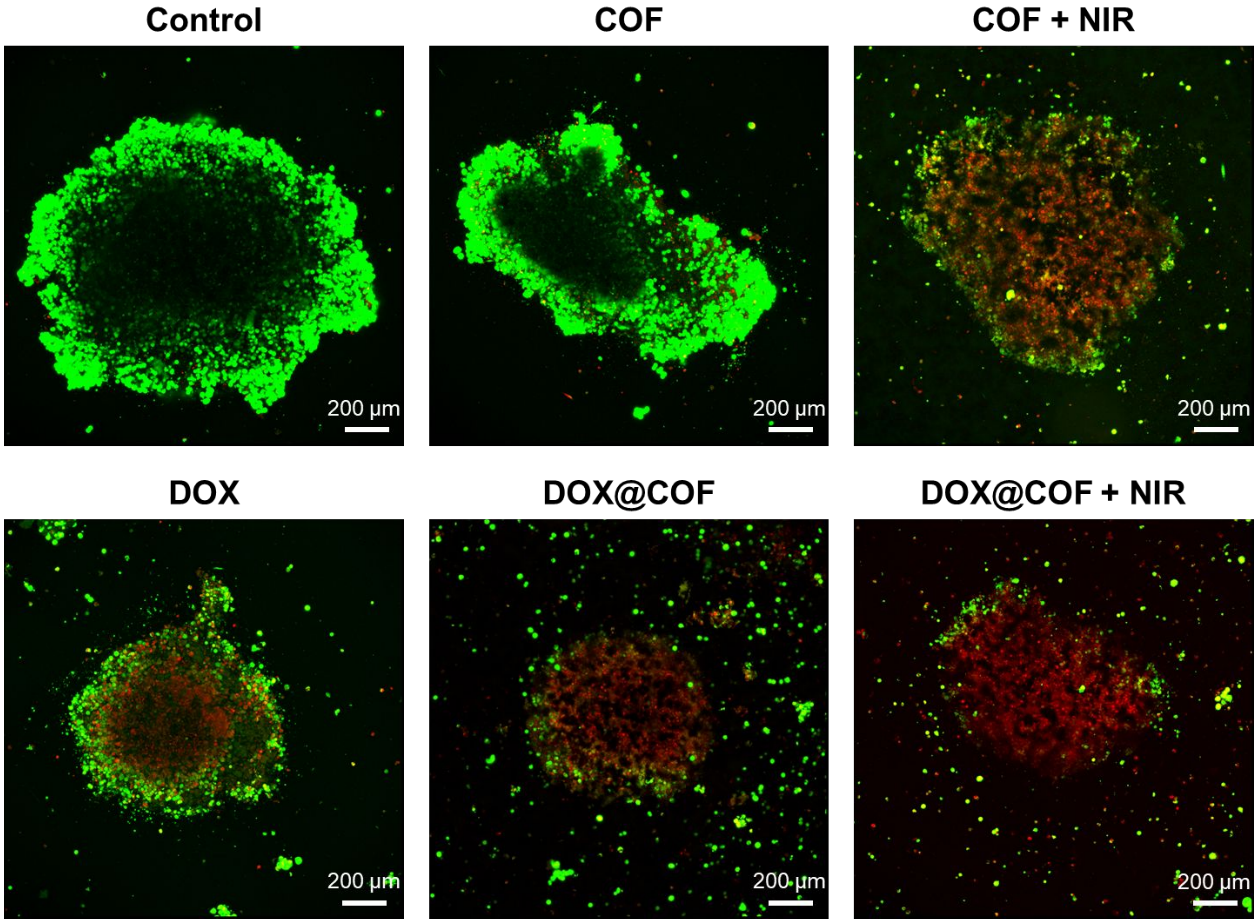

Figure S57. Calcein-based live-dead stainings of the breast cancer organoids after various treatments. While green fluorescence indicates live cells, red fluorescence indicates dead cells.

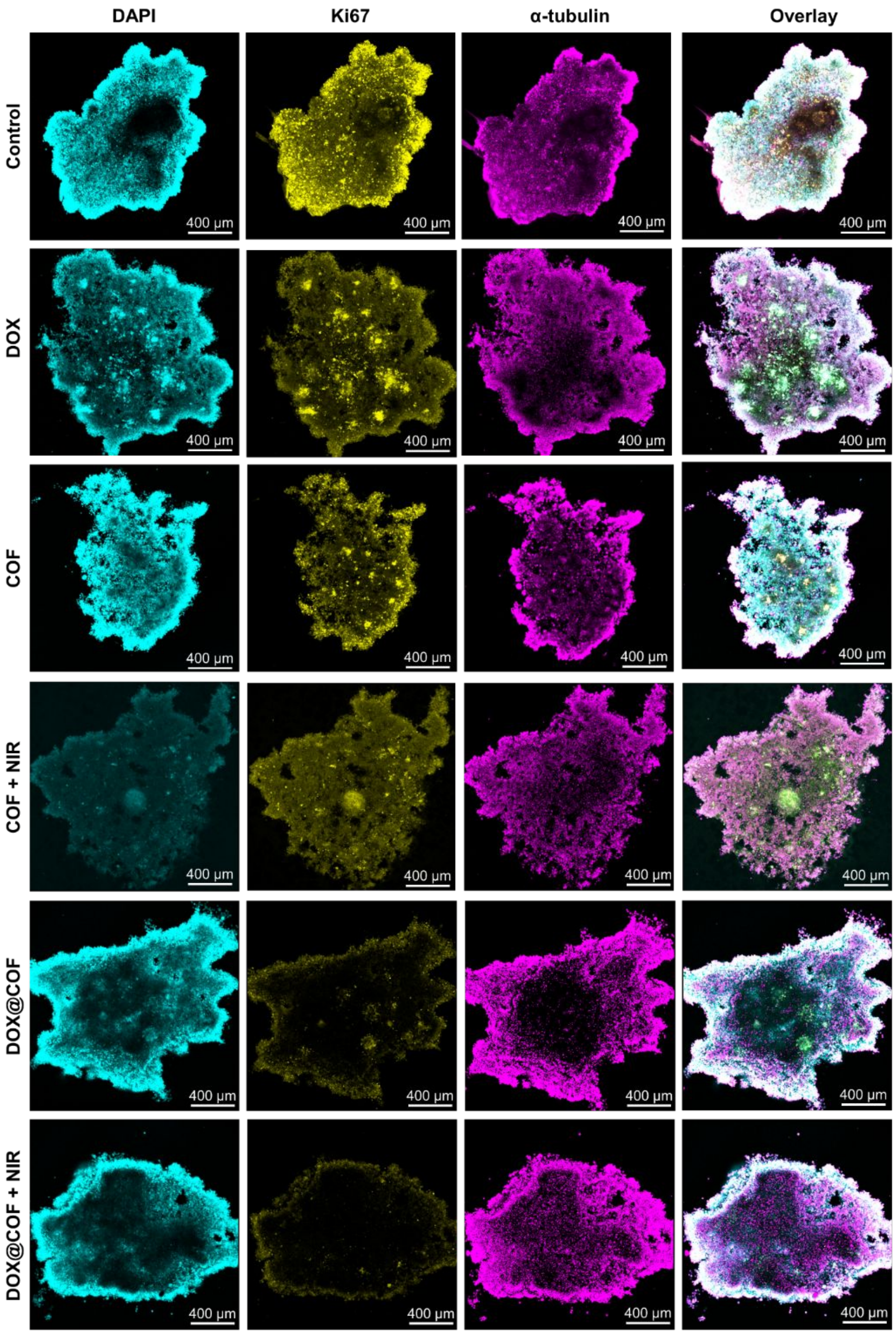

Figure S58. Immunofluorescence images of the breast cancer organoids after various treatments. The organoids are stained with DAPI (cyan) as a nucleus marker, Ki67 (yellow) as a proliferation marker, and α-tubulin (magenta) as a cytoskeleton marker. All images are collected under the same laser power, gain, and exposure. A decrease in the Ki67 indicates that organoids under NIR irradiation with COF treatment have proliferated less.

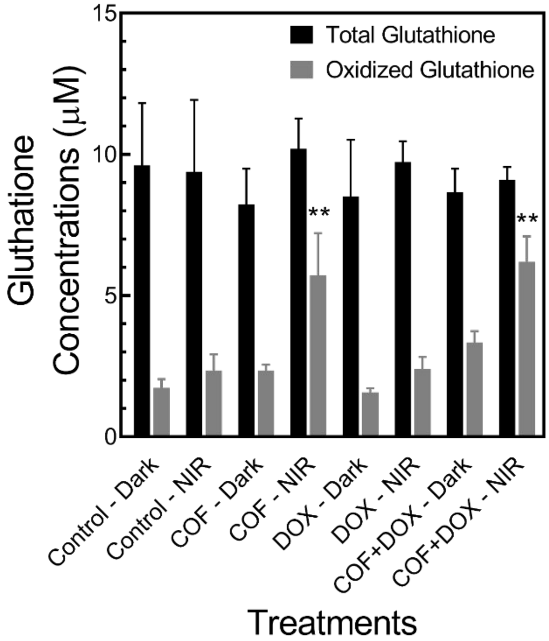

Figure S59. Oxidized and total glutathione levels in various treatments on SKBR3 cell cultures (n=6).

References

(1) Ravel, B.; Newville, M. ATHENA, ARTEMIS, HEPHAESTUS: data analysis for X-ray absorption spectroscopy using IFEFFIT. *Journal of Synchrotron Radiation* **2005**, *12* (4), 537-541. DOI: 10.1107/S0909049505012719.

(2) Yao, L.; Rodríguez-Camargo, A.; Xia, M.; Mücke, D.; Guntermann, R.; Liu, Y.; Grunenberg, L.; Jiménez-Solano, A.; Emmerling, S. T.; Duppel, V.; et al. Covalent Organic Framework Nanoplates Enable Solution-Processed Crystalline Nanofilms for Photoelectrochemical Hydrogen Evolution. *Journal of the American Chemical Society* **2022**, *144* (23), 10291-10300. DOI: 10.1021/jacs.2c01433.

(3) Liao, Q.; Sun, Q.; Xu, H.; Wang, Y.; Xu, Y.; Li, Z.; Hu, J.; Wang, D.; Li, H.; Xi, K. Regulating Relative Nitrogen Locations of Diazine Functionalized Covalent Organic Frameworks for Overall H2O2 Photosynthesis. *Angew. Chem. Int. Ed.* **2023**, *62* (41), e202310556. DOI: 10.1002/anie.202310556.

(4) Kussmann, J.; Ochsenfeld, C. Pre-selective screening for matrix elements in linear-scaling exact exchange calculations. *J. Chem. Phys.* **2013**, *138* (13). DOI: 10.1063/1.4796441.

(5) Kussmann, J.; Ochsenfeld, C. Preselective Screening for Linear-Scaling Exact Exchange-Gradient Calculations for Graphics Processing Units and General Strong-Scaling Massively Parallel Calculations. *J. Chem. Theory Comput.* **2015**, *11* (3), 918-922. DOI: 10.1021/ct501189u.

(6) Kussmann, J.; Ochsenfeld, C. Hybrid CPU/GPU Integral Engine for Strong-Scaling Ab Initio Methods. *J. Chem. Theory Comput.* **2017**, *13* (7), 3153-3159. DOI: 10.1021/acs.jctc.6b01166.

(7) Kästner, J.; Carr, J. M.; Keal, T. W.; Thiel, W.; Wander, A.; Sherwood, P. DL-FIND: An Open-Source Geometry Optimizer for Atomistic Simulations. *J. Phys. Chem. A* **2009**, *113* (43), 11856-11865. DOI: 10.1021/jp9028968.

(8) Grimme, S.; Brandenburg, J. G.; Bannwarth, C.; Hansen, A. Consistent structures and interactions by density functional theory with small atomic orbital basis sets. *J. Chem. Phys.* **2015**, *143* (5). DOI: 10.1063/1.4927476.
(9) Klamt, A.; Schüürmann, G. COSMO: a new approach to dielectric screening in solvents with explicit expressions for the screening energy and its gradient. *J. Chem. Soc., Perkin Trans. 2* **1993**, (5), 799-805. DOI: 10.1039/P29930000799.
(10) Hanwell, M. D.; Curtis, D. E.; Lonie, D. C.; Vandermeersch, T.; Zurek, E.; Hutchison, G. R. Avogadro: an advanced semantic chemical editor, visualization, and analysis platform. *J. Cheminform.* **2012**, *4* (1), 17. DOI: 10.1186/1758-2946-4-17.
(11) Wei, Z.; Liu, M.; Zhang, Z.; Yao, W.; Tan, H.; Zhu, Y. Efficient visible-light-driven selective oxygen reduction to hydrogen peroxide by oxygen-enriched graphitic carbon nitride polymers. *Energy & Environmental Science* **2018**, *11* (9), 2581-2589. DOI: 10.1039/C8EE01316K.
(12) Biswal, B. P.; Vignolo-González, H. A.; Banerjee, T.; Grunenberg, L.; Savasci, G.; Gottschling, K.; Nuss, J.; Ochsenfeld, C.; Lotsch, B. V. Sustained Solar H2 Evolution from a Thiazolo[5,4-d]thiazole-Bridged Covalent Organic Framework and Nickel-Thiolate Cluster in Water. *Journal of the American Chemical Society* **2019**, *141* (28), 11082-11092. DOI: 10.1021/jacs.9b03243.
(13) Han, M.; Yildiz, E.; Bozuyuk, U.; Aydin, A.; Yu, Y.; Bhargava, A.; Karaz, S.; Sitti, M. Janus microparticles-based targeted and spatially-controlled piezoelectric neural stimulation via low-intensity focused ultrasound. *Nature Communications* **2024**, *15* (1), 2013. DOI: 10.1038/s41467-024-46245-4.
(14) Sridhar, V.; Yildiz, E.; Rodríguez-Camargo, A.; Lyu, X.; Yao, L.; Wrede, P.; Aghakhani, A.; Akolpoglu, B. M.; Podjaski, F.; Lotsch, B. V.; et al. Designing Covalent Organic Framework-Based Light-Driven Microswimmers toward Therapeutic Applications. *Advanced Materials* **2023**, *35* (25), 2301126. DOI: 10.1002/adma.202301126.
(15) Hofland, L. J.; van der Burg, B.; van Eijck, C. H. J.; Sprij, D. M.; van Koetsveld, P. M.; Lamberts, S. W. J. Role of tumor-derived fibroblasts in the growth of primary cultures of human breast-cancer cells: Effects of epidermal growth factor and the somatostatin analogue octreotide. *International Journal of Cancer* **1995**, *60* (1), 93-99. DOI: 10.1002/ijc.2910600114.
(16) Wang, R.; Kong, W.; Zhou, T.; Wang, C.; Guo, J. Organobase modulated synthesis of high-quality β-ketoenamine-linked covalent organic frameworks. *Chemical Communications* **2021**, *57* (3), 331-334. DOI: 10.1039/D0CC06519F.
(17) Manh, D. H.; Ngoc Nha, T. T.; Hong Phong, L. T.; Nam, P. H.; Thanh, T. D.; Phong, P. T. Determination of the crystalline size of hexagonal La1−xSrxMnO3 (x = 0.3) nanoparticles from X-ray diffraction – a comparative study. *RSC Advances* **2023**, *13* (36), 25007-25017. DOI: 10.1039/D3RA04018F.
(18) Maekawa, M.; Munakata, M.; Kitagawa, S.; Nakamura, M. Crystal Structure of (2,2′-Bipyridine)dichloropalladium(II). *Analytical Sciences* **1991**, *7* (3), 521-522. DOI: 10.2116/analsci.7.521.
